# SMARCA4 regulates the NK-mediated killing of senescent cells

**DOI:** 10.1126/sciadv.adn2811

**Published:** 2025-01-15

**Authors:** Virinder Reen, Mariantonietta D’Ambrosio, Pia Pernille Søgaard, Katie Tyson, Bryony J. Leeke, Isabelle Clément, Isabel C. A. Dye, Joaquim Pombo, Adam Kuba, Yemin Lan, Joanna Burr, Ida C. Bomann, Maria Kalyva, Jodie Birch, Sanjay Khadayate, George Young, Diane Provencher, Anne-Marie Mes-Masson, Santiago Vernia, Nicholas McGranahan, Hugh J. M. Brady, Francis Rodier, Raffaella Nativio, Michelle Percharde, Iain A. McNeish, Jesús Gil

**Affiliations:** ^1^ MRC Laboratory of Medical Sciences (LMS), Du Cane Road, London W12 0NN, UK.; ^2^ Institute of Clinical Sciences (ICS), Faculty of Medicine, Imperial College London, Du Cane Road, London W12 0NN, UK.; ^3^ Ovarian Cancer Action Research Centre, Department of Surgery and Cancer, Imperial College London, London W12 0NN, UK.; ^4^ Centre de Recherche du Centre Hospitalier de l’Université de Montréal (CRCHUM) et Institut du Cancer de Montréal, Montreal, QC, Canada.; ^5^ Département de Radiologie, Radio-oncologie et Médicine Nucléaire, Université de Montréal, Montreal, QC, Canada.; ^6^ Department of Hemato-Oncology, University Hospital and Faculty of Medicine and Dentistry Palacky University, Olomouc, Czech Republic.; ^7^ Department of Brain Sciences, Imperial College London, London, UK.; ^8^ UK Dementia Research Institute, Imperial College London, London, UK.; ^9^ Department of Life Sciences, Imperial College, London SW7 2AZ, UK.; ^10^ Cancer Genome Evolution Research Group, Cancer Research UK Lung Cancer Centre of Excellence, University College London Cancer Institute, London, UK.; ^11^ Département d’Obstétrique-Gynécologie, Université de Montréal, Montreal, QC, Canada.; ^12^ Département de Médecine, Université de Montréal, Montreal, QC, Canada.; ^13^ Instituto de Biomedicina de Valencia IBV-CSIC, Valencia 46012, Spain.; ^14^ Cancer Research UK Lung Cancer Centre of Excellence, University College London Cancer Institute, London, UK.

## Abstract

Induction of senescence by chemotherapeutic agents arrests cancer cells and activates immune surveillance responses to contribute to therapy outcomes. In this investigation, we searched for ways to enhance the NK-mediated elimination of senescent cells. We used a staggered screen approach, first identifying siRNAs potentiating the secretion of immunomodulatory cytokines to later test for their ability to enhance NK-mediated killing of senescent cells. We identified that genetic or pharmacological inhibition of SMARCA4 enhanced senescent cell elimination by NK cells. SMARCA4 expression is elevated during senescence and its inhibition derepresses repetitive elements, inducing the SASP via activation of cGAS/STING and MAVS/MDA5 pathways. Moreover, a PROTAC targeting SMARCA4 synergized with cisplatin to increase the infiltration of CD8 T cells and mature, activated NK cells in an immunocompetent model of ovarian cancer. Our results indicate that SMARCA4 inhibitors enhance NK-mediated surveillance of senescent cells and may represent senotherapeutic interventions for ovarian cancer.

## Teaser

SMARCA4 inhibition enhances NK-mediated killing of senescent cells and can be combined with cisplatin to treat ovarian cancer.

## INTRODUCTION

Senescence is a stress response that limits the replication of damaged, old, and cancerous cells. Senescent cells undergo a stable cell cycle exit accompanied by phenotypic changes such as changes in cell morphology, chromatin remodeling, reprogrammed metabolism, and the production of an immunomodulatory secretome termed the senescence-associated secretory phenotype (SASP) (*1*).

The SASP is a key mediator of the effects of senescent cells (*2*, *3*). Acute induction of senescence is a protective response able to hamper cancer progression (*4*) or fibrosis (*5*). In this context, SASP secretion complements the senescence-associated cell cycle arrest by initiating an immune surveillance response that eliminates senescent cells and helps restore tissue homeostasis (*5*, *6*). Different immune cell types, including CD4^+^ T cells (*6*), CD8^+^ T cells (*7*, *8*), macrophages, neutrophils, or natural killer (NK) cells (*5*, *9*), are involved in the immune surveillance and elimination of senescent cells (*10*). In particular, NK cells have been involved in the elimination of senescent cells in fibrosis (*5*), cancer (*11*), and aging (*12*).

When senescent cells are not eliminated by the immune system and linger in the tissue, the SASP contributes to chronic inflammation (*13*), driving aging, cancer, and many other age-related diseases (*14*). Consequently, drugs that selectively eliminate senescent cells (termed “senolytics”) have the potential to be used as therapies for a wide range of age-related diseases. In the context of cancer, senolytics exert an array of beneficial effects by eliminating senescent cells present in the tumor microenvironment (*15*) and limiting the side effects of cancer therapies (*16*). In addition, “one-two punch” therapies, which combine chemotherapy, radiotherapy, or targeted drugs able to induce senescence with senolytics, have been postulated as a promising anticancer approach (*17*).

Given that immune surveillance is the natural mechanism for eliminating senescent cells, the immune-mediated killing of senescent cells is an alternative to senolytic drugs. Immune-centric approaches include chimeric antigen receptor T cells targeting antigens present in senescent cells (*18*), senescence vaccines (*19*), or the use of immune checkpoint inhibitors (ICIs) (*20*, *21*). In addition, some targeted therapies [such as the combination of the CDK4/6 inhibitor palbociclib with the mitogen-activated protein kinase kinase inhibitor trametinib (P + T)] can trigger antitumoral responses by engaging senescence and activating immune surveillance (*11*, *22*, *23*). In Kirsten rat sarcoma virus (KRAS)-driven lung tumors, treatment with P + T enhanced senescence and the SASP, potentiating NK-mediated tumor clearance (*11*). While the same treatment is not sufficient to reduce the size of pancreatic tumors (*22*), strategies that potentiated SASP production, such as the use of EZH2 inhibitors, increased the infiltration of NK and T cells and resulted in a more complete antitumoral response (*23*).

High-grade serous ovarian cancer (HGSOC) is a cancer type with high mortality (*24*). This is, in part, caused by their late detection and chemotherapy resistance which translates into a five-year survival rate of 43% (*25*). HGSOC survival is strongly associated with immune infiltration (*26*). Despite that association, trials involving ICIs in HGSOC have produced disappointing results (*27*). One-two punch approaches that target senescence in HGSOC have reached a phase 1 clinical trial (NCT05358639) (*28*), and the induction of senescence by chemotherapeutic agents such as cisplatin has been shown to dictate the outcome in mouse models of ovarian cancer (*29*).

In this investigation, we devised genetic screens to identify small interfering RNAs (siRNAs) enhancing the NK-mediated killing of senescent cells. Among the siRNAs identified were those targeting *SMARCA4* (that encodes for a SWI/SNF complex component also referred to as *BRG1*). We show that genetic or pharmacological inhibition of SMARCA4 activates the cyclic GMP-AMP synthase (cGAS)/stimulator of interferon genes (STING) pathway inducing chemoattractant SASP factors. Moreover, we show that a SMARCA4 inhibitor synergized with cisplatin to increase antitumoral responses in an immunocompetent model of HGSOC in a way dependent on NK cell infiltration. Overall, our results indicate that SMARCA4 inhibitors could be used to enhance immune responses in the context of ovarian cancer and potentially other senescence-associated diseases.

## RESULTS

### A screen to identify siRNAs augmenting SASP induction

NK cells are a key cell type mediating immune-mediated surveillance and elimination of senescent cells. The two main factors controlling that process are the expression of NK receptors and immunomodulatory factors (as part of the SASP) by senescent cells (*5*, *30*, *31*).

**Fig. 1.**
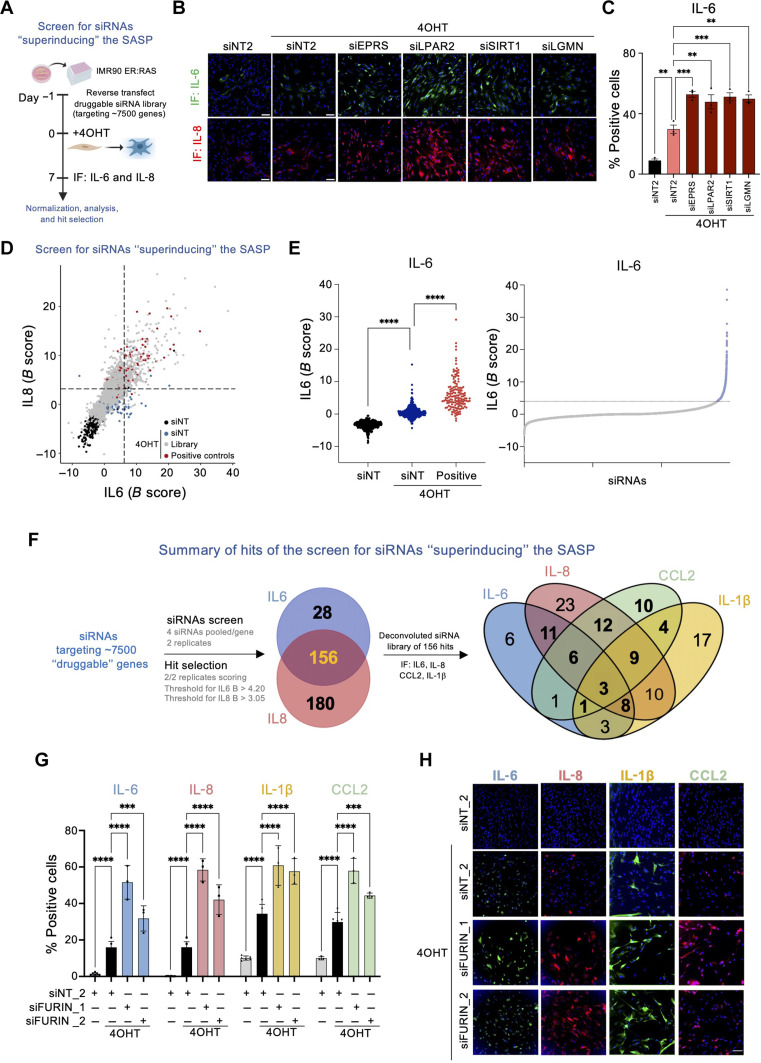
siRNA screens to identify superinducers of the SASP. (**A**) Schematic of the screen. (**B**) Representative images for IL-6 and IL-8 immunofluorescence (IF) of IMR90 ER:RAS cells transfected with the indicated siRNAs. Scale bars, 100 μm. (**C**) Percentage of IMR90 ER:RAS cells positive for IL-6 expression beyond a predetermined threshold following transfection with the indicated siRNAs. Data represent means ± SD (*n* = 3). **P < 0.01 and ***P < 0.001; ordinary one-way analysis of variance (ANOVA; Dunnett’s multiple comparisons test). (**D**) Results of the pooled siRNA screen for SASP superinducers. Normalized *B*-score values of IL-6 versus IL-8 for each replicate sample siRNA are shown. The dashed lines represent the thresholds (+2 SD of the mean of the negative non-targeting siRNA controls). Hits were selected if siRNA pools showed a normalized *B*-score value for both IL-6 and IL-8 beyond the specified threshold in both replicates. (**E**) Screen for IL-6 superinducers. Normalized score for the control siRNAs (left) and samples (right). Dotted line denotes threshold. ****P < 0.0001; ordinary one-way ANOVA (Tukey’s multiple comparisons test). (**F**) Summary of the SASP superinducer siRNA screens. The Venn diagram of the secondary screen shows the distribution of 124 hits across the indicated SASP readouts. (**G**) Percentage of IMR90 ER:RAS cells positive for the indicated SASP components 7 days after siRNA transfection. Two distinct siRNAs targeting *FURIN* are shown as well as the non-targeting control. Data represent means ± SD (*n* = 3). ***P < 0.001 and ****P < 0.0001; two-way ANOVA (Tukey’s multiple comparisons test). (**H**) Representative IF images of the indicated SASP components following transfection of siRNAs targeting *FURIN*. Scale bar, 100 μm. The results of the primary SASP siRNA screen [shown in (D) and (E)] are presented in table S1. The results of the secondary SASP siRNA screen [summarized in (F)] are presented in table S2.

To identify ways to potentiate NK-mediated killing of senescent cells, we first screened for siRNAs able to superinduce the SASP (Fig. 1A). To this end, we took advantage of IMR90 ER:RAS, a cell model widely used to study oncogene-induced senescence (OIS) (*32*). The addition of 4-hydroxytamoxifen (4OHT) to IMR90 ER:RAS cells induces senescence as assessed using multiple markers. Cells become arrested, display SA-β-galactosidase (Sa-β-Gal) activity, up-regulate the expression of cyclin-dependent kinase inhibitors, and induce the expression of the SASP (figs. S1, A to F, and S2, A and B).

We have previously used IMR90 ER:RAS cells to screen for regulators of the SASP induced 8 days after induction (*33*). That screen was better geared to identify SASP repressors, such as Polypyrimidine tract-binding protein 1 (PTBP1) (*33*). Therefore, we decided to adapt the screen by measuring the expression of interleukin-6 (IL-6) and IL-8 as readouts for the SASP 7 days after induction, a time point in which expression levels and the percentage of cells positive for IL-6 and IL-8 were lower than at day 8 (fig. S2, A and B). Transfection with siRNAs targeting known SASP regulators such as Glutamyl-Prolyl-TRNA Synthetase 1 (*EPRS*), Lysophosphatidic acid receptor 2 (*LPAR2*), Sirtuin homolog 1(*SIRT1*), or Legumain (*LGMN*), resulted in “superinduction” of IL-6 and IL-8 on senescent cells (Fig. 1B), and we could quantify the effects using automated high-throughput microscopy (Fig. 1C and fig. S2, C to E).

We screened an siRNA library targeting around 7000 druggable genes searching for those able to enhance the levels of IL-6 and IL-8 as readouts for the SASP (Fig. 1D). We calculated the percentage of cells positive for the expression of IL-6 and IL-8 and used *B* score (described in Materials and Methods) to normalize the data (fig. S2, F and G). We considered hits those siRNAs in which both replicates had a *B* ≥ 4.20 for IL-6 (Fig. 1E) or *B* ≥ 3.05 for IL-8 (fig. S2H). Overall, this primary screen allowed us to shortlist 184 genes whose knockdown resulted in superinduction of IL-6, 336 for IL-8 and 156 regulating both IL-6 and IL-8 (Fig. 1F, primary screen data presented in table S1).

To validate these results, we performed a secondary screen with a siRNA library with four siRNAs against each of the 156 candidates regulating both IL-6 and IL-8. In this secondary screen, we measured not only IL-6 and IL-8 but also two additional SASP components, IL-1β and chemokine (C-C motif) ligand 2 (CCL2) (Fig. 1F). We analyzed the screen using the normalized percent activation (NPA) method and considered hits those genes for which at least two siRNAs passed the NPA threshold (NPA ≥ 1 for IL-6 and IL-8; NPA ≥ 2 for CCL2 and IL-1β) in at least two of the three replicates (fig. S3A). Following these criteria, we identified genes whose knockdown augmented the expression of IL-6 (such as *SHH* or *3HYPDH*; fig. S3, B and C), IL-8 (such as *EIF5A* or *RFWD2*; fig. S3, D and E), CCL2 (such as *GCN1* or *MYBBP1A*; fig. S3, F and G), or IL-1β (such as *P2RX7* or *KDM5C*; fig. S3, H and I). In total, we validated 124 of the 156 candidates as increasing the expression of at least one of the SASP factors tested (Fig. 1F, secondary screen data presented in table S2). Among those candidates, the knockdown of three hits—*FURIN* (Fig. 1, G and H), *FCRL2*, and *IGBP1*—resulted in higher levels of expression of the four SASP factors tested. In summary, the described screens identified multiple candidates whose knockdown resulted in higher induction of SASP factors.

### A screen for siRNAs enhancing NK-mediated killing of senescent cells

**Fig. 2.**
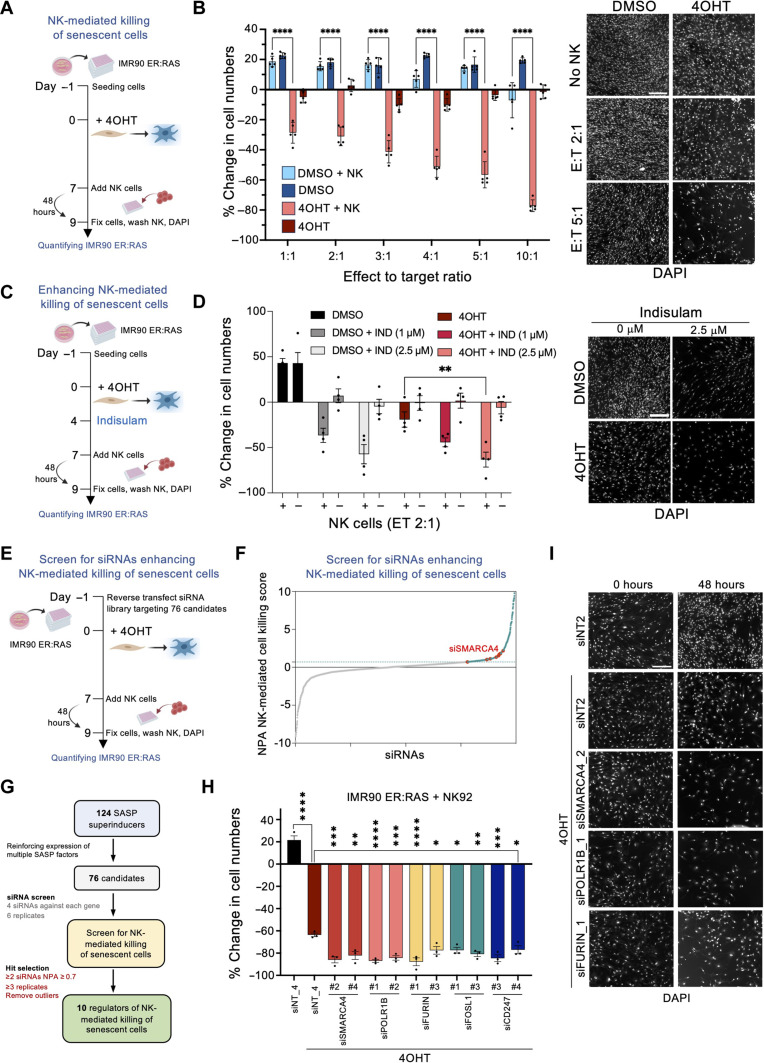
A screen for siRNAs potentiating NK-mediated killing of senescent cells. (**A**) Schematic of the coculture of IMR90 ER:RAS and NK92-MI (NK) cells. (**B**) Quantification and representative images of NK-mediated killing of IMR90 ER:RAS cells, measured as the percentage change in cell count after 48-hour coculture using 4′,6-diamidino-2-phenylindole (DAPI) staining. Control wells contained dimethyl sulfoxide (DMSO) or 4OHT-treated IMR90 ER:RAS cells only. Scale bar, 300 μm. Data represent means ± SEM (*n* = 5). ****P < 0.0001; two-way ANOVA (Tukey’s multiple comparisons test). (**C**) Schematic of the experiment testing the effect of indisulam on NK-mediated killing. (**D**) Quantification and representative images of NK coculture with control and senescent IMR90 ER:RAS cells [2:1 effector–to–target cell (E:T) ratio]. Scale bar, 300 μm. Data represent means ± SEM (*n* = 4). **P < 0.01; two-way ANOVA (Tukey’s multiple comparisons test). (**E**) Schematic of the siRNA screen to identify siRNAs potentiating NK-mediated killing of senescent cells. (**F**) Screen results showing normalized NPA scores for NK-mediated killing of senescent cells. The teal dotted line represents the cutoff (NPA ≥ 0.7). Hits were selected if at least two of the four siRNAs targeting a gene scored above the cutoff in at least three of the six replicates. siRNAs against *SMARCA4* are highlighted. Screen results are presented in table S3. (**G**) Summary workflow of the screens for siRNAs potentiating NK-mediated killing of senescent cells. (**H**) Quantification of the percentage change in IMR90 ER:RAS cell counts in the indicated conditions. Data represent means ± SEM (*n* = 3). *P < 0.05, **P < 0.01, ***P < 0.001, and ****P < 0.0001; ordinary one-way ANOVA (Dunnett’s multiple comparisons test). (**I**) Representative images of IMR90 ER:RAS cells transfected with the indicated siRNAs and cocultured with NK cells (2:1 E:T ratio) for 48 hours. Scale bar, 300 μm.

Next, we set up a high-throughput assay (described in Materials and Methods) to evaluate the killing of senescent cells by NK cells (Fig. 2A). To this end, we cocultured NK-92MI, a human immortalized NK cell line (*34*) with senescent (or control counterparts) IMR90 ER:RAS cells and evaluated NK-mediated killing by measuring the change in target cell numbers (Fig. 2B). Increasing effector–to–target cell (E:T) ratios resulted in a selective reduction of senescent cell numbers in an NK-dependent fashion (Fig. 2B). A previous report has identified indisulam as a factor enhancing the NK-mediated killing of cancer cells (*35*). To understand whether this assay could be useful in identifying factors enhancing NK-mediated killing, we carried out an experiment in which IMR90 ER:RAS cells were treated with indisulam (Fig. 2C). We observed that treatment with indisulam resulted in a dose-dependent killing of both senescent and non-senescent cells by NK cells (Fig. 2D).

To identify factors enhancing NK-mediated killing of senescent cells, we carried out a screen (Fig. 2E) taking advantage of a siRNA library with four siRNAs each against 76 candidates identified in our SASP screen (Fig. 2F). We selected 68 candidates whose knockdown resulted in the up-regulation of at least two of the four SASP factors tested and completed the library with 8 of the 10 candidates that only regulated CCL2, as CCL2 is a known chemoattract of NK cells (*31*). We analyzed the screen results using the NPA method and identified 10 genes whose knockdown in senescent cells resulted in increased NK-mediated killing (Fig. 2, F and G, and fig. S4A; data presented in table S3). We selected 5 of the 10 genes (*SMARCA4*, *POLR1B*, *FURIN*, *CD247*, and *FOSL1*) to validate the screen results. Two independent siRNAs targeting each of those genes enhanced the NK-mediated killing of senescent (Fig. 2, H and I) but not non-senescent cells (fig. S4B) without having a significant cell-intrinsic (senolytic) effect in these conditions when NK cells were not present (fig. S4C).

Next, we confirmed that siRNAs targeting *SMARCA4* (fig. S4D), *POLR1B* (fig. S4E), and *FURIN* (fig. S4F) knocked down the expression of their target genes. In summary, our screen identified *SMARCA4*, *POLR1B*, and *FURIN* among other genes whose knockdown enhanced the NK-mediated killing of senescent cells.

### Increased NK-mediated killing of senescent cells upon SMARCA4 inhibition

SMARCA4 is a component of the SWItch/Sucrose Non-Fermentable (SWI/SNF) chromatin remodeling complex (*36*). Given its relevance for cancer (*37*) and the availability of SMARCA4 inhibitors (*38*), we decided to investigate further their suitability as a target to enhance NK-mediated killing of senescent cells.

To this end, we took advantage of AU-15330, a previously described proteolysis-targeting chimera (PROTAC) able to degrade SMARCA4 and its SWI/SNF paralog SMARCA2 (*38*).

**Fig. 3.**
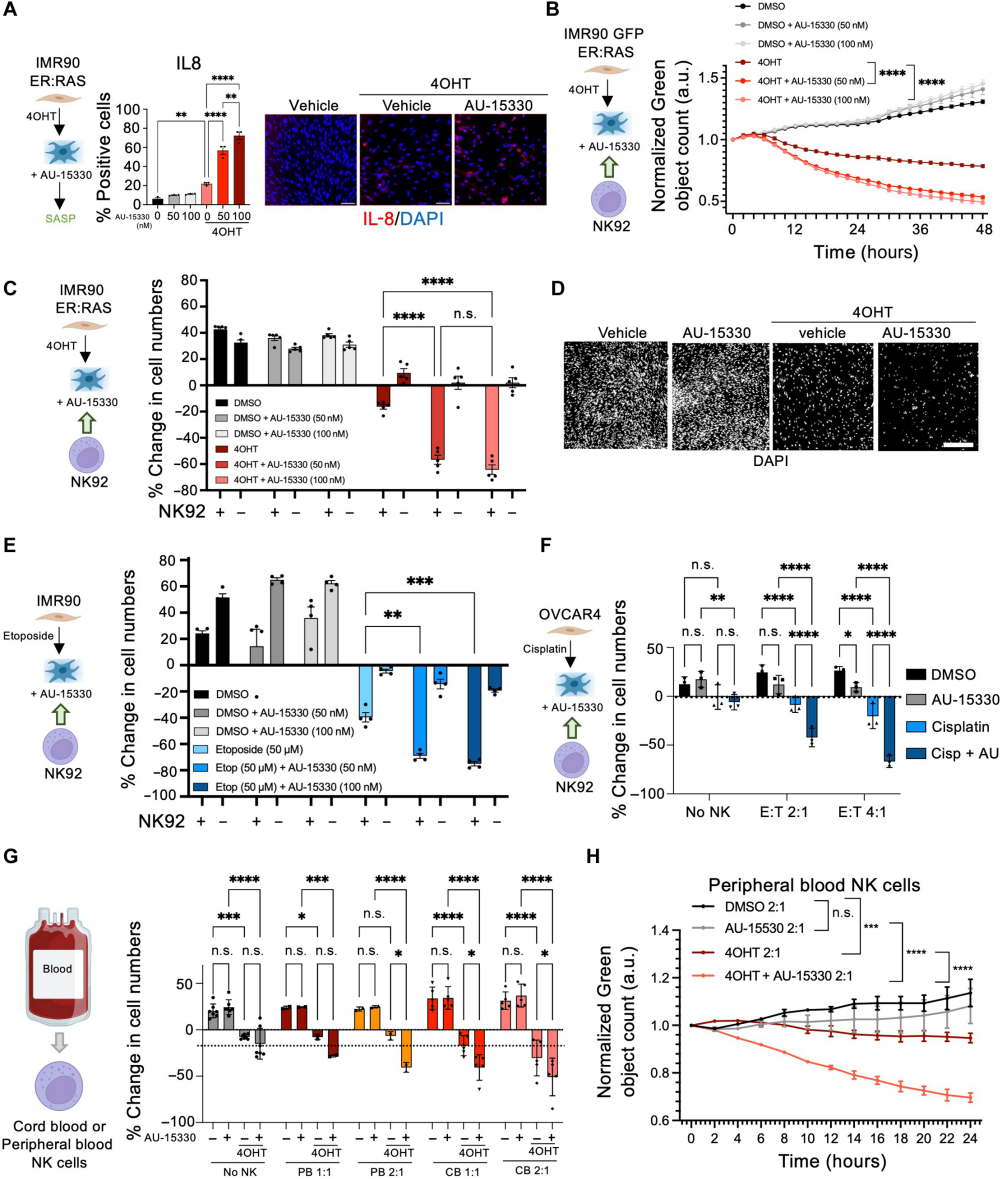
The SMARCA4 PROTAC AU-15330 potentiates NK-mediated killing of senescent cells. (**A**) Quantification and images of IL-8 expression in control and senescent IMR90 ER:RAS cells at day 7. AU-15330 was added on day 4. Scale bars, 300 μm. Data represent means ± SEM (*n* = 3). (**B**) Coculture of senescent AU-15330–treated IMR90 ER:RAS cells and NK cells at 2:1 E:T ratio. Cell numbers were measured using IncuCyte software and normalized to time 0. Data represent means ± SEM (*n* = 3). a.u., arbitrary units. (**C**) Quantification of percentage change in AU-15330–treated IMR90 ER:RAS cells cocultured with NK cells (2:1 E:T ratio). Data represent means ± SEM (*n* = 5). (**D**) Representative images of (C). Scale bar, 300 μm. (**E**) NK-mediated killing of etoposide-induced senescent AU-15330–treated IMR90 cells were cocultured with NK cells (2:1 E:T ratio). Data represent means ± SEM (*n* = 4). (**F**) NK-mediated killing of cisplatin-induced senescent AU-15330–treated OVCAR4 cells cocultured with NK cells at day 6. Data represent means ± SD (*n* = 3). (**G**) NK-mediated killing of senescent AU-15330–treated IMR90 ER:RAS cells cocultured with cord blood (CB)– or peripheral blood (PB)–derived NK cells. Data represent means ± SD. (**H**) Coculture of senescent AU-15330–treated IMR90 ER:RAS cells and peripheral blood–derived NK cells. Data represent means ± SEM. Ordinary one-way ANOVA (Tukey’s multiple comparisons test) was performed for (A), while two-way ANOVA (Tukey’s multiple comparisons test) was used in (B), (C), (E), (F), (G), and (H). n.s., not significant; *P < 0.05, **P < 0.01, ***P < 0.001, and ****P < 0.0001.

Treating IMR90 ER:RAS cells with AU-15330 showed a dose-dependent induction of SASP components such as IL-8 in senescent cells (Fig. 3A). Treatment with AU-15330 selectively increased the SASP without exacerbating or inducing senescence (fig. S5, A to F). Similar to what we observed upon SMARCA4 knockdown, treatment with AU-15330 increased the NK-mediated killing of IMR90 ER:RAS cells undergoing OIS, as assessed using live cell imaging (Fig. 3B) or end-point assays (Fig. 3, C and D). Moreover, we obtained similar results using SGC-SMARCA-BRDVIII, an inhibitor with higher affinity for the SMARCA2/4 and PB1 bromodomains (fig. S5G) (*39*).

To understand whether the increase in NK-mediated killing of senescent cells was observed beyond cells undergoing OIS, we induced therapy-induced senescence (TIS) by treating IMR90 cells with etoposide (fig. S6, A to C). Treatment with AU-15330 did not affect senescence (fig. S6, A to C) but enhanced the NK-mediated killing of IMR90 cells undergoing senescence after treatment with etoposide (Fig. 3E and fig. S6D). A similar result was observed after treating IMR90 cells undergoing TIS with SGC-SMARCA-BRDVIII (fig. S6E). Next, we induced senescence by treating the ovarian cancer cell line OVCAR4 with cisplatin (fig. S6F). Treatment with AU-15330 also served to increase the NK-mediated killing of senescent OVCAR4 cells (Fig. 3F).

All the previous experiments were performed using NK-92MI cells, which are immortalized but retain most properties of human NK cells (*34*). To investigate whether AU-15330 can also promote the killing of senescent cells by primary human NK cells, we purified NK cells from either cord blood or peripheral blood (Fig. 3G). Taking advantage of these, we confirmed that AU-15330 could also potentiate the NK-mediated killing of senescent cells by primary human NK cells (Fig. 3, G and H).

To understand whether the increased killing of senescent cells by NK in response to SMARCA4 inhibition was due only to the effects of the inhibitor in the target senescent cells, we conducted coculture experiments in which we either treated only the target senescent cells with the SMARCA4 inhibitors or also the NK cells (before and/or during the coculture; fig. S7A). These experiments suggest that the observed effects are exclusively due to SMARCA4 inhibition on the senescent target cells (fig. S7B). Nevertheless, treatment of either NK-92MI human (fig. S7C) or mouse (fig. S7D) NK cells with AU-15330 resulted in increased expression of different activation markers, suggesting that SMARCA4 inhibitors could also directly affect NK cell function. Overall, the above results show that genetic or pharmacological inhibition of SMARCA4 potentiates the NK-mediated killing of a wide range of senescent cells.

### Regulation of NK-mediated killing of senescent cells by SWI/SNF

**Fig. 4.**
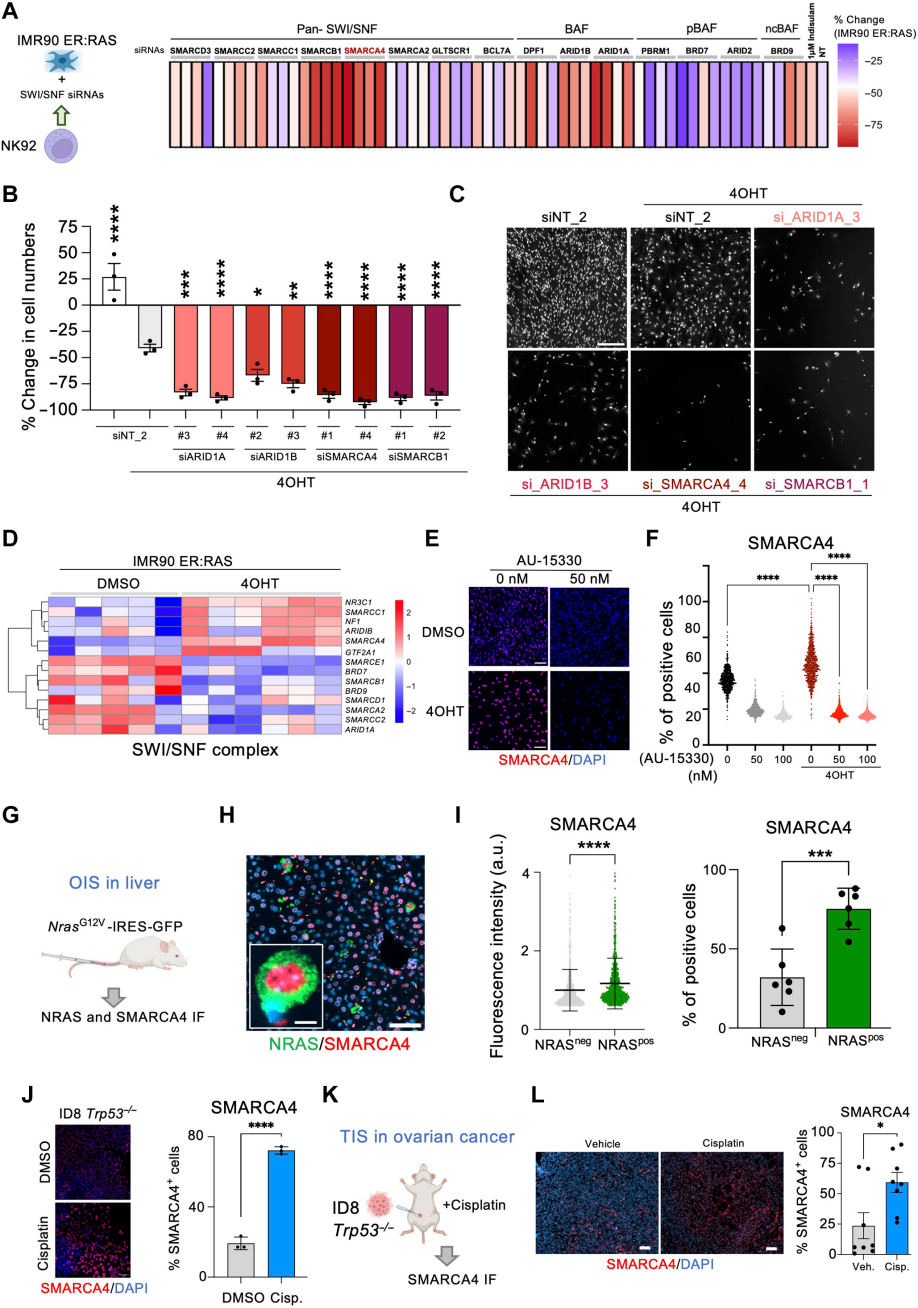
SMARCA4 induction in senescence renders SWI/SNF a target for NK cell–mediated killing. (**A**) Heatmap representing average (of *n* = 3 replicates) NK-mediated killing of senescent IMR90 ER:RAS cells transfected with indicated siRNAs. (**B**) Percentage change in cell numbers of senescent IMR90 ER:RAS cells transfected with the indicated siRNAs following coculture with NK cells at a 2:1 E:T ratio. Data represent means ± SEM (*n* = 3). *P < 0.05, **P < 0.01, ***P < 0.001, and ****P < 0.0001; ordinary one-way ANOVA (Dunnett’s multiple comparisons test). (**C**) Representative images from (B). Scale bar, 300 μm. (**D**) Heatmap of RNA sequencing (RNA-seq) data showing a SWI/SNF signature. (**E**) IMR90 ER:RAS cells were treated with AU-15330 on day 4. Representative IF images at day 7. Scale bars, 100 μm. (**F**) Quantification of IMR90 ER:RAS cells positive for SMARCA4 from (E). Data represent means ± SEM (*n* = 3). ****P < 0.0001; ordinary one-way ANOVA (Tukey’s multiple comparisons test). (**G**) Model of OIS in the liver. (**H**) Representative IF images of SMARCA4 and NRAS in liver samples. (**I**) SMARCA4 fluorescence intensity in NRAS^+^ cells and NRAS^−^ cells (left). A representative experiment out of the six mice (*n* = 1000 cells). Data represent means ± SD. ****P < 0.0001; unpaired *t* test. Percentage of SMARCA4-positive cells in NRAS^+^ and NRAS^−^ cells (right). Data represent means ± SD (*n* = 6). ***P < 0.001; unpaired *t* test. (**J**) SMARCA4 IF staining and quantification in ID8 *Trp53*^−/−^ cells treated with cisplatin (1 μM) or DMSO for 6 days. Scale bar, 100 μm. Data represent means ± SD (*n* = 3). ****P < 0.0001; unpaired *t* test. (**K**) Schematic of in vivo intraperitoneal injection of ID8 *Trp53*^−/−^ cells and cisplatin treatment. (**L**) SMARCA4 IF staining and quantification in omental tumors. Scale bars, 50 μm. Data represent means ± SEM (*n* = 8 mice per group). *P < 0.05; unpaired *t* test.

SMARCA4 acts as part of the chromatin-remodeler SWI/SNF complex. More than 20 different genes have been shown to form part of the SWI/SNF complex. To understand whether the immunomodulatory effects associated with SMARCA4 inhibition were shared by other members of the SWI/SNF complex, we decided to knockdown *SMARCA4* and 13 other SWI/SNF components on senescent cells and analyze how this affected NK-mediated killing (Fig. 4A). siRNAs targeting several of the SWI/SNF genes tested enhanced NK-mediated killing of senescent cells (Fig. 4, A to C). Among those were *ARID1A* and *ARID1B*, which encode for members of canonical Barrier-to-autointegration factor (BAF) complexes and siRNAs targeting *SMARCB1*, which, like *SMARCA4*, encode for a component of different types of SWI/SNF complexes (Fig. 4, A to C). Overall, these results suggest that at least canonical BAF SWI/SNF complexes mediate the immunomodulatory effects associated with SMARCA4 inhibition.

### Induction of SMARCA4 expression during senescence

To investigate the reasons explaining the selectivity toward senescent cells of the immunomodulatory effects associated with SMARCA4 inhibition, we carried out RNA sequencing (RNA-seq) analysis of IMR90 ER:RAS cells undergoing senescence. Transcriptome analysis showed that the expression of SMARCA4 and a cluster of SWI/SNF components was elevated in cells undergoing senescence (Fig. 4D). This observation was confirmed using quantitative immunofluorescence (IF) against SMARCA4 (Fig. 4, E and F). Moreover, treatment with AU-15330 served to confirm both the specificity of the antibody and the efficiency of AU-15330 in degrading SMARCA4.

To investigate whether SMARCA4 was also up-regulated during OIS in vivo, we took advantage of a system that allows for transposon-mediated expression of oncogenic NRas (NRas^G12V^) in the liver. This results in senescence induction in hepatocytes (*6*). Co-IF in liver sections of mice transduced with *NRas^G12V^* unveiled higher expression of SMARCA4 in NRAS^+^, senescent cells (Fig. 4, G to I). To extend these observations beyond OIS, we took advantage of ID8 *Trp53*^−/−^ mouse ovarian cancer cells (*40*). Treatment with cisplatin resulted in senescence induction, as assessed by reduced cell proliferation, an increase in SA-β-gal activity, and SASP induction (fig. S8, A to C). Transcriptome analysis confirmed that signatures of senescence and the SASP were up-regulated in ID8 *Trp53*^−/−^ cells (fig. S8, D and E). Quantitative IF showed that cisplatin-treated senescent ID8 *Trp53*^−/−^ cells also showed elevated levels of SMARCA4 (Fig. 4J). ID8 cells can be transplanted into syngeneic mice as a model of ovarian cancer (*40*). We injected intraperitoneally ID8 *Trp53*^−/−^ cells into C57BL/6J female mice and, when tumors appeared, treated them with either cisplatin or vehicle (Fig. 4K). Transcriptome analysis of tumors showed that cisplatin treatment resulted in senescence induction and the up-regulation of SWI/SNF-related signatures (fig. S8F). Moreover, SMARCA4 IF in sections of omental tumors also showed elevated expression of SMARCA4 upon cisplatin treatment (Fig. 4L). The above results show that SMARCA4 expression is up-regulated on senescence and might contribute to explaining the selective effects caused by SMARCA4 inhibition on senescent cells.

### Activation of cGAS/STING by SMARCA4 inhibition

**Fig. 5.**
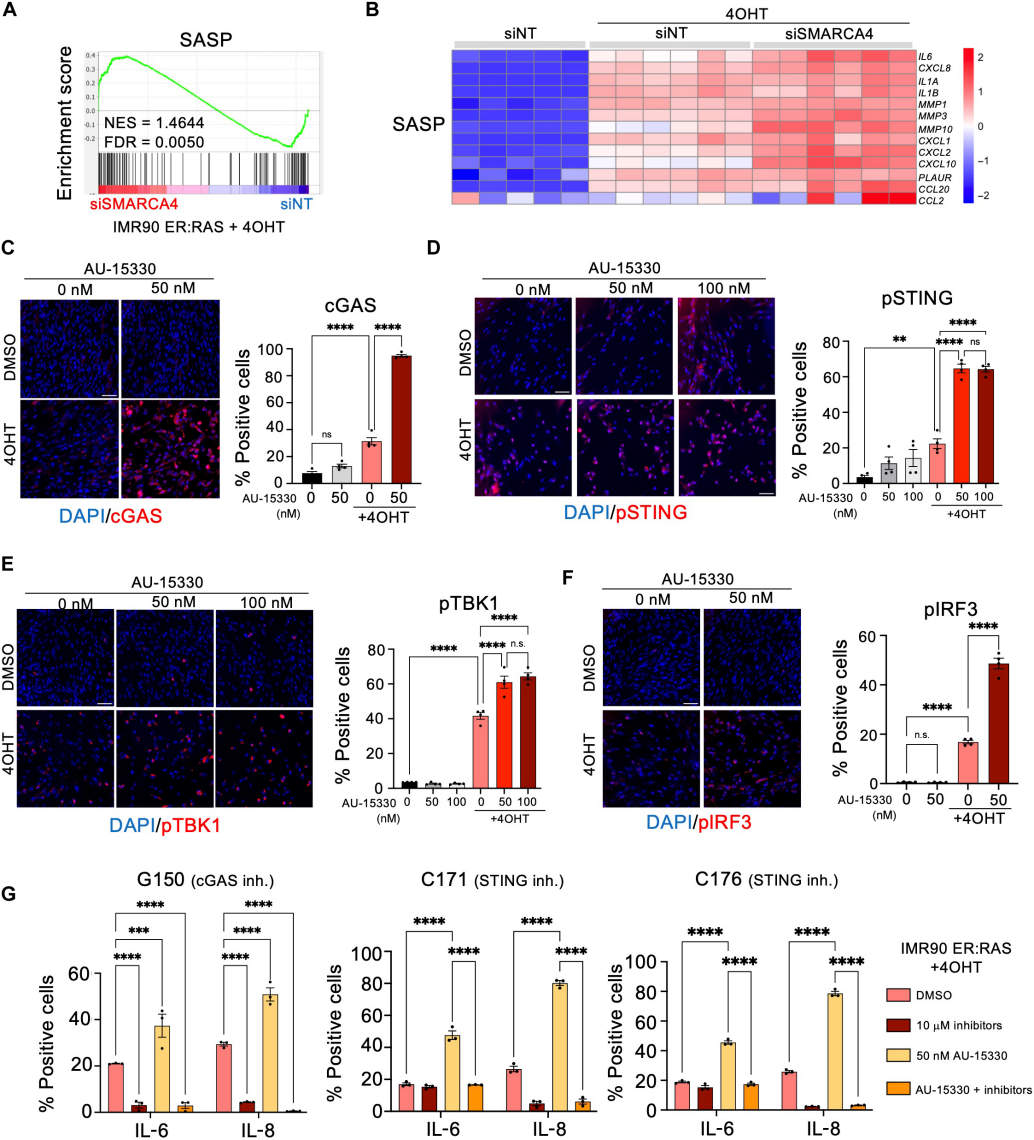
SMARCA4 inhibition potentiates NK cell–mediated senescence surveillance via the cGAS-STING pathway. (**A**) Gene set enrichment analysis (GSEA) plot of the SASP signature in 4OHT-induced IMR90 ER:RAS cells. NES, normalized enrichment score. (**B**) Heatmap of RNA-seq data showing up-regulation of SASP marker expression in senescent IMR90 ER:RAS cells transfected with siRNAs targeting *SMARCA4*. (**C** to **F**) Representative IF images (left) and quantification (right) of AU-15330–treated IMR90 ER:RAS cells positive for cGAS (C), pSTING (D), pTBK1 (E), and pIRF3 (F) staining. Scale bars, 100 μm. Data represent means ± SEM (*n* = 4). **P < 0.01 and ****P < 0.0001; ordinary one-way ANOVA (Tukey’s multiple comparisons test). (**G**) Quantification of the percentage of IL-6– and IL-8–positive 4OHT-induced IMR90 ER:RAS cells in the presence or absence of AU-15330 following treatment with the indicated inhibitors (inh.). Data represent means ± SEM (*n* = 3). ***P < 0.001 and ****P < 0.0001; two-way ANOVA (Dunnett’s multiple comparisons test).

To investigate how SMARCA4 regulates the NK-mediated killing of senescent cells, we took advantage of transcriptome profiling. Extending the results of the SASP screen, we observed that SMARCA4 knockdown caused the up-regulation of multiple SASP components (Fig. 5, A and B) that were secreted by senescent cells (fig. S9A). In contrast, *SMARCA4* knockdown did not further induce the expression of NK-activating ligands in senescent cells (fig. S9B). Gene set enrichment analysis (GSEA) also showed the up-regulation of interferon (IFN)–related signatures on senescent cells upon *SMARCA4* knockdown (fig. S9C).

The cGAS/STING pathway is a key sensor of cytoplasmic DNA with a critical role in initiating inflammation in response to a wide range of stresses, including during senescence (*41*). Transcriptome analysis showed that multiple components of the cGAS/STING pathway (fig. S9D) and signatures associated with sensing cytosolic DNA (fig. S9E) were up-regulated upon *SMARCA4* knockdown on IMR90 ER:RAS cells undergoing OIS. Moreover, similar results were observed in ID8 *Trp53*^−/−^ cells treated with cisplatin (fig. S9, F and G) and in ID8 *Trp53*−/− tumors treated with cisplatin (fig. S8F, right).

To understand whether cGAS/STING activation might explain how SMARCA4 inhibition enhanced SASP induction, we took advantage of IMR90 ER:RAS cells treated with AU-15330. Quantitative IF analysis showed that both cGAS expression and STING phosphorylation, a marker of its activation, were up-regulated in senescence and this induction was further enhanced in cells treated with AU-15330 (Fig. 5, C and D). Activation of cGAS/STING recruits and activates TBK1, initiating a signaling cascade that involves interferon regulatory factor (IRF) phosphorylation (*42*). SMARCA4 inhibition resulted in increased levels of TBK1 and IRF3 phosphorylation in senescent cells (Fig. 5, E and F). Last, we investigated the effect that inhibitors of cGAS (G150) or STING (C171 and C176) had on SASP induction upon SMARCA4 inhibition. Analysis of the expression of IL-6 and IL-8 showed how the AU-15330 increase in SASP expression was prevented upon cGAS or STING inhibition (Fig. 5G).

### The effect of SMARCA4 inhibition on the expression of TEs

cGAS behaves as a sensor for DNA that becomes activated in response to pathogen or self, cytosolic DNA (*41*). During senescence, cytoplasmic DNA derived from chromatin (known as chromatin cytoplasmic foci) (*43*, *44*) or through the mobilization of endogenous retrotransposable elements (*45*) has been suggested as signals to activate the cGAS/STING pathway.

**Fig. 6.**
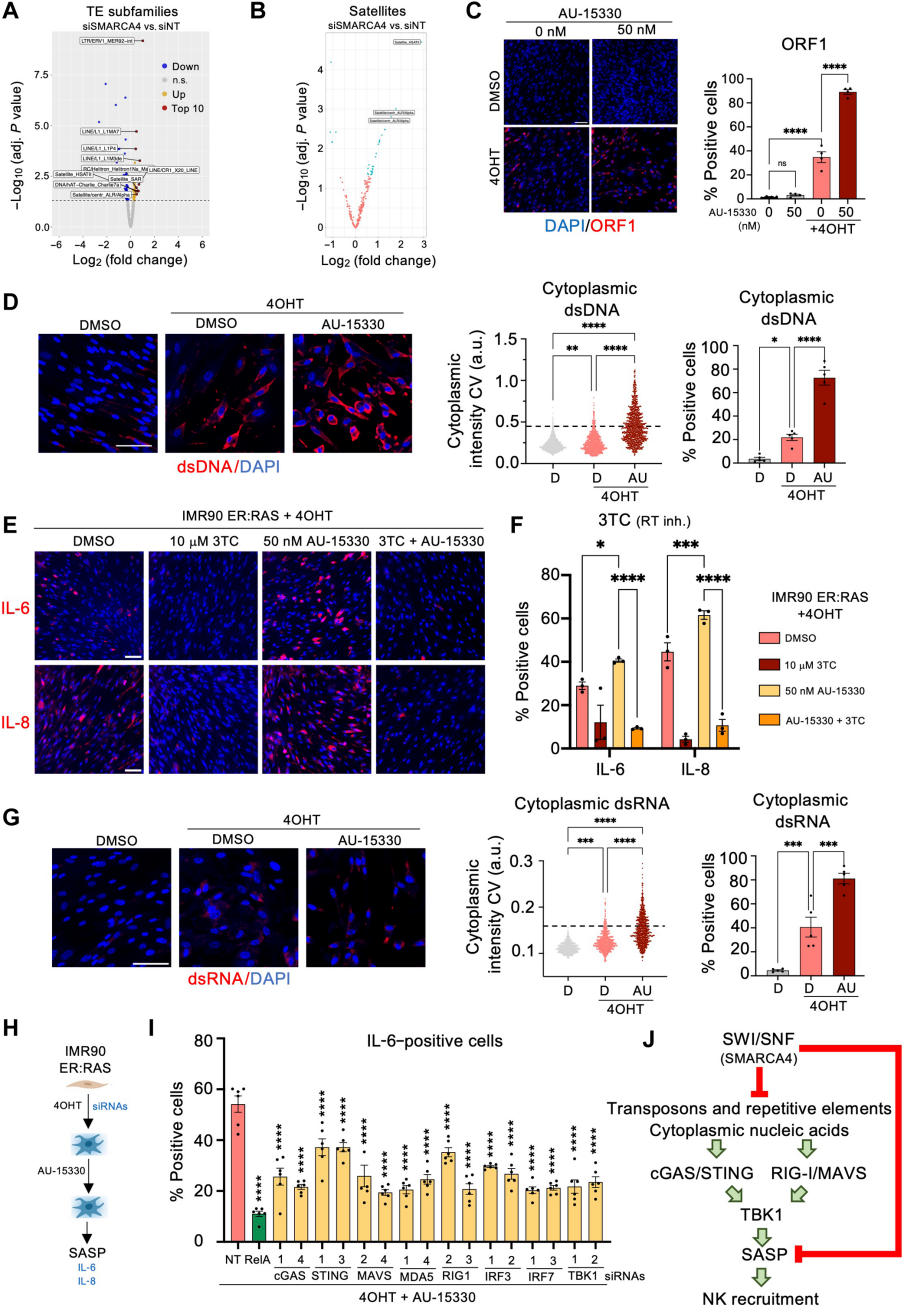
Mechanism of SASP induction upon SMARCA4 inhibition. (**A**) Volcano plot showing fold change of repetitive element subfamilies in siSMARCA4 versus siNT IMR90 ER:RAS + 4OHT cells. The dashed line indicates *P* adj. < 0.05. (**B**) Volcano plot showing fold change of satellite element loci in siSMARCA4 versus siNT IMR90 ER:RAS + 4OHT cells. Blue dots indicate significantly different (DESeq2, *P* adj. < 0.05, fold change > 1.5). (**C**) IF images and quantification of AU-15330–treated IMR90 ER:RAS cells positive for ORF1. Scale bar, 100 μm. Data represent means ± SEM (*n* = 4). (**D**) Representative IF images of cytoplasmic dsDNA staining (left), quantification of dsDNA intensity (center), and percentage of dsDNA-positive IMR90 ER:RAS cells (right) treated with and without AU-15330 as indicated. Data represent means ± SEM (*n* = 5). (**E**) IF images of IL-6 (top)– or IL-8 (bottom)–positive IMR90 ER:RAS cells treated with AU-15330 and lamivudine (3TC) as indicated. Scale bars, 100 μm. (**F**) Quantification of IL-6– or IL-8–positive cells from (E). Data represent means ± SEM (*n* = 3). (**G**) Representative IF images (left), quantification of dsRNA intensity (center), and percentage of dsRNA-positive IMR90 ER:RAS cells (right) treated with and without AU-15330 as indicated. Data represent means ± SEM (*n* = 5). (**H**) Schematic of siRNA experiment in AU-15330–treated IMR90 ER:RAS cells. (**I**) Quantification of IL-6–positive IMR90 ER:RAS cells. Data represent means ± SEM (*n* = 6). (**J**) Scheme showing SASP activation and the mechanism of NK cell recruitment following SMARCA4 inhibition in senescent cells. Ordinary one-way ANOVA (Tukey’s multiple comparisons test) was used for (C), ordinary one-way ANOVA (Dunnett’s multiple comparisons test) for (D) and (G), two-way ANOVA (Dunnett’s multiple comparisons test) for (F), and two-way ANOVA (Šídák’s multiple comparisons test) for (I). *P < 0.05, **P < 0.01, ***P < 0.001, and ****P < 0.0001.

We hypothesized that SMARCA4 inhibition could derepress and mobilize endogenous retrotransposable elements. To investigate that, we performed paired-end RNA-seq analysis of IMR90 ER:RAS senescent cells in which *SMARCA4* has been knocked down (fig. S10A). Principal components analysis of transposable element (TE) loci showed a separation between senescent and non-senescent cells upon *SMARCA4* knockdown (fig. S10B). Heatmaps showed that the expression of different TE subfamilies was induced during senescence. *SMARCA4* knockdown in senescent cells up-regulates a mostly different subset of TEs (fig. S10C). The top repeat subfamilies up-regulated upon *SMARCA4* knockdown in senescent cells included non-TE, satellite regions, *L1* (also termed *LINE1*) elements, and *MER92-int* (Fig. 6A). The expression of *MER92-int* has been recently linked with prognosis and NK response to colorectal carcinomas (*46*). While total repeat counts of all TE loci were mostly similar across conditions (fig. S10D), we observed trends toward increased expression of specific types of repetitive elements upon *SMARCA4* knockdown, including older *L1* elements, *SINE/Alu*, *MER92*, and satellites (fig. S10E). Volcano plots show a global derepression of satellite elements upon *SMARCA4* knockdown (Fig. 6B). Given that higher-order unfolding of satellite heterochromatin occurs on senescent cells (*47*), *SMARCA4* knockdown might derepress satellites.

To complement the above data, the activation of L1 expression in senescent cells was shown by IF against the L1 protein, ORF1p, as has been previously described (*45*). SMARCA4 inhibition further increased the expression of ORF1p (Fig. 6C). Moreover, the use of antibodies recognizing either single-stranded DNA (ssDNA; fig. S11A) or double-stranded DNA (dsDNA; Fig. 6D) detected increased levels of these nucleic acids in the cytoplasm, consistent with the potential mobilization and expression of TEs.

To understand whether TE-driven cytoplasmic DNA could contribute to explaining SASP induction upon SMARCA4 inhibition, we took advantage of three different reverse transcriptase (RT) inhibitors, 3TC, abacavir, and zidovudine. Analysis of the expression of IL-6 and IL-8 showed that the AU-15330 increase in SASP expression was prevented upon RT inhibition (Fig. 6, E and F, and fig. S11, B and C).

### Mechanism of SMARCA4-mediated SASP regulation

Because SMARCA4 inhibition seemed to affect broadly the expression of TEs (e.g., *SINE/Alu*), we speculated that innate immunity sensors other than cGAS/STING could be activated. We detected increased levels of cytoplasmic double-stranded RNA (dsRNA) upon SMARCA4 knockdown on senescent cells (Fig. 6G). Sensors such as retinoic acid-inducible gene I (RIG-I)and melanoma differentiation-associated protein 5 (MDA5) can detect cytoplasmic dsRNA to activate Mitochondrial antiviral-signaling protein (MAVS)-dependent inflammatory signaling (*48*).

We knocked down the expression of different components of these pathways (fig. S11D) to understand their role in regulating SASP expression in response to SMARCA4 inhibition (Fig. 6H). Knockdown of components of the cGAS/STING and the RIGI/MDA5/MAVS pathways partially affected the induction of IL-6 and IL-8 in response to SMARCA4 inhibition (Fig. 6I and fig. S11E). Moreover, the knockdown of TBK1 and the downstream transcription factors IRF3 and IRF7 also affected SASP expression (Fig. 6I and fig. S11E). GSEA analysis further confirmed the activation of dsRNA sensing pathways upon SMARCA4 knockdown in senescent cells (fig. S11F).

Because SMARCA4 is involved in chromatin remodeling and gene regulation, we wondered whether SMARCA4 directly controls the expression of any SASP genes. To address that question, we took advantage of a published study analyzing the effect of reexpressing SMARCA4 in small cell carcinoma of ovary, hypercalcemic type (SCCOHT) cells with deleted SMARCA4 (fig. S12A) (*49*). GSEA analysis of the RNA-seq data showed that SMARCA4 deletion results in increased SASP (fig. S12B) and IFN signaling (fig. S12C) but also increased senescence (fig. S12D). This was confirmed by the elevated expression of different SASP components and members of the cGAS/STING and RIG-I/MAVS sensing pathways (fig. S12E). In addition, Assay for Transposase-Accessible Chromatin using sequencing (ATAC-seq) and SMARCA4/BRG1 chromatin immunoprecipitation sequencing (ChIP-seq) in the same cells showed that genes encoding for SASP components, such as *IL6* or the innate immune sensors *STING1* and *RIGI*, are direct targets of SMARCA4, while others, such as *CXCL8*, might be indirect targets (fig. S12, F to I). Last, transcription factor motif enrichment analysis suggested that activator protein 1 (AP-1), which is known to control super-enhancers during senescence (*50*), is a master regulator of the process (fig. S12J). In summary, the above experiments suggest a mechanism by which SMARCA4 inhibition results in enhanced NK-mediated killing of senescent cells due to the derepression of TEs and other repeat elements activating innate immune sensing pathways (such as cGAS/STING and RIGI/MAVS), which results in increased SASP production (Fig. 6J).

### Analysis of SMARCA4 levels and NK infiltration in patients with ovarian cancer

**Fig. 7.**
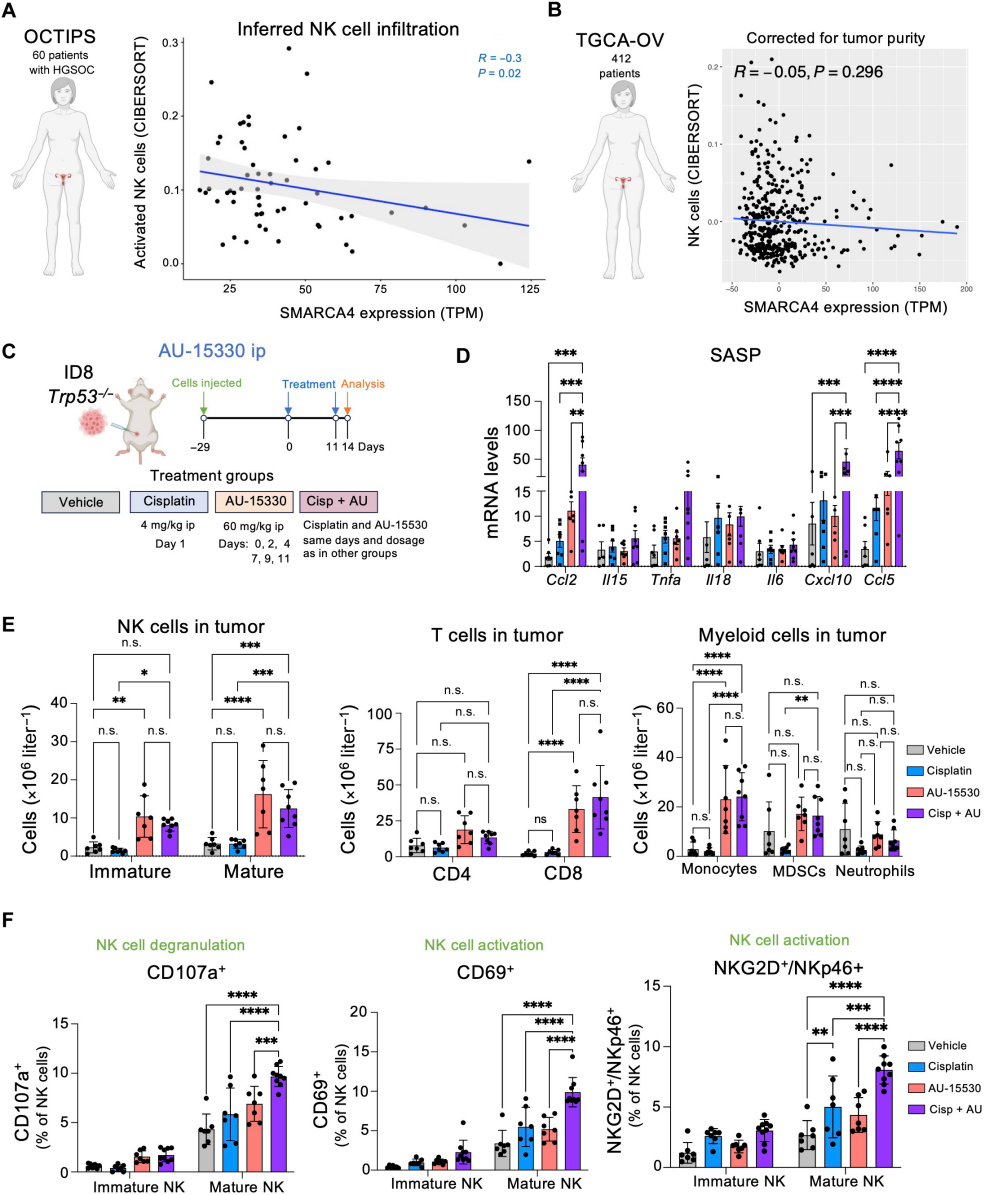
SMARCA4 inhibition increases NK cell infiltration and activation. (**A**) Sixty-six patients with HGSOC underwent primary debulking surgery, followed by RNA-seq of tumor samples derived from this cytoreduction. Six samples were excluded because of patients receiving neoadjuvant chemotherapy, resulting in a final cohort of 60 samples for downstream analysis. Spearman’s correlation between absolute NK cell infiltration as inferred by CIBERSORTx (*51*) and expression of SMARCA4 in transcript-per-million (TPM). *R*, Spearman *r*; *P*, *P* value. The linear regression line is in blue, with gray-shaded areas representing confidence intervals. (**B**) Spearman’s partial correlation between absolute NK cell infiltration as inferred by CIBERSORTx (*51*) and expression of *SMARCA4* in TPM in the TCGA-OV cohort, accounting for tumor purity (*n* = 412). (**C**) Schematic of the in vivo intraperitoneal injection of ID8 *Trp53*^−/−^ cells and treatment with cisplatin and AU-15330, alone or in combination. ip, intraperitoneal. (**D**) mRNA expression levels of markers for SASP in omental tumors measured by quantitative reverse transcription polymerase chain reaction (qRT-PCR). Data represent means ± SEM. **P < 0.01, ***P < 0.001, and ****P < 0.0001; two-way ANOVA (Tukey’s multiple comparisons test). (**E**) Flow cytometry analysis of NK, T, and myeloid cell counts in omental tumors. Data represent means ± SD. *P < 0.05, **P < 0.01, ***P < 0.001, and ****P < 0.0001; two-way ANOVA (Šídák’s multiple comparisons test). (**F**) Flow cytometry analysis of NK cell degranulation and activation in omental tumors. Data represent means ± SD. **P < 0.01, ***P < 0.001, and ****P < 0.0001; two-way ANOVA (Tukey’s multiple comparisons test). For (D) to (F), *n* = 7 mice for the vehicle, cisplatin, and AU-15330 groups; *n* = 8 for cisplatin in combination with AU-15330 (Cisp + AU).

To understand whether *SMARCA4* levels might (inversely) correlate with NK infiltration, we first analyzed the Ovarian Cancer Therapy Innovative Models Prolong Survival (OCTIPS) cohort of HGSOC (https://cordis.europa.eu/project/id/279113). We took advantage of RNA-seq data of samples obtained at diagnosis and applied CIBERSORTx (*51*) to infer NK cell infiltration. We observed an inverse correlation between inferred NK cells and SMARCA4 expression (Fig. 7A). Moreover, taking advantage of gene ontology analysis, we observed a similar inverse correlation between *SMARCA4* levels and signatures of NK cell activation, degranulation, and antitumoral responses (fig. S13, A to C). Furthermore, analysis of the The Cancer Genome Atlas Program-ovarian cancer (TGCA-OV) cohort showed that patients with mutated *SMARCA4* displayed a significantly higher inferred NK cell count (fig. S13D). Because inferring immune infiltration using bulk RNA-seq might be confounded for tumor purity, we further looked into the TGCA-OV cohort. Before correcting for tumor purity, we observed a (weaker in this cohort) significant inverse correlation between *SMARCA4* expression and inferred NK cell infiltration (fig. S13E), which disappeared after correcting for tumor purity (fig. S7B), suggesting caution in interpreting possible relations.

Analysis of the relationship between *SMARCA4* RNA expression and inferred NK infiltration in lung cancer taking advantage of the TRACERx cohort (*52*) showed a direct rather than inverse correlation (fig. S13F) that decreased when corrected for tumor purity (fig. S13, G to I). To further dissect the relationship between SMARCA4 and ovarian cancer, we evaluated single-cell SMARCA4 protein expression using tissue microarrays (TMAs) from a clinically annotated cohort High grade serous ovarian cancer-Centre hospitalier de l'Université de Montréal (HGSOC-CHUM) of treatment naïve patients. Using quantitative multiplex IF, HGSOC tissue cores were simultaneously stained for SMARCA4 and segmented into epithelium/stroma (cytokeratin positivity) and nuclear area [4′,6-diamidino-2-phenylindole (DAPI)] (fig. S14, A and B). There was a strong correlation between SMARCA4 expression in separate cores from the same patient (fig. S14C), validating the quantitative analysis. SMARCA4 protein expression was also higher in HGSOC epithelial cancer cells when compared to that in stromal cells using either nuclear mean fluorescence intensity (MFI) or SMARCA4-positive cell counts (fig. S14D). Using an unbiased Kaplan-Meier analysis of the cohort split 50/50 into patients with high or low SMARCA4 expression, we found associations between SMARCA4 high expression (in either the epithelial or the stroma compartment) and good clinical outcomes (fig. S14, E and F). This is consistent with similar observations made using bulk RNA expression from ovarian tumors in two different cohorts that were not specifically validated as HGSOC (*53*). Next, we performed immune cell quantification. Because CD56, a key NK marker in tissues, is expressed in ovarian cancer cells (*54*, *55*), we did not score NK infiltration but analyzed other immune populations. In general, macrophages (CD68^+^ cells) and T lymphocytes (CD3^+^ cells) counts per surface area were similar in the stromal and epithelial zones within each core (fig. S14, G to J). We could not observe a significant and consistent correlation between SMARCA4 in either compartment with either CD3^+^ or CD68^+^ cells in this cohort (fig. S14K). A deeper analysis of the immune microenvironment would be required to evaluate single-cell immune activation, including that of NK cells in relationship to SMARCA4 expression (*54*). Overall, although connections between SMARCA4, immune regulation, and clinical outcomes exist, the data suggest that these relations are complex and probably context dependent.

### Immunomodulatory effects of SMARCA4 inhibition in ovarian cancer

To investigate the therapeutic potential and immunomodulatory effects associated with SMARCA4 inhibition, we took advantage of the previously described ID8 *Trp53*^−/−^ ovarian cancer model. We injected ID8 *Trp53*^−/−^ cells intraperitoneally and, once tumors became established, divided mice into four cohorts and examined the tumors 14 days after treatment (Fig. 7C). All treatments were well tolerated, and we did not observe any notable toxicities or alterations in the spleen and only a transient weight loss after cisplatin treatment (fig. S15, A and B). While intraperitoneal administration of AU-15330 alone did not decrease tumor size, treatment with cisplatin resulted in a reduction in mean tumor size (fig. S15C). The combination of cisplatin and AU-15330 resulted in a significant reduction in mean tumor size (fig. S15C). A higher percentage of the tumors of mice treated with the combination of cisplatin and AU-15330 were smaller in size when compared with those in the vehicle or the cisplatin-treated group (fig. S15D).

To investigate the possible immunomodulatory effects of the combination treatment, we analyzed by quantitative reverse transcription polymerase chain reaction (qRT-PCR) the expression of different cytokines and chemokines that form part of the SASP. We observed induction of most of them in the combination group; in particular, we observed significant up-regulation of transcripts encoding for immunomodulatory chemokines including *Ccl2*, *Cxcl10*, and *Ccl5* (Fig. 7D).

Fluorescence-activated cell sorting (FACS) analysis of immune infiltration detected a significant increase in mature (NK1.1^+^ and CD49b^+^) NK cells, CD8^+^ T cells, and monocytes in mice treated with AU-15330 either alone or in combination with cisplatin (Fig. 7E). The expression of transcripts encoding for NK-activating ligands including *Rae1a Rae1c* and *Grzb* (fig. S15, E and F), the transcript encoding for granzyme B, indicative of immune cell activation, was also up-regulated. *Grzb* is also expressed in cytotoxic T cells, and our experiment cannot assess the relative contribution of NK and T cells. However, we characterized further the tumor-infiltrated NK cells, and we observed an increase of cytotoxic mature NK cells, as shown by the degranulation marker CD107a and NK cell activation markers (CD69^+^ and NKG2D^+^/NKp46^+^) in mice treated with AU-15330 and cisplatin (Fig. 7F). Furthermore, we observed also an increase in IFN-γ secretion by mature NK cells in mice treated with AU-15330 in combination with cisplatin (fig. S15G). No significant differences were observed in the infiltrated B cells, macrophages, or dendritic cells (fig. S15H). Overall, the above results show that treatment with AU-15330 in combination with cisplatin enhanced the infiltration of CD8 T and activated, mature NK cells in the tumor.

### Effect of combined cisplatin/AU-15-330 treatment in ovarian cancer

**Fig. 8.**
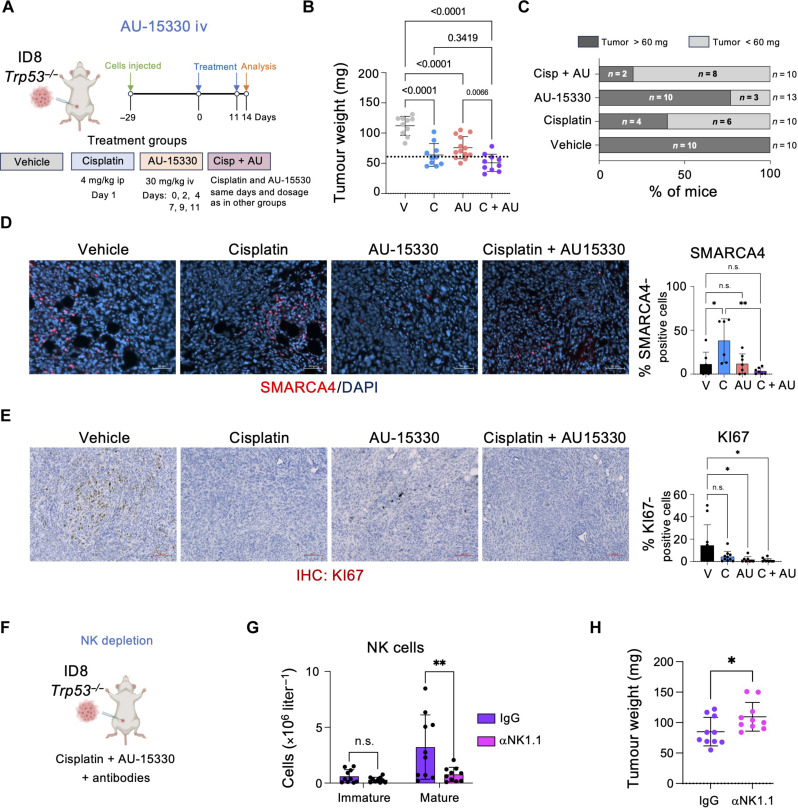
Treatment with AU-15330 in combination with cisplatin reduces ovarian tumor size in an NK-dependent manner. (**A**) Schematic of the in vivo intraperitoneal injection of ID8 *Trp53*^−/−^ cells and treatment with cisplatin and AU-15330, alone or in combination. iv, intravenous. (**B**) Tumor weight (milligrams) of mice treated with vehicle (V), cisplatin (C), AU-15330 (AU), and cisplatin in combination with AU-15330 (C + AU). Data represent means ± SD; one-way ANOVA (Tukey’s multiple comparisons test). (**C**) Percentage of mice with a tumor weight below 60 mg from (B). For (B) and (C), *n* = 10 mice for the V, C, and C + AU groups; *n* = 13 mice for the AU group. (**D**) Representative images (left) and quantification (right) of SMARCA4 IF staining in omental tumors. Scale bar, 50 μm. Data represent means ± SD. *P < 0.05 and **P < 0.01; one-way ANOVA (Tukey’s multiple comparisons test). *n* = 7 mice for the V, AU, and C + AU groups; *n* = 6 mice for the C group. (**E**) Representative images (left) and quantification (right) of Ki67 staining in omental tumors. Scale bars, 100 μm. Data represent means ± SD. *P < 0.05; one-way ANOVA (Tukey’s multiple comparisons test). *n* = 10 mice for the V, C, and C + AU groups; *n* = 8 mice for the AU group. (**F**) Schematic of the in vivo depletion of NK cells in ID8 *Trp53*^−/−^ cells injected intraperitoneally. Mice were treated with the combination of cisplatin and AU-15330 together with αNK1.1 antibody. Immunoglobulin G (IgG) antibody was used as a control. (**G**) Flow cytometry analysis of NK cell counts in omental tumors. Data represent means ± SD. **P < 0.01; two-way ANOVA (Šídák’s multiple comparisons test). (**H**) Tumor weight (milligrams) of mice treated with vehicle C + AU + αNK1.1 and C + AU + IgG. Data represent means ± SD; Mann-Whitney test. For (F) to (H), *n* = 10 mice for the C + AU + IgG and C + AU + αNK1.1 groups.

While the above-described experiments show that SMARCA4 inhibition results in increased NK infiltration in a murine model of ovarian cancer, we observed increased ascites formation and no reduction in tumor size upon intraperitoneal injection with the SMARCA4 PROTAC AU-15330. We modified the treatment protocols to administer AU-15330 intravenously, similar to what has been used to treat other cancer models (Fig. 8A) (*38*). All treatments were well tolerated, and we did not observe any toxicities or alteration in the spleen (fig. S16, A and B). Intravenous treatment with AU-15330 did not increase the ascites formation but resulted in a significant decrease in tumor size, in contrast with what we observed with intraperitoneal AU-15330 administration (Fig. 8B). Analysis of mean tumor weight showed trends to a decrease in size when comparing the combined treatment with individual treatments (Fig. 8B). Moreover, analysis of the individual tumors showed a decrease in the bigger tumors (size > 60 mg) upon combination treatment (20%), when compared with cisplatin treatment (40%) or AU-15330 (76%) (Fig. 8C). Quantitative IF confirmed an increase of SMARCA4 upon cisplatin treatment that was not observed in the combined treatment, consistent with AU-15330 degrading SMARCA4 (Fig. 8D). Moreover, we observed a significant decrease in proliferating cells in the tumors treated with the combination, as assessed by KI67 staining (Fig. 8E). We confirmed similar changes in NK cell infiltration and activation in tumors from mice treated with cisplatin and intravenous AU-15330, as we have seen before (fig. S8, C to F).

Next, we used antibodies against NK1.1 that cause NK depletion to investigate the contribution of NK cells to the antitumoral responses observed in mice treated with AU-15330 and cisplatin (Fig. 8F). Analysis of the tumor-infiltrated populations confirmed the specific depletion of NK cells in mice treated with αNK1.1 antibodies (Fig. 8G). There was no impact in other immune populations such as T cells, B cells, myeloid cells, or macrophages (fig. S16, G to J). Mice treated with cisplatin in combination with AU-15330 with NK cell depletion showed a significant increase in the mean tumor growth, when compared to the immunoglobulin G (IgG)–treated mice (Fig. 8H), which was associated with decreased apoptosis in the tumors (fig. S16K). Overall, the above results suggest that SMARCA4 inhibition could be combined with existing therapies to control ovarian cancer and NK cells play an important role in explaining the observed antitumoral effect.

## DISCUSSION

The accumulation of senescent cells contributes to many age-related diseases, including cancer. Consequently, their selective elimination positively affects life span and health span (*56*). Despite overwhelming evidence for the benefits associated with eliminating senescent cells, the most studied senolytic agents, such as navitoclax or the combination D + Q, have important side effects, reduced selectivity, or unclear mechanisms of action. Immune-mediated surveillance is the natural mechanism controlling the elimination of senescent cells. Therefore, ways to harness the immune system to remove lingering senescent cells are an attractive alternative to the use of senolytic drugs. In that regard, recent work has shown how therapeutic combinations such as P + T can induce senescence and trigger an NK-mediated response that improves outcomes in preclinical models of lung cancer (*11*). Expression of chemoattractants as part of the SASP is a key factor regulating senescence surveillance. For example, EZH2 inhibitors can enhance the production of SASP chemokines and potentiate NK and T cell–mediated immune responses in models of pancreatic cancer (*23*). A dual G9A/EZH2 inhibitor also stimulates antitumor immune responses in a model of HGSOC (*57*).

Given the important role of NK cells in the clearance of senescent cells (*58*), we investigated ways to potentiate NK-mediated elimination of senescent cells. To this end, we used a staggered screening approach. First, we searched for siRNAs that augmented SASP production. Consequently, we shortlisted genes whose knockdown resulted in SASP superinduction and tested whether siRNAs targeting these genes potentiated NK-mediated killing of senescent cells. Our screen identified 10 genes whose knockdown enhanced the NK-mediated killing of cells undergoing OIS.

We chose to validate 5 of those 10 hits: *SMARCA4*, *FOSL1*, *POLR1B*, *FURIN*, and *CD247*. We confirmed the effects of NK-mediated killing of senescent cells upon knockdown of the five hits. Ablating these genes selectively enhanced the NK-mediated killing of senescent but not normal cells. The reduction in senescent cell numbers depended on the coculture with NK cells, therefore discarding senolysis as the main mechanism explaining these results.

Among the screen hits, SMARCA4 was particularly attractive. Taking advantage of a SMARCA4 targeting PROTAC (*38*), we confirmed that SMARCA4 inhibition increased the SASP and NK-mediated killing of different types of senescent cells. While in those settings we did not observe that SMARCA4 inhibition induced or exacerbated senescence, we cannot discard that it does so in some scenarios, as observed in SCCOHT cells with *SMARCA4* deletions (*49*). We used an immortalized NK cell line (NK92-MI) for most of our coculture experiments, but primary human NK cells (derived from either peripheral or cord blood) also killed more efficiently senescent cells treated with SMARCA4 inhibitors.

SMARCA4 is a component of the SWI/SNF chromatin remodeling complex, and it is often mutated in cancer (*59*). Evidence in the literature points to a role for SMARCA4 and the SWI/SNF complex in modulating immune responses in cancer. For example, while alterations in SWI/SNF genes are not frequent in pancreatic cancer, patients displaying these alterations are more responsive to ICIs (*60*). A study in head and neck cancer found deletions of SMARCA4 as one of the most common occurrences in patients responding to ICI (*61*), and there have been also reports of complete remission in a patient with stage IV *SMARCB1*-inactivated epithelioid sarcoma (*62*).

We observed that SMARCA4 (and other SWI/SNF components) are up-regulated during senescence. While trying to understand how SMARCA4 inhibition increased SASP production, we observed a hyperactivation of the cGAS/STING pathway in senescent cells treated with SMARCA4 inhibitors. cGAS/STING is a key sensor of cytoplasmic DNA playing an important role in SASP activation during senescence (*43*, *44*). Chromatin cytoplasmic foci (*43*), mitochondrial DNA, and the mobilization of endogenous retrotransposable elements (*45*) have all been shown to contribute to cGAS activation during senescence. We also observed elevated levels of cytoplasmic dsRNA, which can be sensed by RIG-I to induce inflammatory factors during senescence (*63*). We observed the induction of different repetitive elements, including *L1* elements and satellites in senescent cells treated with SMARCA4 inhibitors. Therefore, derepression of TEs caused by SMARCA4 inhibition might be behind the activation of innate immune sensing pathways and increased SASP levels. However, we cannot discard that other mechanisms also contribute to the up-regulation of the SASP in response to SMARCA4 inhibition in senescent cells.

*TP53* mutations are near-universal in HGSOC. To assess the therapeutic potential of SMARCA4 inhibition, we took advantage of a preclinical model in which ID8 *Trp53*^−/−^ cells were injected intraperitoneally in syngeneic mice. While cisplatin treatment alone significantly reduced tumor size, combination with the SMARCA4 targeting PROTAC AU-15330 resulted in a higher percentage of mice with reduced tumor size, reduced tumor proliferation, and increased immune infiltration. We observed increased infiltration of both CD8^+^ T cells and mature, activated NK cells intratumorally. Increased immune infiltration was also observed in mice treated only with AU-15330, suggesting that SMARCA4 inhibition could also increase immune infiltration in the absence of senescence. Because SMARCA4 inhibition up-regulates the expression of chemoattractants as part of the SASP, it is expected that the enhanced immune response is not limited to NK activation. Nevertheless, antibody depletion experiments confirmed the important role of NK cells in the antitumoral response. The genetic landscape of HGSOC is complex but relatively stable between diagnosis and relapse (*64*). Consequently, epigenetic modulators such as DNA methyltransferase (DNMT) inhibitors (*65*) or EZH2/G9a dual inhibitors (*57*) have been suggested as potential therapies. In addition, carboplatin-based first-line therapies and poly(adenosine 5′-diphosphate–ribose) polymerase (PARP) inhibitors induce senescence in HGSOC cells (*28*), enabling SMARCA4 inhibition as a potential therapy for HGSOC in multiple contexts. Nevertheless, while analysis of different HGSOC patient cohorts suggests that SMARCA4 levels might relate to NK and immune cell infiltration, we should be cautious in overinterpreting a direct relation between SMARCA4 and NK in patient-derived samples.

In summary, our study identifies ways to potentiate the NK-mediated responses against senescent cells. Taking advantage of preclinical models of HGSOC, we provide evidence of the therapeutic potential of SMARCA4 inhibition as an anticancer therapy. Moreover, given the key roles that NK cells play in the surveillance and elimination of senescent cells in fibrosis and aging, the potential for SMARCA4 inhibition could be extended to a wide range of senescence-associated diseases.

## MATERIALS AND METHODS

### Ethics

This research complied with all relevant ethical regulations and was approved and overseen by the following ethics review boards. The OCTIPS cohort (described at https://cordis.europa.eu/project/id/279113) includes samples from 66 patients with HGSOC who underwent primary debulking surgery (all are females, between the ages of 21 and 74 at diagnosis, median of 55.5 years). Informed consent was obtained from each patient before sample collection and approved by the relevant ethical committee for each locality. RNA-seq was performed on tumor samples, with the exclusion of six samples from patients who had received neoadjuvant chemotherapy, resulting in a final cohort of 60 samples. Whole blood was collected from the umbilical cord under the Imperial College Healthcare Tissue Bank (ICHTB) Human Tissue Act (HTA) license 12275 and with Research Ethics Committee approval: 17/WA/0161. Whole blood was isolated from leukocyte cones purchased from the National Health Service (NHS) Blood and Transplant Service.

The HGSOC-CHUM cohort was constituted following approval from the institutional ethics committee. Informed consent from all 76 patients was obtained before sample collection, and samples were anonymized. Tumor samples were collected from patients who underwent surgery at the CHUM Department of Gynecologic Oncology for ovarian cancer between 1993 and 2014. The disease stage was validated by a pathologist and defined using the International Federation of Gynecology and Obstetrics staging system. Tumor histopathology was classified using the World Health Organization criteria. Patient survival was calculated from the date of diagnosis, and 5-year overall survival was defined as the time from diagnosis to death or last follow-up, up to a maximum follow-up of 5 years. For TMA construction, two cores of 0.6-mm diameter for each tissue sample were arrayed onto recipient paraffin blocks.

Experiments performed in mice were approved by the Animal Welfare and Ethics Review Body at Imperial College London. All experiments conformed to UK Home Office regulations under the Animals (Scientific Procedures) Act 1986, including Amendment Regulations 2012 and adhered to the Animal Research: Reporting of In Vivo Experiments (ARRIVE) guidelines. Liver cancer initiation experiments were performed under project license number PPL 70/09080. Ovarian cancer experiments were performed under the project license numbers PA780D61A and PP1321516.

### Drugs

The compounds used in this study are summarized in table S4.

### Antibodies

The primary antibodies used for IF are in table S5, and the ones used for FACS are in table S6. The secondary antibodies used in this study are in table S7.

### Cell lines

IMR90 [human; ATCC, CCL-186], OVCAR4 (human; National Cancer Institute, Frederick, MA), and human embryonic kidney (HEK)–293T (human; ATCC, CRL-11268) cells were obtained from the American Type Culture Collection (ATCC). ID8 *Trp53*^−/−^ ovarian cancer cells (derived from C57BL/6 mouse) have been described before (*40*). IMR90 ER:RAS cells were generated by retroviral infection of IMR90 cells and have been described elsewhere (*32*). For IncuCyte experiments, IMR90 ER:RAS H2B-GFP cells were generated through stable lentiviral infection of IMR90 ER:RAS cells with the FU-H2B-GFP-IRES-Puro vector (Addgene, no. 69550) for constitutive expression of a nuclear green fluorescent protein (GFP) signal. Infected IMR90 ER:RAS cells were selected by treatment with puromycin (1 μg/ml; InvivoGen, catalog no. ant-pr-1) and maintained at a working concentration of 0.5 μg/ml. For coculture experiments, the NK92-MI lymphoma NK cell line (ATCC, CRL-2408) was a gift from V. Krizhanovsky (Weizmann Institute of Science, Rehovot, Israel).

IMR90, HEK-293T, IMR90 ER:RAS, and OVCAR4 were cultured in Dulbecco’s modified Eagle’s medium (DMEM; Gibco, catalog no. 41965120) supplemented with 10% fetal bovine serum (FBS; Sigma-Aldrich, catalog no. F7524) and 1% antibiotic-antimycotic solution (Gibco, catalog no. 15240062), hereafter referred to as “D10 medium.” ID8 *Trp53*^−/−^ were cultured in DMEM (high glucose, pyruvate; Gibco, catalog no. 11995-073) with 4% FBS (Sigma-Aldrich, catalog no. F7524), 1% antibiotic-antimycotic solution (Gibco, catalog no. 15240-062), and 1% insulin-transferrin-selenium (Gibco, catalog no. 2506891).

NK92-MI cells were maintained in minimum essential medium–α with no nucleosides (Gibco, 22561054), supplemented with 12.5% FBS, 12.5% FBS horse serum (Gibco, 16050130), 1% 100× antibiotic-antimycotic solution, 0.6 ml of inositol (Sigma-Aldrich, I7508), and 0.2 M filter-sterilized and 0.6 ml of folic acid (Sigma-Aldrich, F8758), prepared in 0.1 M NaHCO_3_ and 0.02 M filter-sterilized and 4.1 μl of β-mercaptoethanol (Gibco, 21985023).

The purification and culture of cord blood– and peripheral blood–derived NK cells are reported in detail below (see the “Isolation of mononuclear cells and expansion of NK cells” section). To induce OIS, IMR90 ER:RAS cells were treated with 100 nM 4OHT (Sigma-Aldrich, 68047-06-3) reconstituted in dimethyl sulfoxide (DMSO; Sigma-Aldrich, D2650). For chemotherapy-induced senescence, IMR90 cells were treated with 50 μM etoposide (Tocris, 1226) for 48 hours. In OVCAR4 and ID8 *Trp53*^−/−^ cell lines, senescence was induced by cisplatin treatment for 6 days. In OVCAR4, the concentration of cisplatin used was 1.25 and 2.5 μM, while, in ID8 *Trp53*^−/−^, the concentration of cisplatin used was 1 μM.

### Isolation of MNCs and expansion of NK cells

Whole blood was collected from the umbilical cord under the ICHTB HTA license 12275 and with REC approval: 17/WA/0161. Whole blood was isolated from leukocyte cones purchased from the NHS Blood and Transplant Service. Mononuclear cells (MNCs) were isolated from whole blood by Ficoll-Paque PLUS (Cytiva) separation. MNCs were cryopreserved in 90% heat-inactivated FBS (HIFBS) (Thermo Fisher Scientific) and 10% DMSO at a cell concentration between 1 × 10^7^ and 1 × 10^8^ per milliliter per vial. Before expansion, MNCs were thawed and allowed to recover overnight in RPMI 1640 (Sigma-Aldrich) supplemented with 10% HIFBS, 100× non-essential amino acids (Sigma-Aldrich), 100× Hepes (Sigma-Aldrich), 1000× 2-mercaptoethanol (Gibco), 100 IU of recombinant human IL-2 (Peprotech), and recombinant human IL-15 (10 ng/ml; Peprotech) at 37°C and 5% CO_2_. MNCs were depleted of CD3^+^ cells using Miltenyi QuadroMACS Separator with phycoerythrin (PE) microbeads (Miltenyi) following the supplier’s instruction. Briefly, MNCs were stained with anti-human CD3 PE, clone 4H11 (BioLegend) at 1:100 concentration per 1 × 10^7^ cells for 15 min at 4°C. Cells were then stained with PE Microbeads at 10 μl per 1 × 10^7^ cells for 15 min at 4°C. Stained cells were loaded onto LD columns (Miltenyi). Unbound CD3-negative cells were then expanded in RPMI 1640 supplemented with 10% HIFBS and 200 IU of IL-2. Cells were cocultured with sublethally irradiated K562 feeder cells expressing membrane-bound IL-21 (mbIL-21), at a 10:1 (feeder:MNC) ratio. The culture was restimulated with irradiated mbIL-21 K562 cells at expansion day 7 at a 5:1 ratio. At expansion day 14, before use in functional assays, the culture was enriched for CD56^+^ cells using Miltenyi QuadroMACS Separator with PE microbeads following the supplier’s instruction. Briefly, cells were stained with anti-human CD56 PE antibody (Invitrogen) at 1:100 per 1 × 10^7^ cells for 15 min at 4°C. Cells were washed with phosphate-buffered saline (PBS) and then stained with PE Microbeads at 10 ml per 1 × 10^7^ cells for 15 min at 4°C. Cells were washed and then loaded onto an LS Column that was then washed with PBS. The LS Column was removed from the magnetic block, and bound CD56 cells were purged in 5 ml of FBS.

### Growth assays

For 5-bromo-2′-deoxyuridine (BrdU) incorporation assays, the cells were incubated with 10 μM BrdU (Sigma-Aldrich, 858811) for 16 to 18 hours and then fixed with 4% paraformaldehyde (PFA; Thermo Fisher Scientific, 033314.M1). BrdU incorporation was assessed by IF and high-content analysis microscopy. For crystal violet staining, the cells were seeded at a low density on six-well dishes and fixed at the end of the treatment with 0.5% glutaraldehyde. The plates were then stained with 0.2% crystal violet.

### IF staining of cells

Cells were grown in 96-well plates (Nunc MicroWell, 167008) and cultured, as required. At the desired time point, cells were fixed with 4% PFA for 45 min, permeabilized in 0.2% Triton X-100 (Sigma-Aldrich, 11332481001), diluted in PBS for 10 min, and blocked with 1% bovine serum albumin (Sigma-Aldrich, catalog no. A2153) and 0.2% fish gelatin (Sigma-Aldrich, catalog no. G7765) for 1 hour at room temperature. Cells were then incubated with a primary antibody for 1 to 2 hours at room temperature, followed by the corresponding fluorescence-labeled secondary antibody (Alexa Fluor) for 45 min and DAPI (1 μg/ml; w/v, PBS) for 15 min at room temperature. For BrdU staining, primary BrdU antibody was added together with deoxyribonuclease (DNAse; 0.5 U/μl) and 1 mM MgCl_2_ in blocking solution and incubated for 30 min at room temperature. Primary and secondary antibodies used are listed in tables S5 and S7, respectively. Antibodies were diluted in a blocking solution. After every step, cells were washed with PBS three times.

### High-throughput microscopy and quantitative analysis

Images of wells from 96-well plates were acquired using the IN Cell Analyzer 2500HS high-throughput microscope and plate reader (Cytiva). For images of only DAPI (cell count), images of wells were captured at a 10× objective. For all IF staining, wells were imaged with a 20× objective except BrdU staining (imaged using a 10× objective) and DNA damage foci, dsDNA, ssDNA, and dsRNA analysis (imaged using a 40× objective). Fluorescent images of fluorophores were captured on the basis of the pre-set wavelength settings for “DAPI” (for DAPI staining), “Red” (for Alexa Fluor 594), and “Green” (for Alexa Fluor 488) on the IN Cell microscope. High content analysis of acquired images was performed using the IN Cell Investigator 2.7.3 software (GE Healthcare). DAPI nuclear staining was used as a nuclear mask to quantify cells and allow the segmentation of cells based on a Top-Hat method, according to the software guidelines. This method also allowed the detection of nuclear-localized antibody staining. Nuclear staining was quantified by the average pixel intensity within the nuclear mask. Cytoplasmic staining was quantified after applying a collar of 6 to 9 μm around the DAPI mask. Cytoplasmic-localized staining was quantified either by the average pixel intensity or by measuring the coefficient of variance (CV) pixel intensity within the cell collar. Detection of percentage positive cells was based on a threshold, typically set according to the average pixel intensity of an internal negative control or an unstained secondary antibody–only control sample, unless specified. For IF staining of ssDNA, dsDNA, and dsRNA, cytoplasmic-localized staining was quantified by measuring the CV pixel intensity within the cell collar excluding the nucleus.

### Cytochemical SA-β-Gal assay

Cells were grown on six-well plates, fixed with 0.5% glutaraldehyde (Sigma-Aldrich, G5882) in PBS for 15 min, washed with 1 mM MgCl_2_/PBS (pH 6.0), and then incubated with X-Gal staining solution {X-Gal (1 mg ml^−1^; Thermo Fisher Scientific, 2737087), 5 mM K_3_[Fe(CN)_6_], and 5 mM K_4_[Fe(CN)_6_]} for 6 to 8 hours at 37°C. Bright-field images of cells were taken using the DP20 digital camera attached to the Olympus CKX41 inverted light microscope. The percentage of Sa-β-Gal–positive cells was estimated by counting at least 100 cells per replicate sample facilitated by the “point picker” tool of ImageJ software (National Institutes of Health).

### RNA isolation and purification

For total RNA isolation, cells were seeded in 6-cm dishes. Cells were dissociated using trypsin and washed with PBS and lysate. Total RNA from the cell pellet was then isolated and purified using the RNAeasy Mini Kit (QIAGEN, 74106), according to the manufacturer’s protocol. The total RNA isolated was eluted in 30 to 50 μl of ribonuclease-free water, and RNA concentration was quantified using the NanoDrop ND-1000 ultraviolet-visible spectrophotometer. RNA was stored at −80°C for long-term preservation.

### RNA isolation and purification from omental tumor tissues

Omental tumors were homogenized mechanically with pestles (Corning, PES-15-B-SI) in 500 μl of TRIzol (Ambion, Life Technologies, 15596026). Next, 100 μl of chloroform (Sigma-Aldrich, 319988) was added, and the samples were centrifuged at 12,000 rpm for 30 min. The transparent phase was collected and mixed with an equal volume of ethanol (70%). The samples were then moved to the RNAeasy Mini Kit column, and the RNA was extracted according to the manufacturer’s protocol.

### cDNA generation and qRT-PCR

The cDNA was generated using random hexamers and SuperScript II RT (Invitrogen, 18064-014), according to the manufacturer’s protocol. qRT-PCR was performed using SYBR Green PCR master mix (Applied Biosystems) in a CFX96 RT-PCR detection system (Bio-Rad). The primers used for qRT-PCR are listed in tables S8 and S9. The delta-delta threshold cycle (C_T_) method (2^−∆∆Ct^) was applied to the resulting CT values obtained during the amplification phase to calculate relative mRNA expression levels. *RPS14* (human), *Gapdh* (mouse cells), and *Rn18S* (mouse tissues) were used as the housekeeping control for normalization, and an untreated sample was used as an internal relative control (DMSO). The 2^−∆∆Ct^ method calculation is as follows

$$\Delta \Delta \text{Ct}={\Delta \text{Ct}}_{\text{treated}}-{\Delta \text{Ct}}_{\text{control}}/\Delta \text{Ct}={\text{Ct}}_{\text{target}}-{\text{Ct}}_{\text{housekeeping}}$$

### Generation of CM

IMR90 ER:RAS (0.3 × 10^5^ or 1.0 × 10^5^) cells were seeded in six-well plates for the DMSO and 4OHT conditions, respectively. The following day, DMSO and 4OHT treatments were administered as appropriate. Any compounds used were administered 4 days after DMSO/4OHT treatment. After 7 days of DMSO/4OHT treatment, the D10 medium was replaced with serum-free D10 medium and maintained in culture for 48 to 72 hours to generate conditioned medium (CM). After this time point, CM was collected, centrifuged to remove cellular debris, and aliquoted to be stored at −80°C.

### Enzyme-linked immunosorbent assay

To detect cytokines secreted in the CM, enzyme-linked immunosorbent assays were performed using kits for IL-6 (R&D Systems, DY206), IL-8 (R&D Systems, DY208), and CXCL10 (R&D Systems, DY266), according to the manufacturer’s guidelines. For absorbance measurements, readings were taken using a SPECTRAmax 340PC Microplate Spectrophotometer (Molecular Devices) at 450 nm.

### Live cell imaging assay to assess NK-mediated killing of senescent cells

IMR90 ER:RAS H2B-GFP cells were seeded in 96-well plates and allowed to adhere overnight (day −1). Additional replicate plates were also seeded, labeled as “time 0.” The following day (day 0), cells were treated with DMSO or 4OHT until the desired time point (7 to 8 days, depending on the experiment). Medium was replaced every 3 to 4 days. On the day of coculture, time 0 plates were fixed with 4% PFA (w/v, PBS) for 20 min, followed by 10-min incubation with DAPI solution (1 μg/ml; w/v, PBS). Cell counts from time 0 plates were quantified on the basis of the number of stained nuclei to determine the correct E:T ratio of NK cells required, using the IN Cell Analyzer 2500HS. NK92-MI cells were stained red with 1 μM CellTracker Red CMTPX Dye (Invitrogen, C34552) for 30 to 45 min, according to the manufacturer’s instructions. NK cells were then added to the 96-well plates containing IMR90 ER:RAS cells at the required E:T ratios in triplicates. Additional triplicate control wells received only medium alone without NK cells. For the duration of the coculture, cells were maintained in regular D10 growth medium unless otherwise stated. Cocultures were imaged every 2 hours using a 10× objective up to the end time point (usually 48 hours) using the IncuCyte Live-Cell Analysis Systems (Sartorius). NK cell–mediated cytotoxicity was assessed by the percentage change in IMR90 ER:RAS H2B-GFP green object count during the coculture period using the built-in IncuCyte Zoom software (Essen Bioscience).

### High-throughput imaging and analysis of NK-mediated killing of senescent cells

NK cell–mediated killing was measured using the IN Cell Analyzer 2500HS high-throughput microscope and plate reader (Cytiva). The assay was performed similarly to that described above, with a few adjustments. Fluorescent reporter expression in cells was not required. IMR90, IMR90 ER:RAS, or OVCAR4 cells were seeded in 96-well plates and allowed to adhere overnight (day −1). Time 0 plates were also seeded. The following day (day 0), cells were treated with the appropriate senescence inducer until the required time point (~7 to 8 days). Medium was replaced every 3 to 4 days. Any drugs tested to promote or inhibit NK-mediated cytotoxicity were added on day 4.

On the day of coculture, cell numbers from the time 0 plates were quantified as described above. NK92-MI cells were then starved in plain DMEM lacking FBS for 30 to 45 min before being added to the 96-well plates containing cells in triplicates at the required E:T ratios. Additional triplicate wells received only medium alone without NK cells as a control. For the duration of the coculture, cells were maintained in regular growth medium in the absence of drugs unless otherwise stated. Plates were incubated at 37^o^ C for the desired time (typically 48 hours, 24 hours for OVCAR4 cells). Plates were then fixed using 4% PFA (w/v, PBS) for 20 min, followed by 10-min incubation with DAPI solution (1 μg/ml; w/v, PBS) to label the nuclei. Following the coculture, all plates were fixed, stained with DAPI, and imaged using the IN Cell Analyzer 2500HS (Cytiva). NK cell–mediated cytotoxicity was assessed by the percentage change in DAPI-positive cell counts during the coculture period using the InCell Investigator 2.7.3 software.

### Transfection of siRNAs and SASP screen

Druggable genome siRNA libraries were purchased from Horizon Discovery (human siGENOME SMARTpool). Custom-designed siRNA library plates and individual siRNAs were purchased from the siGENOME siRNA reagent human catalog from Dharmacon (Horizon Discovery), and all plates arrived lyophilized coated onto 96-well plates (0.1 nM) or in a tube (for individual siRNA orders). The siRNA 96-well plates (0.1 nM) were reconstituted in 100 μl of nuclease-free water and aliquoted appropriately in round bottom 96-well (Nunc, 163320) daughter plates for storage at −20°C. For siRNA transfection, 3.6 μl of aliquoted siRNA was added to flat-bottom 96-well plates (Nunc MicroWell, 167008). A “transfection mix” was generated comprising 17.5 μl of plain DMEM (Gibco) and 0.1 μl of DharmaFECT1 transfection reagent per well (Horizon Discovery, T-2005-01). The transfection mix (17.6 μl) was added to each well already containing 3.6 μl of siRNA (or for mock wells, containing 3.6 μl of water), and the mixture was incubated at room temperature for 30 min. For reverse transfection, IMR90 ER:RAS cells were first quantified, and 100 μl of cell suspension (in D10 medium supplemented with only 10% FBS but no antibiotics) was added to the 96-well plates (containing siRNA and transfection mix) at the appropriate seeding density. This resulted in a final siRNA concentration of 30 nM, and the plates were incubated at 37°C for 18 hours to allow cells to adhere and be reverse transfected (day −1). Following this period, the transfection medium was discarded and replaced with fresh D10 medium supplemented with the presence of DMSO or 4OHT as appropriate (day 0). Four days following the initial DMSO/4OHT treatment (day 4), the medium was replaced again. Seven days after the initial DMSO/4OHT treatment (day 7), cells were fixed in 4% PFA (w/v) for 45 min (for IF), washed, and stored in PBS at 4°C. For assessing levels of SASP components, IF was performed as described. Sequences for siRNA candidates tested are listed in table S10.

### Normalization of the screens

For the primary siRNA screens, *B*-score normalization was performed using the CellHTS2 package on R (*66*). All data were assigned a normalized *B* score using the plate-averaging method based on separate plate batches of transfection. A threshold was set to determine the hits.

For the secondary siRNA screens, the NPA method was used. Each siRNA was assigned an NPA score on the basis of the following calculation: NPA = (*X_i_* − C ®neg)/(C ®pos − C ®neg), where *X_i_* is the percentage positive value of SASP for the sample measurement on the *i*th siRNA, and C ®neg and C ®pos are the means of the percentage positive SASP values of the negative and positive controls, respectively, in the SASP superinducer screen.

### siRNA transfection for the screen for NK-mediated killing of senescent cells

siRNA transfection for the NK-mediated killing screen followed an identical protocol to that of the siRNA SASP superinducer screen with a few adjustments. Seven days following the initial DMSO/4OHT treatment day 7, NK cells were cocultured with growing and senescent siRNA-transfected cells for 48 hours. Following this period, all plates were fixed with 4% PFA (w/v) for 20 min and stained with DAPI for 10 min before being imaged using the IN Cell Analyzer 2500HS workstation. Cell quantification was performed using the IN Cell Investigator 2.7.3 software. Percentage change in cell counts was measured on the basis of the change in IMR90 ER:RAS DAPI-positive cell numbers over 48 hours of coculture in the presence or absence of NK cells. NPA was used to normalize percentage change in cell counts between multiple plates, batches, and replicates. Sequences for siRNA candidates tested are listed in table S10.

### RNA-seq and analysis

RNA was extracted using the RNeasy Mini kit (QIAGEN) following the manufacturer’s protocol. RNA-seq was performed by the MRC LMS Genomics core facility. Library preparation was performed using a NEBNext Ultra II Directional RNA library prep kit with the NEBNext PolyA Enrichment module using the NextSeq 2000 instrument model [single-end 72 base pairs (bp) + dual 8-bp indexing for IMR90 ER:RAS and ID8 *Trp53*^−/−^ in vivo datasets, single-end 70 bp + dual 8-bp indexing for ID8 in vitro dataset, and paired-end 110 bp + dual 8-bp index for the IMR90 ER:RAS repeat elements dataset]. RNA-seq data were aligned using STAR. Differential expression was performed using the DESeq2 package on R (*67*). For GSEA, differentially expressed genes were ranked according to their expression using the pre-ranked function on GSEA v4.0.3 (Broad Institute). Significant enrichment of gene sets was considered if the normalized enrichment score was >1.0, with a false discovery rate lower than <0.25 (*68*). Gene sets used were obtained from built-in “gene ontology” or “curated” databases v7.5 (Broad Institute) or described previously in (*69*). Cluster and pathway analysis was performed using ClusterProfiler on R.

### Methods for the reanalysis of GSE151026 data

ATAC-seq peaks and genome browser bigWig tracks for BRG1/SMARCA4 ChIP-seq and ATAC-seq in BIN67 cells, with or without SMARCA4 reexpression, were obtained from the GEO database (GSE151026) and visualized using the UCSC genome browser. These datasets were originally described by Orlando *et al.* (*49*). Snapshots were taken at loci of genes of interest. ATAC-seq peaks gained upon BRG1/SMARCA4 reexpression in BIN67 cells, also retrieved from GSE151026, underwent DNA motif enrichment analysis using HOMER v4.11 with the parameter “-size given” and random background regions.

### TE RNA-seq analysis

For the TE analysis, samples sequenced using 110-bp paired-end reads were used. Reads were processed using Cutadapt v4.7 to remove Illumina adapters, trim, and filter on quality with the settings: -a AGATCGGAAGAGCACACGTCTGAACTCCAGTCA, -A AGATCGGAAGAGCGTCGTGTAGGGAAAGAGTGT, -n 1, -m 31, --nextseq-trim 20, --max-n 0. Processed reads were aligned with HISAT2 (*70*) to GRCh38 with settings adjusted to report up to 100 multiple alignments for each read pair: --rna-strandness RF--no-discordant --no-mixed --no-unal -k 100 --score-min L,0,-0.66 --pen-noncansplice 20 –dta. Gene and TE locus-level counts were obtained with TElocal [TEtranscripts; (*71*)] using settings to allow distribution of multimapping reads using maximum likelihood estimation: --stranded reverse --mode multi. GRCh38.100 (Ensembl) annotations were used for genes. TE annotations were produced using RepeatMasker v4.1.5 (*72*) configured with HMMER v3.3.2 (*73*). The GRCh38 genomes were scanned using Dfam3.7 (*74*) HMM TE models in sensitive mode: -s -no_is -norna -spec “*Homo sapiens*.” Gene and TE analysis were performed using DESeq2. TE subfamily analysis was performed by summing the counts from individual TE loci. Within DESeq2 (*67*), log_2_ fold-change shrinkage was performed using ashr (*75*). Scripts are available upon request and/or can be found at https://github.com/mpercharde/RNAseq.

### NK cell purification from mouse spleen and treatment with AU-15330

Mouse NK cells for in vitro culture experiments were purified from the mouse spleen. The mice used were wild-type C57BL/6J mice (Charles River Laboratories, UK, RRID:IMSR_JAX:000664). We used male and female mice, 10 to 16 weeks old. Spleens were collected from mice and digested in RPMI medium (Gibco, 21875-034) containing 10% of HIFBS (Sigma-Aldrich, F7524), 1% 100× penicillin/streptomycin (Gibco, 15140-122), 1% nonessential amino acids (Gibco, 11140-050), 1% sodium pyruvate (Gibco, 11360-070), and 1% l-glutamine (Gibco, 25030-024). We will refer to this medium as NK medium. Red blood cells were removed with ACK lysis buffer (Gibco, A10492-01). The cell suspension was filtered using cell strainers (Corning, 431751) and then stained using the NK cells isolation kit. NK cells were purified with magnetic separation according to the manufacturer’s protocol (Miltenyi Biotec, 130-115-818). Once NK cells were obtained, they were cultured in NK medium for 48 hours in the presence of DMSO or AU-15330 (100 nM or 1 μM). NK cells were stimulated with phorbol 12-myristate 13-acetate (PMA; 50 nM; Sigma-Aldrich, catalog no. P1585) and ionomycin (0.5 μg/ml; Sigma-Aldrich, I0634) for 2 hours, followed by 2 hours of brefeldin A (10 μg/ml; Enzo Life Sciences, BML-G405-0005). Then, NK cells were collected, and RNA was extracted using TRIzol (Ambion, Life Technologies, 15596026) according to the manufacturer’s protocol.

### ID8 *Trp53*^−/−^ model of ovarian cancer

For the ovarian cancer experiments, C57BL/6J female mice were bought from Charles River Laboratories, UK (RRID:IMSR_JAX:000664), at 7 to 8 weeks of age and left to acclimatize for at least 1 week before any regulated procedures were performed. ID8 *Trp53*^−/−^ cells (5 × 10^6^) per mouse were injected in sterile and filtered PBS (Thermo Fisher Scientific, J61196-AP) intraperitoneally in C57BL/6 J. After 1 month, mice were randomized in four different experimental treatment groups. For the experiment in Figs. 7 and 8 (F to H) and figs. S15 and S16 (G to K), mice were treated with vehicle, cisplatin (4 mg/kg, once a week for 1 week), and AU-15330 (60 mg/kg, three times a week for 2 weeks) alone or in combination. All the drugs were delivered to the mice intraperitoneally.

For the experiment in Fig. 8 (A to E) and fig. S16 (A to F), mice were treated with vehicle, cisplatin (4 mg/kg, once a week for 1 week) and AU-15330 (30 mg/kg, three times a week for 2 weeks) alone or in combination. Vehicle and AU-15330 were delivered to the mice intravenously (tail vein injection), while cisplatin was delivered to the mice intraperitoneally. At the defined end point, omental tumors and spleens were collected. Cisplatin was dissolved in PBS. AU-15330 was purchased from MedChemExpress (catalog no. HY-145388), dissolved in 40% of 2-hydroxypropyl-β-cyclodextrin (HPβCD) and sonicated until completely dissolved, and then mixed with 5% dextrose in water to reach a final concentration of 10% HPβCD (Figs. 7 and 8, F to H, and figs. S15 and S16, G to K). AU-15330 was dissolved in 5% DMSO and 95% (20% sulfobutylether-β-cyclodextrin in saline) (Fig. 8, A to E, and fig. S16, A to F).

In the NK depletion experiments, after 1 month from cell injection, mice were randomized into two experimental groups and treated, respectively, with cisplatin in combination with AU-15330 + IgG and cisplatin in combination with AU-15330 + αNK1.1. Cisplatin and AU-15330 were administered as previously described, while αIgG and αNK1.1 were administered intraperitoneally twice a week for 2 weeks (250 μg per mouse). At the defined end point, omental tumors and spleens were collected. NK-depleting antibody (αNK1.1, clone PK136, catalog no. BE0036) and IgG isotype control (IgG2a, clone C1.18.4, catalog no. BE0085) were purchased from BioXcell and diluted in PBS.

### Liver cancer initiation

Hydrodynamic tail vein injection (HDTVI) was carried out in female C57BL/6J (Charles River Laboratories, UK, RRID:IMSR_JAX:000664) mice aged 5 to 6 weeks using 25 μg of a transposon expressing NRAS^G12V^ along with 5 μg of SB13 transposase-expressing plasmid. Plasmids were prepared with a GenElute HP Endotoxin-Free Maxiprep kit (Sigma-Aldrich). For HDTVI, vectors were diluted in normal saline to a final volume of 10% body weight. HDTVI was performed within 7 to 8 s.

### IHC staining of tissue sections

Omental tumor tissue sections were deparaffinized in Histoclear (Scientific Laboratory Supplies) for 5 min and washed in decreasing concentrations of ethanol, until a final 5-min wash in distilled H_2_O. Heat-induced epitope retrieval was then performed in a pressure cooker for 20 min in citrate-based at pH 6.0 (VectorLab, H-3300-250) or tris-based at pH 9.0 (VectorLabs, H-3301-250), following the antibody manufacturer’s instructions. For intracellular expression stains, sections were permeabilized in Triton X-100 (0.2% in PBS) for 10 min and washed in PBS for 5 min. Slides were then incubated in BLOXALL blocking solution (VectorLabs, SP-6000) for 15 min, washed in PBS, and exposed to Animal-Serum Free serum (Cell Signaling Technology, 15019L) diluted in dH_2_O for 45 min. Slides were then incubated with primary antibody overnight in a humidified chamber at 4°C. Slides were washed twice in PBS for 5 min and incubated with a secondary antibody for 45 min. After, slides were washed in PBS and incubated in SignalStain DAB (Cell Signaling Technology, 8059) for 5 min or until the horseradish peroxidase signal was visible and the reaction stopped in dH_2_O. Cells were then stained for hematoxylin (Dako, Mayer’s Hematoxylin, S3309) for 30 s and washed in dH_2_O. Slides were dehydrated in 75% ethanol for 1 min and 100% ethanol for 5 min, washed in Histoclear for 5 min, and mounted in dibutylphthalate polystyrene xylene (DPX) (Sigma-Aldrich). Slides were imaged using 20× bright-field objective on Zeiss AxioScan Z.1 slide scanner. QuPath was used to quantify staining by measuring the positive cells as a percentage of the total tissue area. All antibodies used in IF and immunohistochemistry (IHC) are listed in tables S5 and S7.

### IF staining of tissue sections

For IF staining, deparaffinization and antigen retrieval were performed as described previously (see the “IHC staining of tissue sections” section). Tissue samples were incubated overnight in the primary antibody previously diluted in antibody diluent (Dako). Samples were washed in PBS three times for 5 min. Samples were then incubated in secondary antibody for 45 min. To perform double staining, samples were incubated in HCl (0.02 mol/l) for 20 min after the first antibody signal amplification step. Samples were then washed in PBS for 5 min, and peroxidase blocking was reapplied for 20 min. Samples were then incubated in Animal-Serum Free blocking solution (Cell Signaling Technology, 15019L) diluted in H_2_O for 1 hour, and the second primary antibody incubation was performed overnight. Samples were washed in PBS three times for 5 min. Samples were then incubated in secondary antibody for 45 min. Then, the tyramide SuperBoost kit (Invitrogen, B40935 for Alexa Fluor 596 and B40932 for Alexa Fluor 488) is prepared according to the manufacturer’s instructions and added to the section for 10 min at room temperature. Samples were then washed three times in PBS for 5 min and incubated with DAPI (1 μg/ml; w/v, PBS) for 5 min. Samples were washed a further three times in PBS for 5 min and mounted in 50% glycerol in PBS. Slides were imaged using 20× fluorescence objective on Zeiss AxioScan Z.1 slide scanner. QuPath was used to quantify staining by measuring positive cells as a percentage of the total tissue area.

### IF staining of HGSOC TMAs

The TMA was sectioned into 4-μm slices and stained using a BenchMark XT automated stainer (Ventana Medical System Inc., Tucson, AZ, USA). Antigen retrieval was carried out with Cell Conditioning 1 (Ventana Medical System Inc., no. 950-124) for 60 min. The prediluted primary antibody was automatically dispensed and incubated for 60 min at 37°C. The following steps were performed manually. After blocking with Protein Block serum-free reagent (Dako, no. X0909), secondary antibodies were added for 45 min, followed by washing and blocking overnight at 4°C with diluted Mouse-On-Mouse reagent (VectorLabs, no. MKB-2213). This was followed by an incubation of 60 min with primary antibodies of the epithelial mask. The Alexa Fluor 750 secondary antibody was incubated for 45 min, followed by DAPI staining for 5 min. Staining with 0.1% Sudan black for 15 min to quench tissue autofluorescence was followed by coverslip mounting using Fluoromount Aqueous Mounting Medium (Sigma-Aldrich, no. F4680). The full TMA was scanned and digitalized using a 20× 0.75 numerical aperture objective with a resolution of 0.325 μm (BX61VS, Olympus). Multicolor images were segmented and quantified using Visiopharm Integrator System (VIS) version 2023.09.7.16662 (Visiopharm, Denmark).

### TMA VIS data acquisition and analysis

Briefly, the VIS software was used to measure the MFI of all pixels in original digitalized multicolor TMA images for a chosen region of a tissue sample (total tissue core or core sub-compartments as described for fig. S14B). Then, the MFIs corresponding to replicate total cores or selected core sub-compartments from the same patient are averaged to calculate one final mean MFI for each data point used in the presented analysis. Controls related to SMARCA4 duplicate core reproducibility are presented in fig. S14C. For single-cell analysis, individual nuclei in each core were identified and assigned SMARCA4 positivity-negativity. VIS was also used to analyze immune infiltration in HGSOC tissues where a cell body area was determined around each nucleus, which was then used to assign CD3 positivity (T cells) and CD68 positivity (macrophages; fig. S14, G and H). Statistical significance of differences in MFI was calculated using the Mann-Whitney test. Survival curves were plotted using Kaplan-Meier analyses, and the log-rank test was used to test for significance. All statistical analyses were done using the Statistical Package for Social Sciences software version 21 (SPSS Inc.), and statistical significance was set at *P* < 0.05. Some patients were excluded from the final analysis on the basis of poor quality cores (folded or scratched cores) or for lack of epithelial material in cores.

### Flow cytometry

Tumors were disaggregated and digested in collagenase type I (Thermo Fisher Scientific, 17100017) and DNase (Sigma-Aldrich, D4527) for 45 min at 37°C to obtain a single-cell suspension. The obtained cell suspension was filtered with a cell strainer (70 μm; Corning, 431751). After neutralization of unspecific binding with anti-CD16/CD32 antibody (BioLegend, 101302), single-cell suspensions were stained with specific monoclonal antibodies (primary antibodies directly conjugated) to assess the phenotype and diluted 1:200. The antibodies used are listed in table S6. For IFN-γ and granzyme B staining, tumors and cells were stimulated with PMA (50 nM, Sigma-Aldrich, catalog no. P1585) and ionomycin (0.5 μg/ml; Sigma-Aldrich, I0634) for 2 hours, followed by 2 hours of brefeldin A (10 μg/ml; Enzo Life Sciences, BML-G405-0005). Cells were then permeabilized using the Fix/perm kit (Life Technologies, GAS004) according to the manufacturer’s instructions. For flow gating, we used isotype controls of fluorescence minus one control. Samples were acquired on a BD symphony flow cytometer (BD Biosciences). Data were analyzed using FlowJo software (TreeStar). CountBright Plus Absolute Counting Beads (Invitrogen, C36950) according to the manufacturer’s instructions were used to infer the number of immune cells.

### Inference of NK cells and SMARCA4 association in the OCTIPS cohort

Transcript-per-million (TPM) data were generated from raw counts using the GRCh37.87 reference GTF file with batch correction performed using ComBat-seq (*76*). TPM counts were filtered to ensure >0.5 TPM in more than half the samples, and a mean value was taken where duplicate values for gene expression existed. Immune infiltration was estimated from filtered TPM counts using the CIBERSORTx (*51*) platform in absolute mode with 100 permutations, batch correction, and quantile normalization disabled. For use in gene set scoring, MSigDB (*77*) was mined for gene sets relating to NK cell activity. Filtered TPM counts were used to score samples for these NK cell gene sets, via the singscore package (*78*). Correlations between SMARCA4 TPM and inferred NK cell infiltration, or NK cell gene set scores, were calculated and visualized in RStudio with the ggpubr package, using Spearman’s correlation.

### Inference of NK cells and SMARCA4 association in the TCGA-OV cohort

RNA data in the form of unstranded TPM for the TCGA ovarian (TCGA-OV) cohort (*n* = 429 samples) were downloaded using the GDCquery function from within the R package TCGABioLinks (*79*), accessed 5 March 2023. Filtering was performed as described for the OCTIPS dataset, resulting in 422 unique TCGA-OV samples after aggregation. Tumor purity data were available for 412 of these patients, obtained from the supplementary data of (*80*). An identical pipeline for immune infiltration estimation and correlation analyses was used as described for the OCTIPS dataset. For partial correlation analyses, the stats package in R was used to perform multiple linear regression and calculate residuals ahead of plotting with the ggpubr package.

### Inference of NK cells, purity, and SMARCA4 association in the TRACERX cohort

TRACERx [TRAcking non–small cell lung Cancer Evolution through therapy (Rx); clinical trial identifier NCT01888601] is a longitudinal prospective cohort study, designed to delineate tumor evolution from diagnosis and surgical resection to either cure or disease recurrence (*52*). TPM data and Danaher scores and purity were calculated as previously described (*52*). Correlations between SMARCA4 log_2_(TPM) values, NK cell Danaher scores, and purity values were calculated and visualized in RStudio, using Spearman’s correlation.

### Statistics and reproducibility

Statistical analyses were performed and plotted using GraphPad Prism 9 software. Details of the test used are given in the corresponding figure legends and the source data. Statistical analysis was performed using either an unpaired, two-tailed *t* test or with ordinary one- or two-way analysis of variance (ANOVA) with Dunnett’s, Šídák’s, or Tukey’s multiple comparisons correction. All data points are plotted together with an overlay of the means ± SD or SEM (as specified in the figure legends). *P* < 0.5 was taken to indicate statistical significance. The number of biological replicates is indicated in their respective figure legends. No statistical methods were used to predetermine sample sizes for the in vivo studies, but our sample sizes are similar to what we have reported previously, which aimed to reach a statistical power of at least 80%. For in vivo studies, mice were randomized to treatment groups 1 month after injecting the tumor cells. Cell culture experiments were not randomized. Investigators were not blinded during the cell culture or in vivo experiments, but, whenever possible (e.g., quantitative IF and IHC analysis), analysis was performed using automated procedures. Flow cytometry samples were excluded from analysis when the number of acquired events was less than 1000.
