# Supplementary material for: SMARCA4 regulates the NK-mediated killing of senescent cells
Source: Sci Adv. 2025 Jan 15;11(3):eadn2811. doi: 10.1126/sciadv.adn2811 (PMC11734740; doi:10.1126/sciadv.adn2811)
Supplement: Supplementary file 1 — Figs. S1 to S16 Legends for tables S1 to S3 and S11 Tables S4 to S10 [file sciadv.adn2811_sm.pdf]

Science Advances
AAAS

Supplementary Materials for

SMARCA4 regulates the NK-mediated killing of senescent cells

Virinder Reen *et al.*

Corresponding author: Jesús Gil, jesus.gil@imperial.ac.uk

Sci. Adv. **11**, eadn2811 (2025)
DOI: 10.1126/sciadv.adn2811

The PDF file includes:

Figs. S1 to S16
Legends for tables S1 to S3 and S11
Tables S4 to S10

Other Supplementary Material for this manuscript includes the following:

Tables S1 to S3 and S11

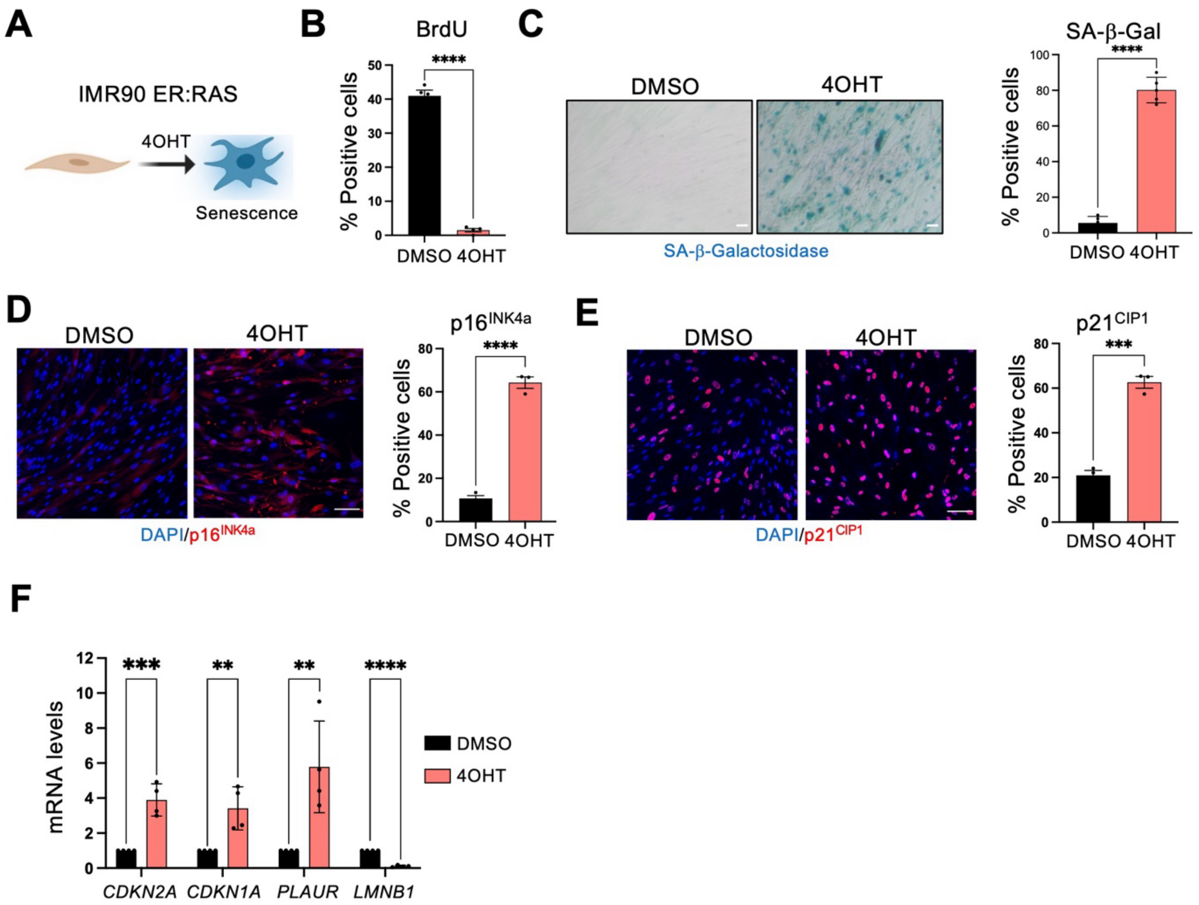

Figure S1. IMR90 ER:RAS as a model to study oncogene-induced senescence (OIS). (A) IMR90 ER:RAS cells as a model of oncogene-induced senescence (OIS). IMR90 fibroblasts infected with the ER:RAS construct undergo OIS upon the addition of 4OHT. **(B)** Quantification of immunofluorescence (IF) staining for BrdU incorporation in IMR90 ER:RAS cells 8 days after treatment with DMSO or 4OHT. Data represents mean ± SEM (n = 3). ****p < 0.0001; unpaired t-test. **(C)** Quantification of SA-β-galactosidase (SA-β-gal) activity in IMR90 ER:RAS cells. Left panel, representative brightfield images for SA-β-gal staining in IMR90 ER:RAS cells 7 days following treatment with DMSO or 4OHT. Scale bar, 100μm. Right panel, quantification of the percentage of cells staining positive for SA-β-gal. Data represents mean ± SEM (n = 5). ****p < 0.0001; unpaired t-test. **(D-E)** IF staining for senescence markers. Left panel, representative IF images for p16^{INK4a} staining (D) and p21^{CIP1} (E) in IMR90 ER:RAS cells 8 days following treatment with DMSO or 4OHT. Scale bar,100 μm. Right panel, quantification of the percentage of cells staining positive for p16^{INK4a} (D) and p21^{CIP1} (E). Data represents mean ± SEM (n =

3). ***p < 0.001; ****p < 0.0001; unpaired t-test. **(F)** qRT-PCR analysis of indicated senescence markers in IMR90 ER:RAS cells 8 days following treatment with DMSO or 4OHT (n = 3). **p < 0.01; ****p < 0.0001; unpaired t-test.

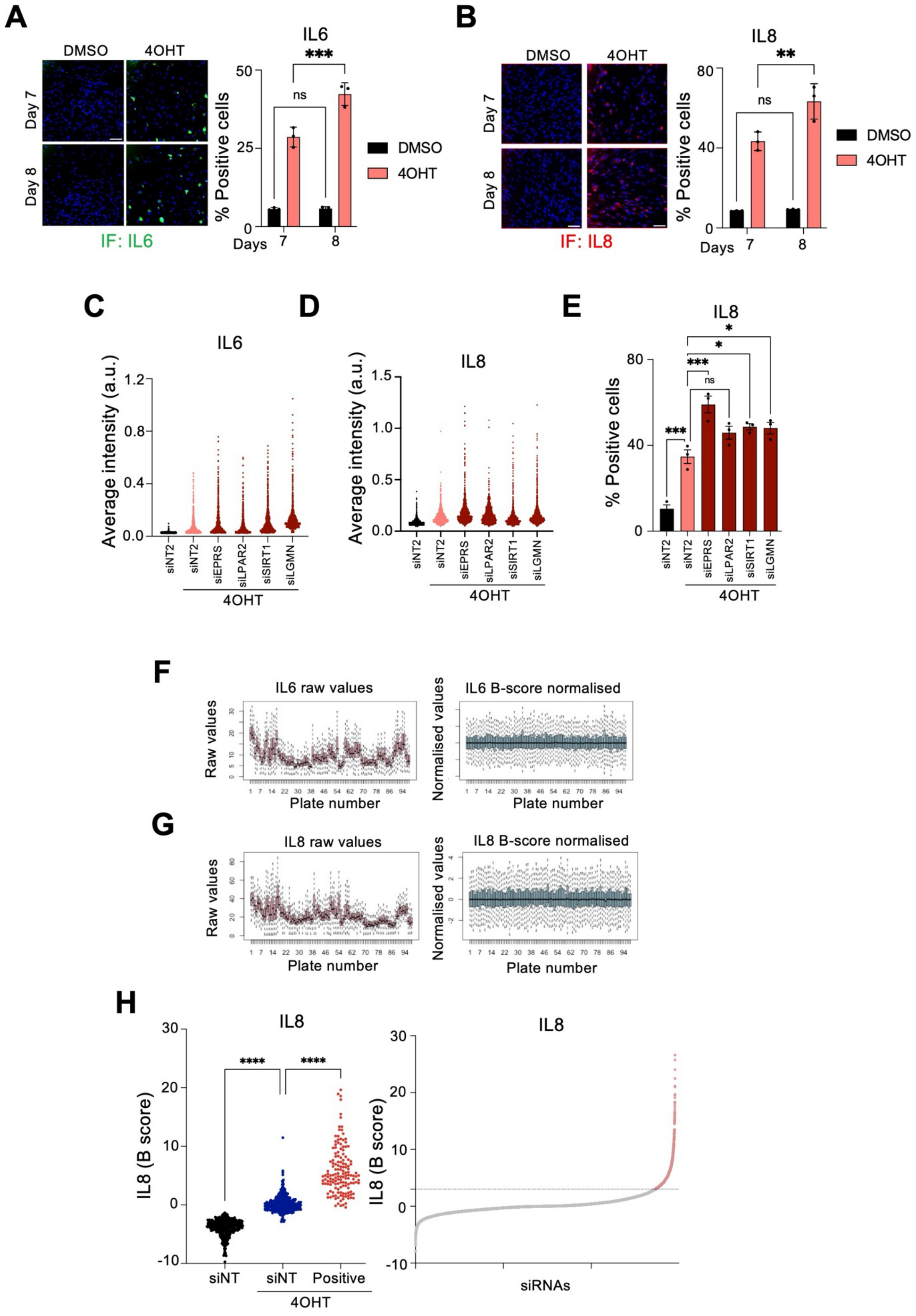
A
IL6
DMSO
4OHT
Day 7
Day 8
IF: IL6
% Positive cells
Days
DMSO
4OHT
ns
***
B
IL8
DMSO
4OHT
Day 7
Day 8
IF: IL8
% Positive cells
Days
DMSO
4OHT
ns
**
C
IL6
Average intensity (a.u.)
siNT2
siEPRS
siLPAR2
siSIRT1
siLGMN
4OHT
D
IL8
Average intensity (a.u.)
siNT2
siEPRS
siLPAR2
siSIRT1
siLGMN
4OHT
E
IL8
% Positive cells
siNT2
siEPRS
siLPAR2
siSIRT1
siLGMN
4OHT
F
IL6 raw values
IL6 B-score normalised
Raw values
Normalised values
Plate number
G
IL8 raw values
IL8 B-score normalised
Raw values
Normalised values
Plate number
H
IL8
IL8 (B score)
siNT
Positive
4OHT
****
siRNAs

Figure S2. Screens to identify siRNAs superinducing the SASP in IMR90 ER:RAS cells. (A-B) Representative immunofluorescence (IF) images (left panel) and quantification (right panel) of the percentage of IL6 **(A)** and IL8 **(B)** positive IMR90 ER:RAS cells at days 7 and 8 following senescence induction. Scale bar, 100 μm. Data represents mean ± SD (n = 3). $^{**}p < 0.01$; $^{***}p < 0.001$; ns, not significant; ordinary one-way ANOVA (Tukey's multiple comparisons test). **(C-D)** Quantification of IF displaying the average intensity values of IL6 **(C)** and IL8 **(D)** per cell in a representative sample well seeded with IMR90 ER:RAS cells transfected with the specified control siRNAs. **(E)** IF quantification in the percentage of IMR90 ER:RAS cells positive for IL8 expression beyond a pre-determined threshold following transfection with the indicated siRNAs. Data represents mean ± SD (n = 3). $^{*}p<0.05$; $^{***}p <0.001$; ns, not significant; ordinary one-way ANOVA (Dunnett's multiple comparisons test). **(F-G)** B-score normalisation for IL6 **(F)** and IL8 **(G)** from the siRNA SASP superinducer screen. The distribution of the normalisation within each screen plate is displayed as an individual box-and-whisker plot. The left panel shows the raw B-score value of IL6 or IL8 for plates with siRNA-transfected IMR90 ER: RAS cells. The right panel shows the normalised B-score values for each screened plate. Displayed are the representative pre- and post-normalisation distributions for one of the two biological replicates of the siRNA screen. (**H**) Results of the siRNA screen for IL8 superinducers. The left panel represents the normalised score for the control siRNAs. The right panel displays the normalised score for each sample siRNA pool. The dotted line indicates the threshold beyond which hits were selected. $^{****}p < 0.0001$; ordinary one-way ANOVA (Tukey's multiple comparisons test).

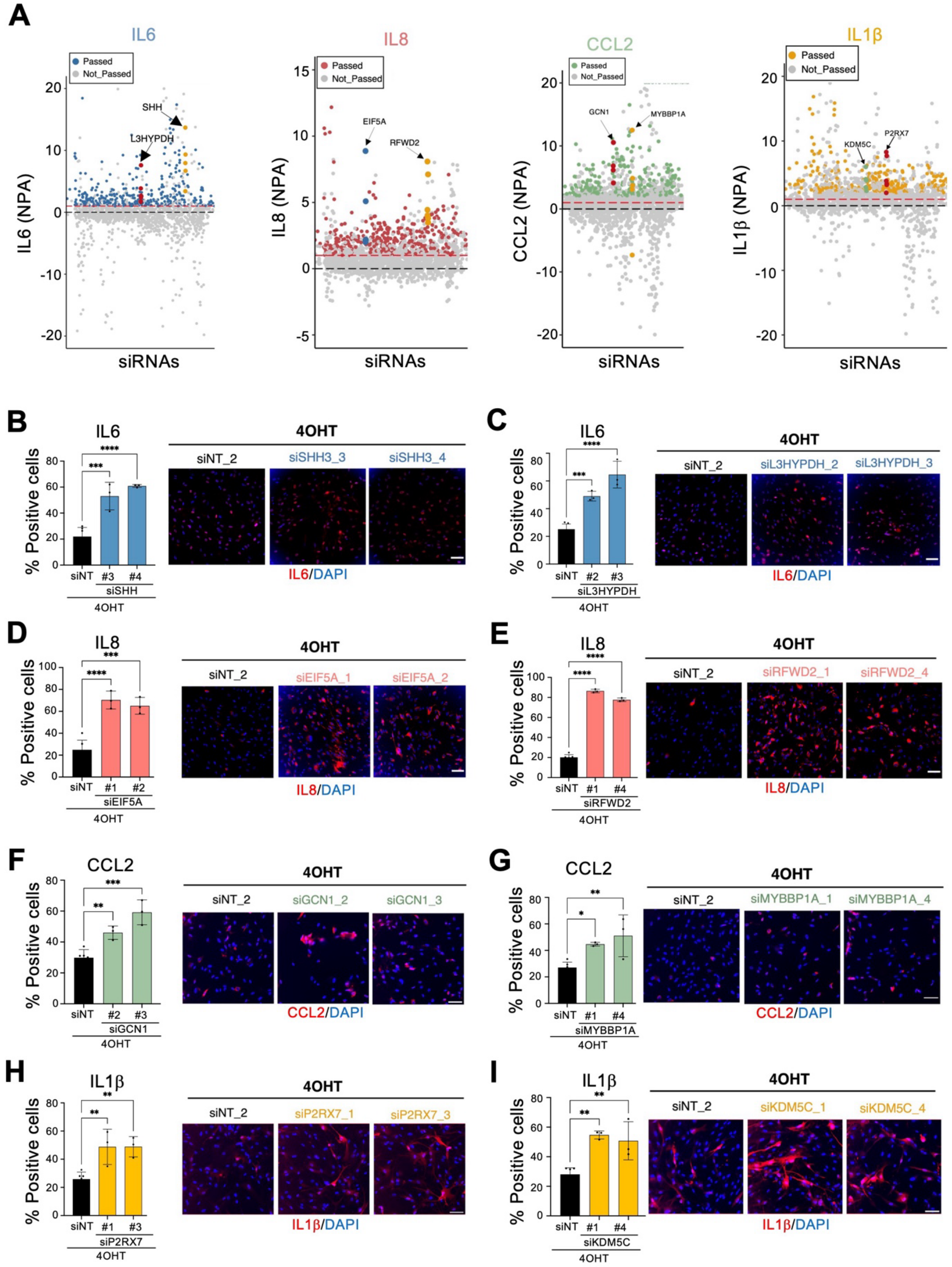

Figure S3. Representative hits identified in the screen for SASP regulators. (**A**) Results of the secondary siRNA screen for superinducers of the indicated SASP

readouts. Normalised NPA scores of the SASP readouts are shown for each replicate siRNA sample. The black dotted line represents the normalised score of the negative non-targeting siRNA controls (NPA = 0). The red dotted line represents the normalised score of positive siRNA controls (NPA = 1). For IL6 and IL8, the threshold for hit selection was NPA ≥ 1. For CCL2 and IL1β, the threshold was set at NPA ≥ 2. Hits were selected if at least 2 of 4 siRNA targeting a gene scored beyond the specified threshold in at least 2/3 replicates. Two examples of genes whose knockdown results in elevated expression of each SASP readout are shown **(B-I).** Quantification and representative IF images for IL6 **(B-C)**, IL8 **(D-E)**, CCL2 **(F-G)** and IL1β **(H-I)** in senescent IMR90 ER:RAS cells transfected with the indicated siRNAs. Scale bar, 100 μm. Data represents mean ± SEM (n = 3). *p < 0.05; **p < 0.01; ***p <0.001; ****p <0.0001; ordinary one-way ANOVA (Tukey's multiple comparisons test).

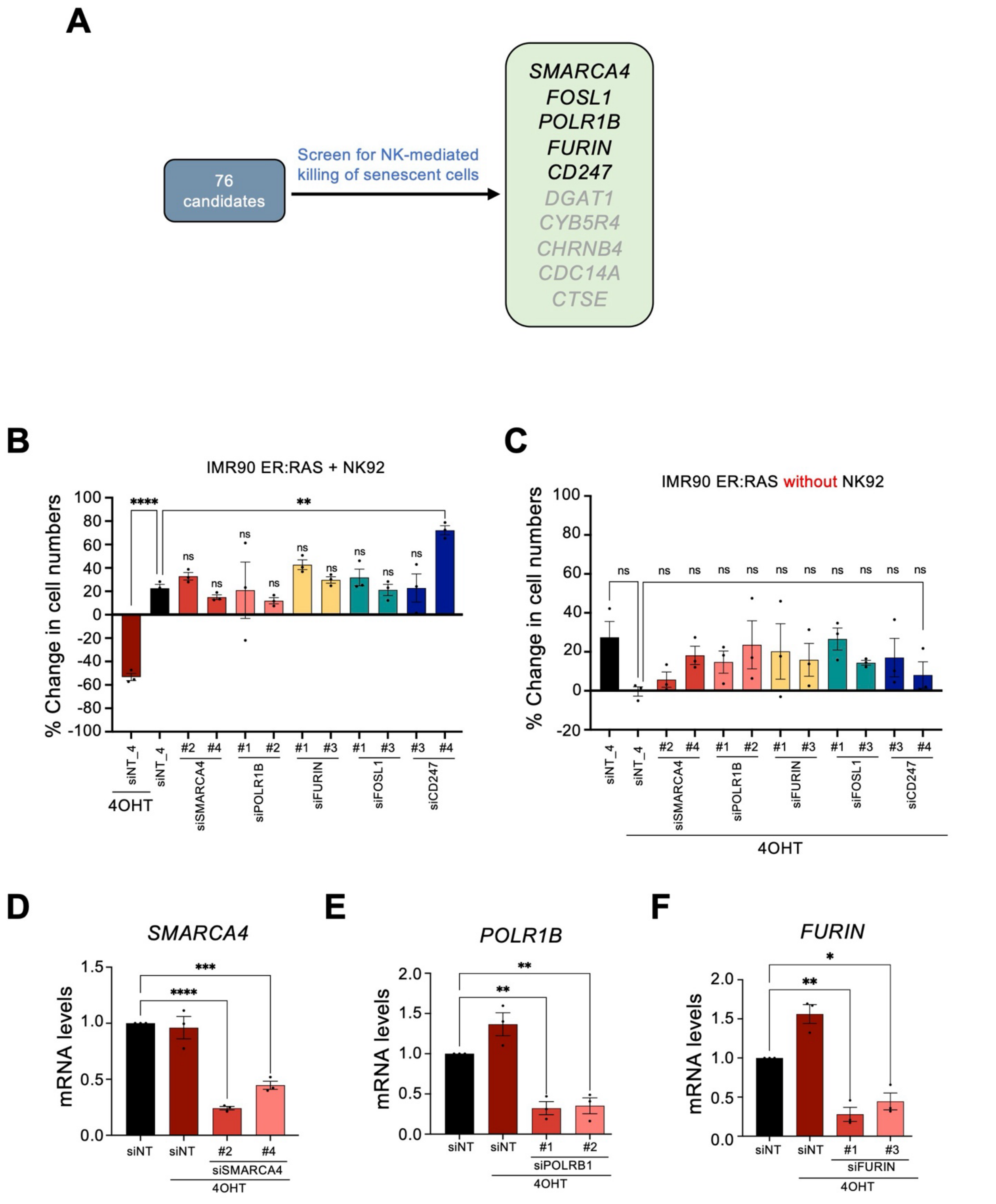

Figure S4. Regulators of NK-mediated killing of senescent cells. (A) List of ten gene candidates selected as hits from the siRNA screen to identify reinforcers of NK-mediated killing of senescent cells. The five genes retested are shown in black. **(B)** Percentage

change in IMR90 ER:RAS cells transfected with the indicated siRNA and treated with DMSO after co-culture with NK cells at a 2:1 E:T ratio. Data represents mean ± SEM (n = 3). **p < 0.01; ****p < 0.0001; ns, not significant; ordinary one-way ANOVA (Dunnett's multiple comparisons test). **(C)** Percentage change in IMR90 ER:RAS cells transfected with the indicated siRNA and treated with 4OHT in the absence of NK cells. Data represents mean ± SEM (n = 3). ns, not significant; ordinary one-way ANOVA (Dunnett's multiple comparisons test). **(D-F)** qRT-PCR analysis showing the mRNA expression of SMARCA4 **(D)** POLR1B **(E)** and FURIN **(F)** in IMR90 ER:RAS cells 3 days after being transfected with two selected siRNAs against the indicated genes. Data represents mean ± SEM (n = 3). *p < 0.05; **p < 0.01; ***p <0.001; ****p <0.0001; ordinary one-way ANOVA (Tukey's multiple comparisons test).

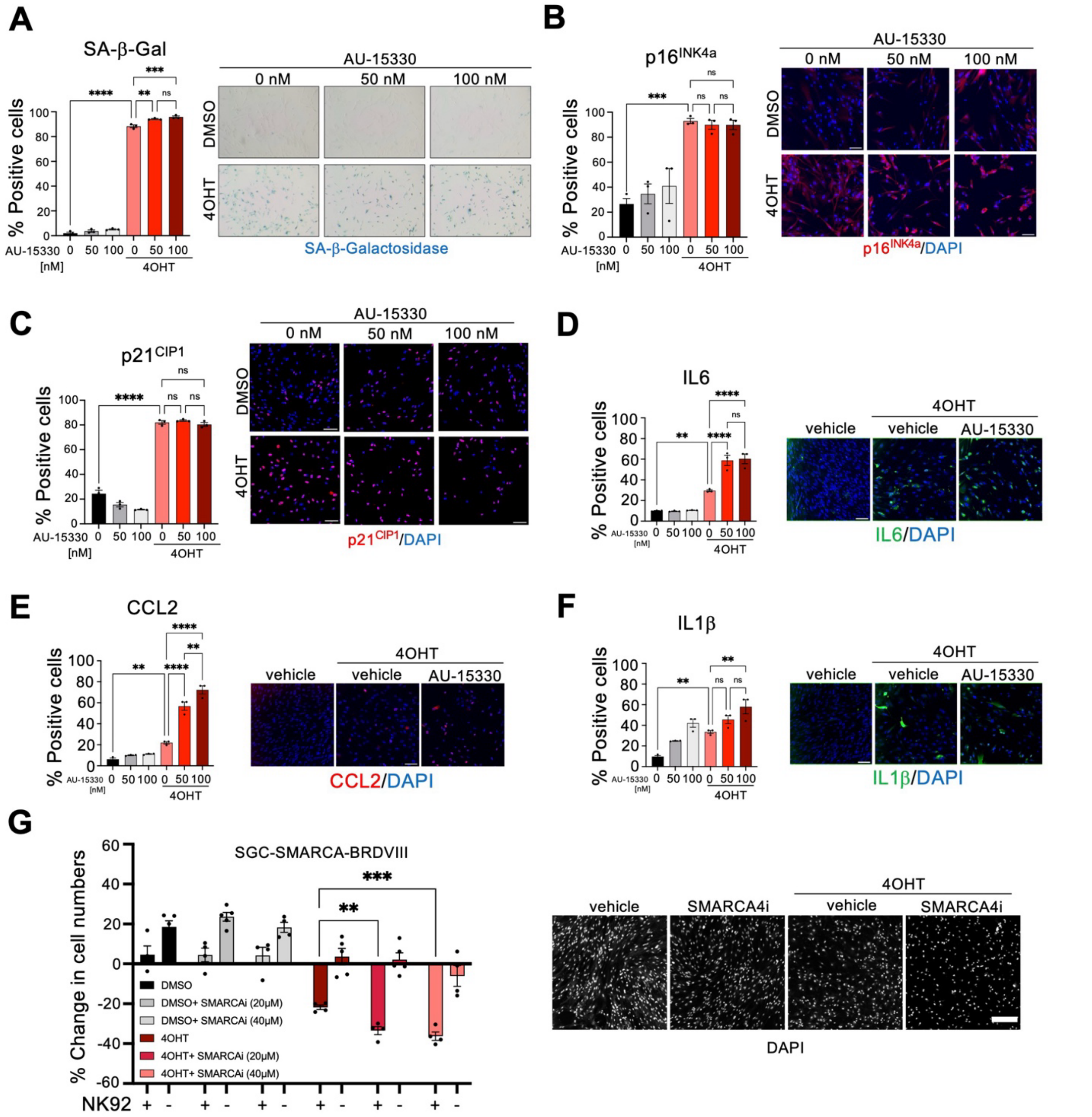

Figure S5. Effects of SMARCA4 inhibitors in a model of oncogene-induced senescence (OIS). (A) Quantification (left panel) and representative images right panel) of SA-β-gal activity in DMSO or 4OHT-induced IMR90 ER:RAS cells treated with AU-15330 at the indicated concentrations. Scale bar, 100 μm. Data represents mean ± SEM (n = 3). $^{**}p < 0.01$; $^{***}p<0.001$; $^{****}p < 0.0001$; ns, not significant; ordinary one-way ANOVA (Tukey's multiple comparisons test). **(B-F)** IF staining for senescence markers. Left panel, quantification of the percentage of cells staining positive for p16^{INK4a} (B),

p21^{CIP1} (C), IL6 (D), CCL2 (E) and IL1β (F) in IMR90 ER:RAS cells at day 7 after DMSO or 4OHT induction, and treated with the indicated concentrations of AU-15330 at day 4. Right panel, representative IF images. Scale bar,100 μm. Data represents mean ± SEM (n = 3). **p<0.01; ****p < 0.0001; ns, not significant; ordinary one-way ANOVA (Tukey's multiple comparisons test). **(G)** Quantification (left panel) and representative images (right panel) of NK-mediated killing of DMSO or 4OHT-induced IMR90 ER:RAS cells treated with the SMARCA4 inhibitor, SGC-SMARCA-BRDVIII at the indicated concentrations. NK cells were added at a 1:1 E:T ratio for 48 hours. Scale bar, 300 μm. Data represents mean ± SEM (n = 4). **p < 0.01; ***p < 0.001; ordinary one-way ANOVA (Tukey's multiple comparisons test).

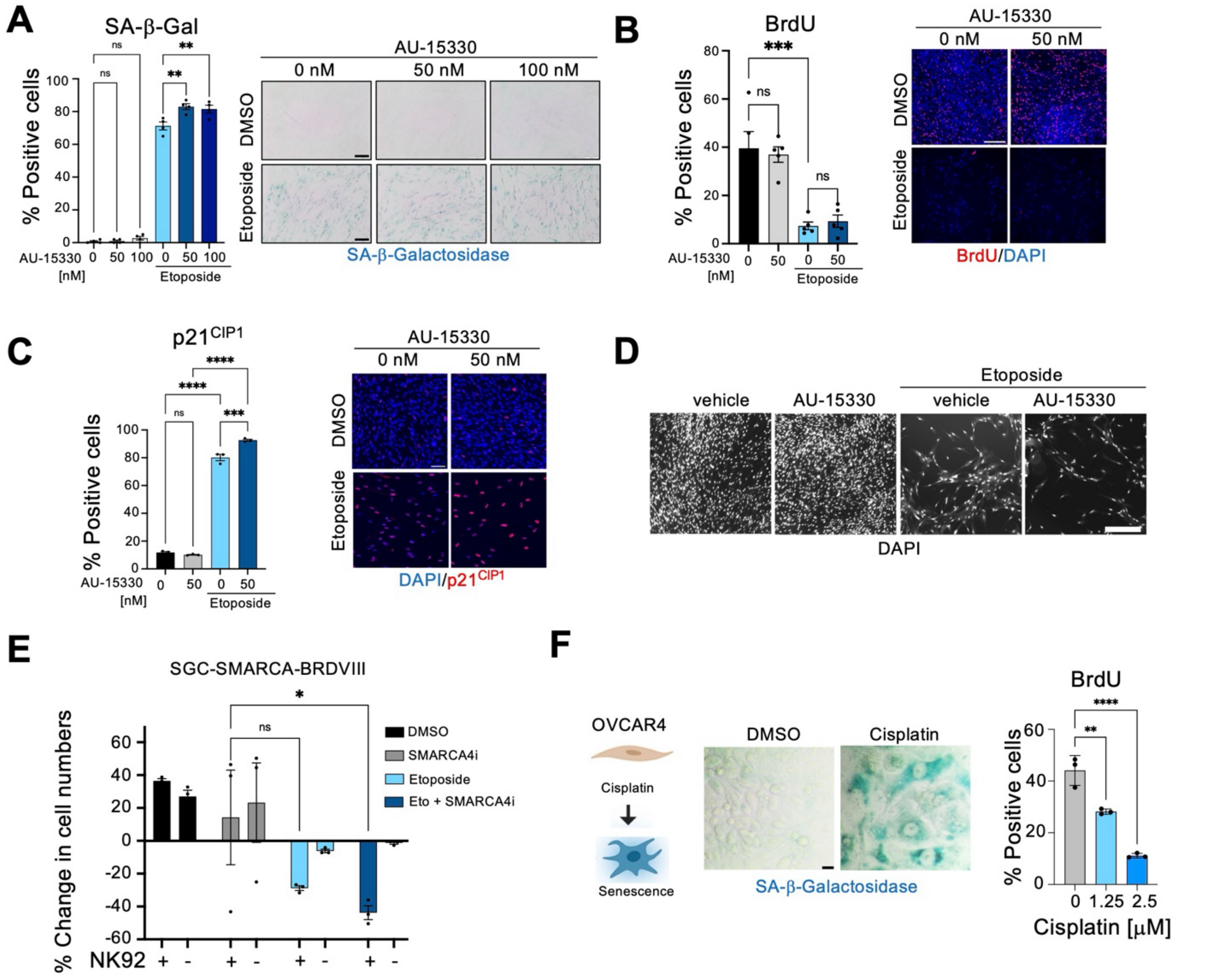

Figure S6. Effects of SMARCA4 inhibitors in therapy-induced senescence (TIS). (A) Quantification (left panel) and representative images (right panel) of SA-β-gal activity in DMSO or etoposide-induced IMR90 cells. Control or senescent IMR90 cells were treated with AU-15330 at day 4 at the indicated concentrations. Scale bar, 100 μm. Data represents mean ± SEM (n = 3). $^{**}p < 0.01$; ns, not significant; ordinary one-way ANOVA (Tukey's multiple comparisons test). **(B-C)** IF staining for senescence markers. Left panel, quantification of the percentage of cells staining positive for BrdU (B) and $p21^{CIP1}$ (C) at day 7 in DMSO or etoposide-induced IMR90 cells which were treated with the 50 nM of AU-15330 at day 4. Right panel, representative IF images for BrdU staining (B) and $p21^{CIP1}$ (C). Scale bar, 100 μm. Data represents mean ± SEM (n = 3). $^{***}p < 0.001$; $^{****}p < 0.0001$; ns, not significant; ordinary one-way ANOVA (Tukey's multiple comparisons test). **(D)** Representative images of DMSO or etoposide-induced IMR90 cells treated in

the presence or absence of 50 nM AU-15330, after 48 hours of co-culture with NK cells. Scale bar, 300 μm. **(E)** Quantification of NK-mediated killing of DMSO or etoposide-induced senescent IMR90 cells and treatment with the SMARCA4 inhibitor, SGC-SMARCA-BRDVIII, at the indicated concentrations. NK cells were added at a 2:1 E:T ratio for 48 hours. Data represents mean ± SEM (n = 3). *p < 0.05; ns, not significant; two-way ANOVA (Tukey's multiple comparisons test). (**F**) SA-β-gal activity in cisplatin-induced OVCAR4 cells. Middle panel, representative SA-β-gal IF images of OVCAR4 cells treated with Cisplatin for 6 days to induce senescence. Scale bar, 20 μm. Right panel, quantification of the percentage of cisplatin-treated OVCAR4 cells positive for BrdU. Data represents mean ± SD (n = 3). **p < 0.01; ****p < 0.0001; ordinary one-way ANOVA (Dunnett's multiple comparisons test).

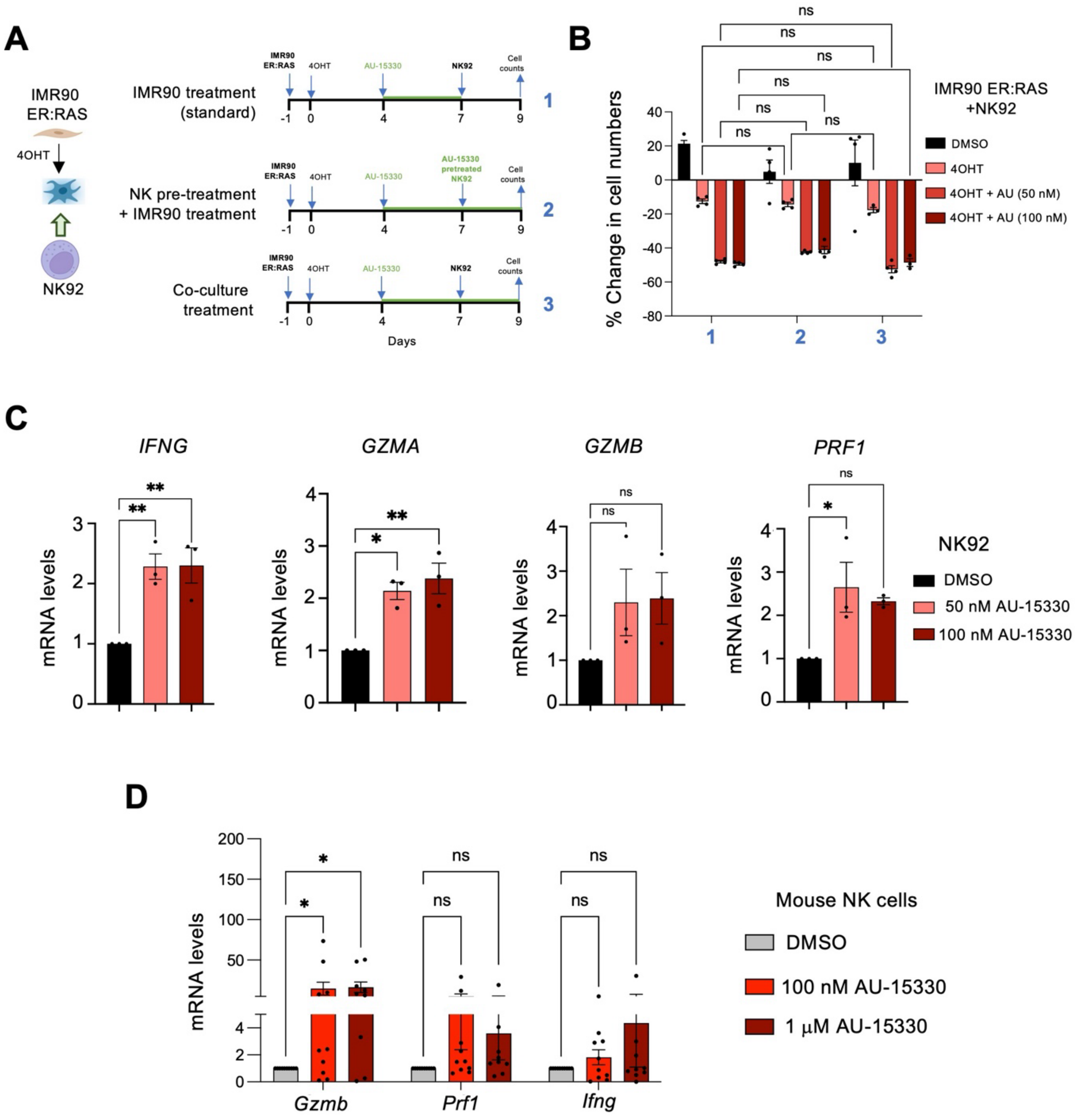

Figure S7. Effect of the SMARCA4 inhibitor AU-15330 in NK cells. (A) Schematic of the experimental settings of the NK cell co-culture with IMR90 ER:RAS cells. Co-cultures were performed with NK92 cells pre-treated with or without AU-15330 as indicated. **(B)** Quantification of the percentage change in IMR90 ER:RAS cell counts following co-culture with NK cells pre-treated with AU-15330 or not, as shown in **(A)**. Data represents mean ± SEM (n = 4). ns, not significant; two-way ANOVA (Tukey's multiple comparisons test). **(C)** mRNA expression levels of *IFNG, GZMA, GZMB* and *PRF1* in NK92-MI measured by qRT-PCR. Data represents mean ± SEM. *p<0.05; **p<0.01; ns, not significant; one-way ANOVA (Dunnett's multiple comparisons test). **(D)** mRNA expression

levels of *Gzmb, Prf1* and *Ifng* in NK cells purified from mouse spleen measured by qRT-PCR. Data represents mean ± SD. *$p<0.05$; ns, not significant; two-way ANOVA (Tukey's multiple comparisons test).

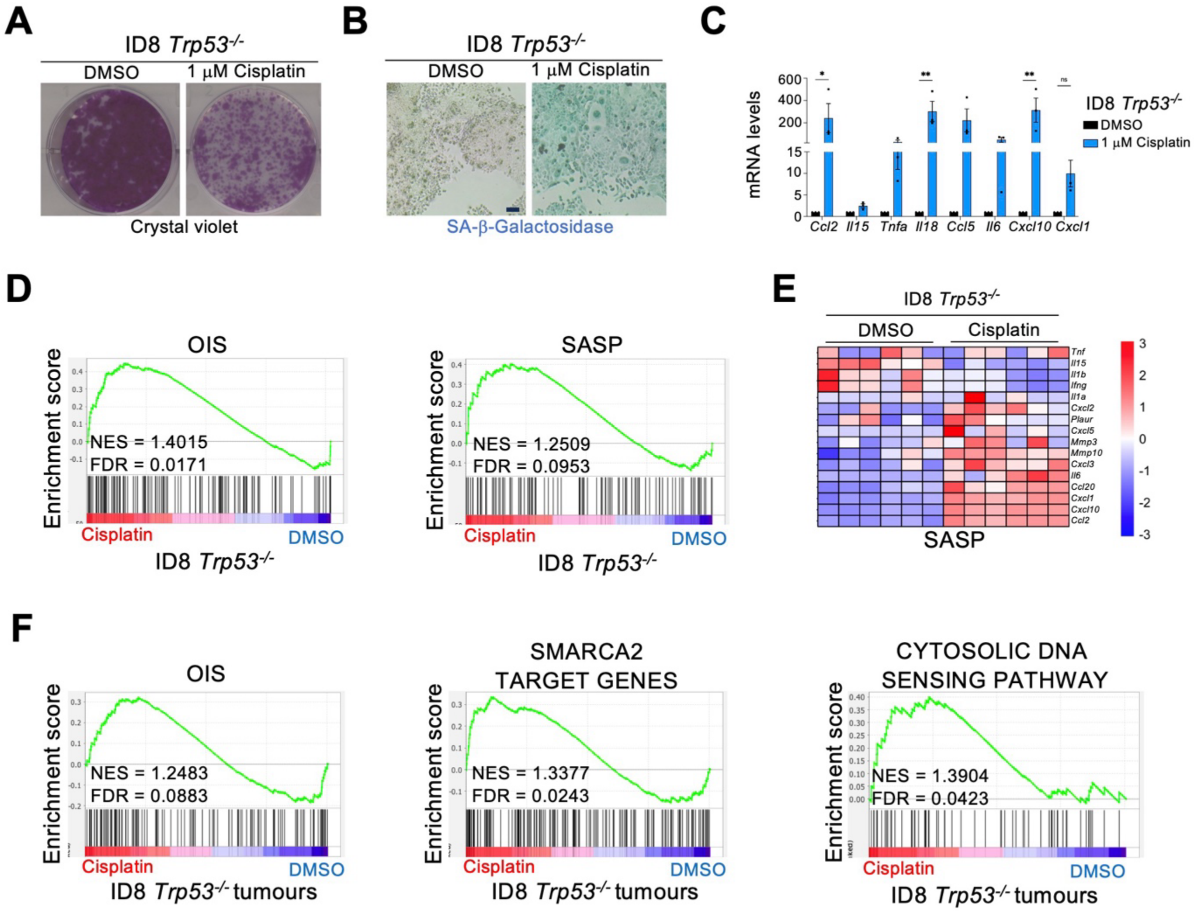

Figure S8. Cisplatin-induced senescence in ID8 *Trp53*$^{-/-}$ cells and tumours. (**A**) Representative crystal violet images of ID8 *Trp53*$^{-/-}$ cells DMSO or cisplatin-treated. (**B**) Representative SA-β-gal pictures of ID8 *Trp53*$^{-/-}$ cells DMSO or cisplatin-treated. Scale bar: 50 μm. (**C**) mRNA expression levels of SASP markers in ID8 *Trp53*$^{-/-}$ cells DMSO or cisplatin-treated measured by qRT-PCR. Data represents mean ± SEM (n = 3). *p<0.05; **p<0.01; ns, not significant; two-way ANOVA (Šídák's multiple comparisons test). (**D**) GSEA enrichment plots of OIS and SASP signature in DMSO or cisplatin-treated ID8 *Trp53*$^{-/-}$ cells. (**E**) Heatmap of RNA-Seq data showing SASP expression levels in DMSO or cisplatin-treated ID8 *Trp53*$^{-/-}$ cells. (**F**) GSEA plots of OIS, SMARCA2 target gene, and Cytosolic DNA sensing pathway signature in DMSO or cisplatin-treated ID8 *Trp53*$^{-/-}$ omental tumours.

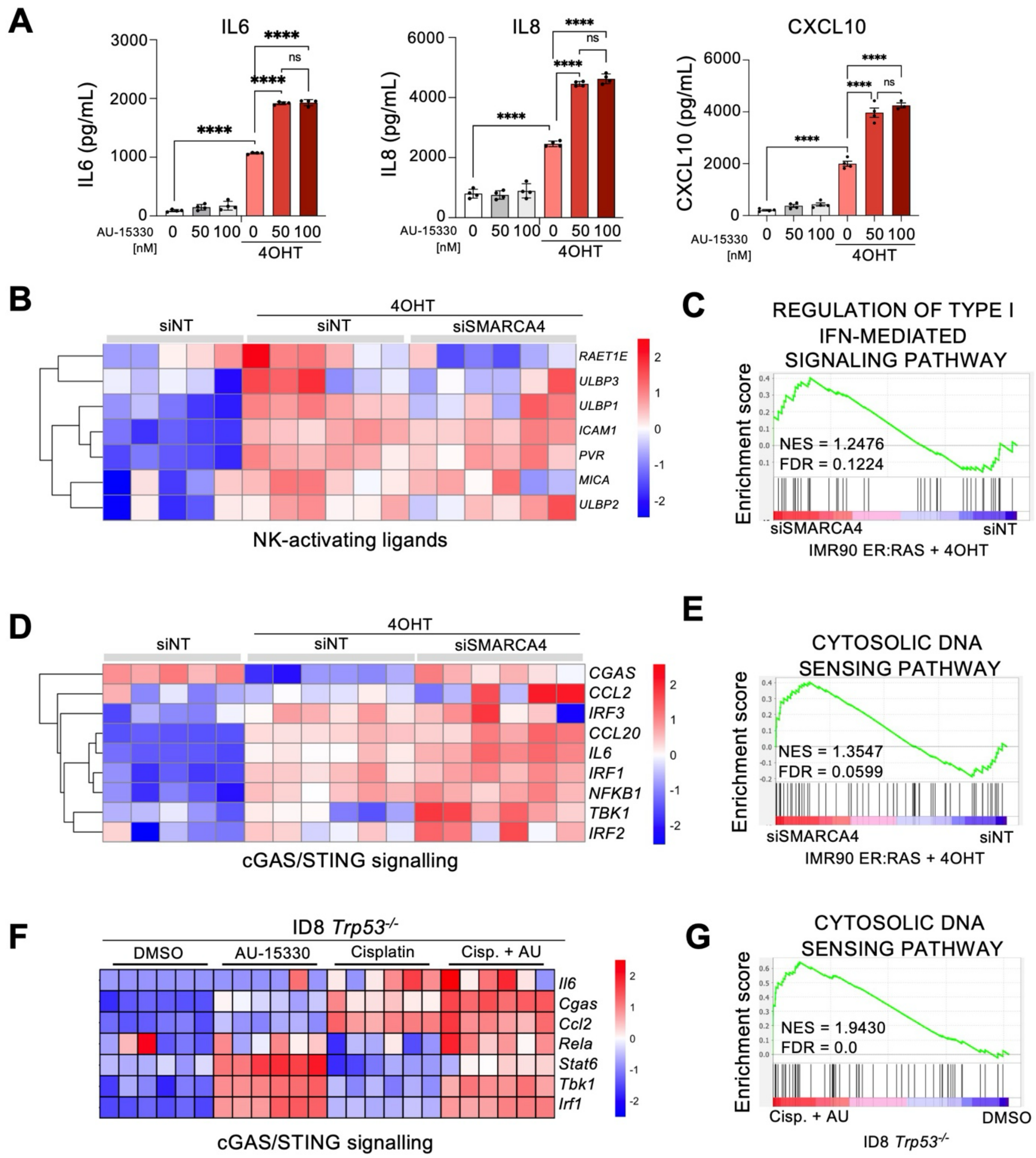

Figure S9. Treatment with AU-15330 activates cGAS/STING signalling. (A) Quantification of the indicated cytokine secreted from IMR90 ER:RAS cells 7 days following DMSO or 4OHT treatment and treated with AU-15330 at day 4, as assessed by ELISA. Data represents mean ± SEM (n = 4). ****$p < 0.0001$; ns, not significant; ordinary one-way ANOVA (Dunnett's multiple comparisons test). (**B**) Heatmap of RNA-Seq data showing the expression of NK-activating ligands in DMSO or 4OHT-treated IMR90 ER:RAS cells and transfected with siNT or siSMARCA4. (**C**) GSEA enrichment plot of

regulation of type I IFN-mediated signalling pathway signature in senescent IMR90 ER:RAS cells transfected with siSMARCA4. (**D**) Heatmap of RNA-Seq data showing the expression of cGAS/STING signalling in DMSO or 4OHT-treated IMR90 ER:RAS cells transfected with siNT or siSMARCA4. (**E**) GSEA enrichment plot of the regulation of Cytosolic DNA sensing pathway signature in senescent IMR90 ER:RAS cells transfected with siSMARCA4. (**F**) Heatmap of RNA-Seq data showing the expression of cGAS/STING signalling in ID8 *Trp53*$^{-/-}$ cells treated with DMSO, AU-15330, cisplatin or cisplatin and AU-15330 combined. (**G**) GSEA plot of the Cytosolic DNA sensing pathway signature in ID8 *Trp53*$^{-/-}$ cells treated with DMSO or cisplatin + AU-15330.

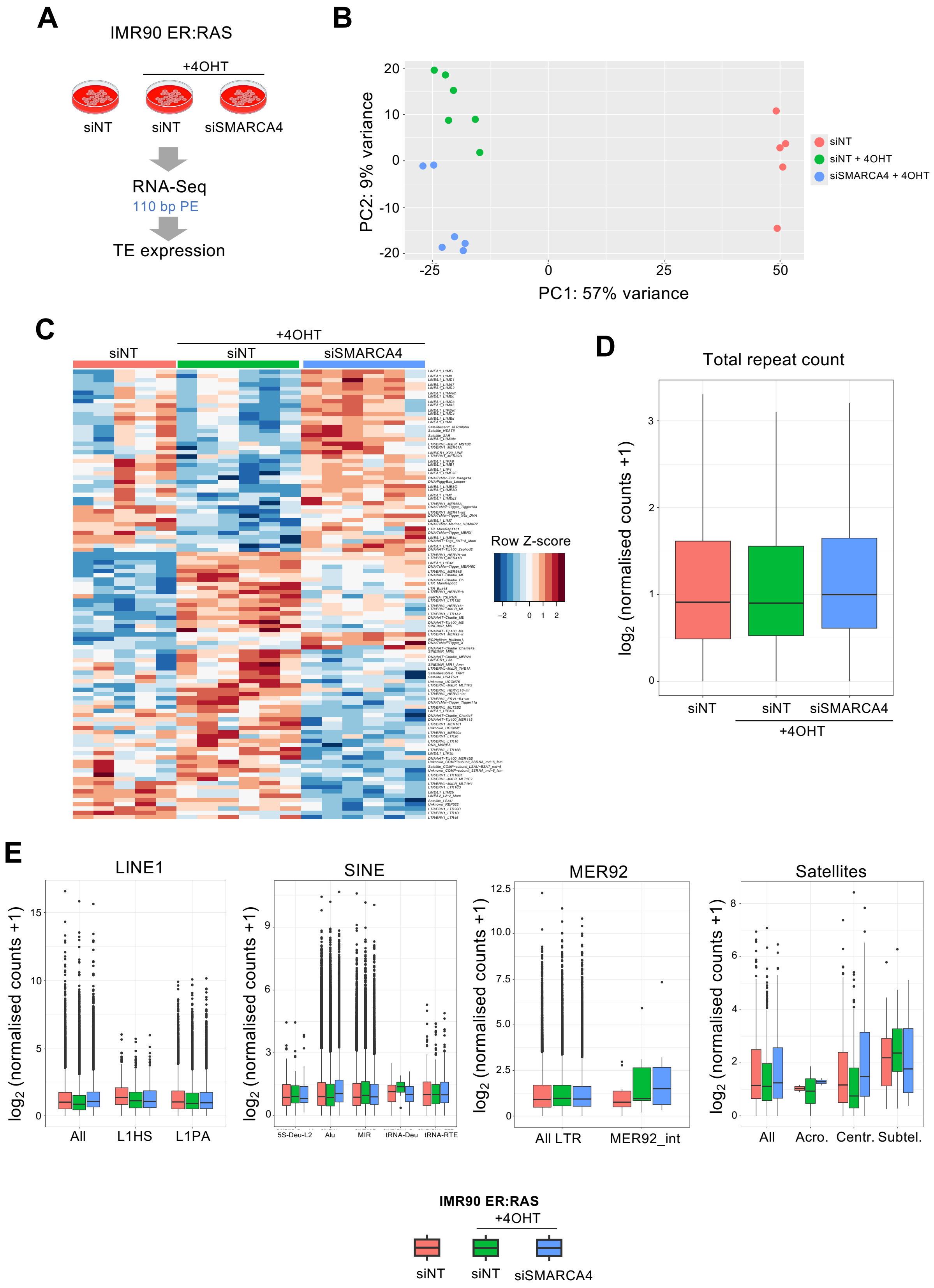
A
IMR90 ER:RAS
+4OHT
siNT
siNT
siSMARCA4
RNA-Seq
110 bp PE
TE expression
B
PC2: 9% variance
PC1: 57% variance
siNT
siNT + 4OHT
siSMARCA4 + 4OHT
C
+4OHT
siNT
siNT
siSMARCA4
Row Z-score
D
Total repeat count
log2 (normalised counts +1)
siNT
siNT
siSMARCA4
+4OHT
E
LINE1
SINE
MER92
Satellites
All
L1HS
L1PA
5S-Deu-L2
Alu
MIR
tRNA-Deu
tRNA-RTE
All LTR
MER92_int
All
Acro.
Centr.
Subtel.
IMR90 ER:RAS
+4OHT
siNT
siNT
siSMARCA4

Figure S10. Effect of SMARCA4 knockdown on the expression of repeat and transposable elements in senescent cells. (A) Scheme showing the set-up of the RNA-Seq experiment to assess transposable and repeat element expression upon SMARCA4 knockdown. **(B)** Principal Components Analysis (PCA) plot of expression at repetitive element loci in IMR90 ER:RAS cells treated with DMSO or 4OHT and siNT or siSMARCA4. PC1 and PC2 are shown. **(C)** Heatmap showing expression of repetitive element subfamilies in IMR90 ER:RAS cells treated with DMSO or 4OHT and siNT or siSMARCA4. Subfamilies shown were those significantly different between siSMARCA4 + 4OHT vs siNT + 4OHT (DESeq2, p adj <0.05). **(D)** Boxplots of normalised expression (DESeq2 median of ratios) of all individual repetitive element loci expression in IMR90 ER:RAS cells treated with DMSO or 4OHT and siNT or siSMARCA4. Boxplot shows the median, first and third quartiles. Whiskers show 1.5 times the interquartile range. Outliers are not displayed. **(E)** Boxplots of normalised expression (DESeq2 median of ratios) of individual repetitive element loci of the indicated families in IMR90 ER:RAS cells treated with DMSO or 4OHT and siNT or siSMARCA4. Boxplot shows the median, first and third quartiles. Whiskers show 1.5 times the interquartile range and dots show outliers.

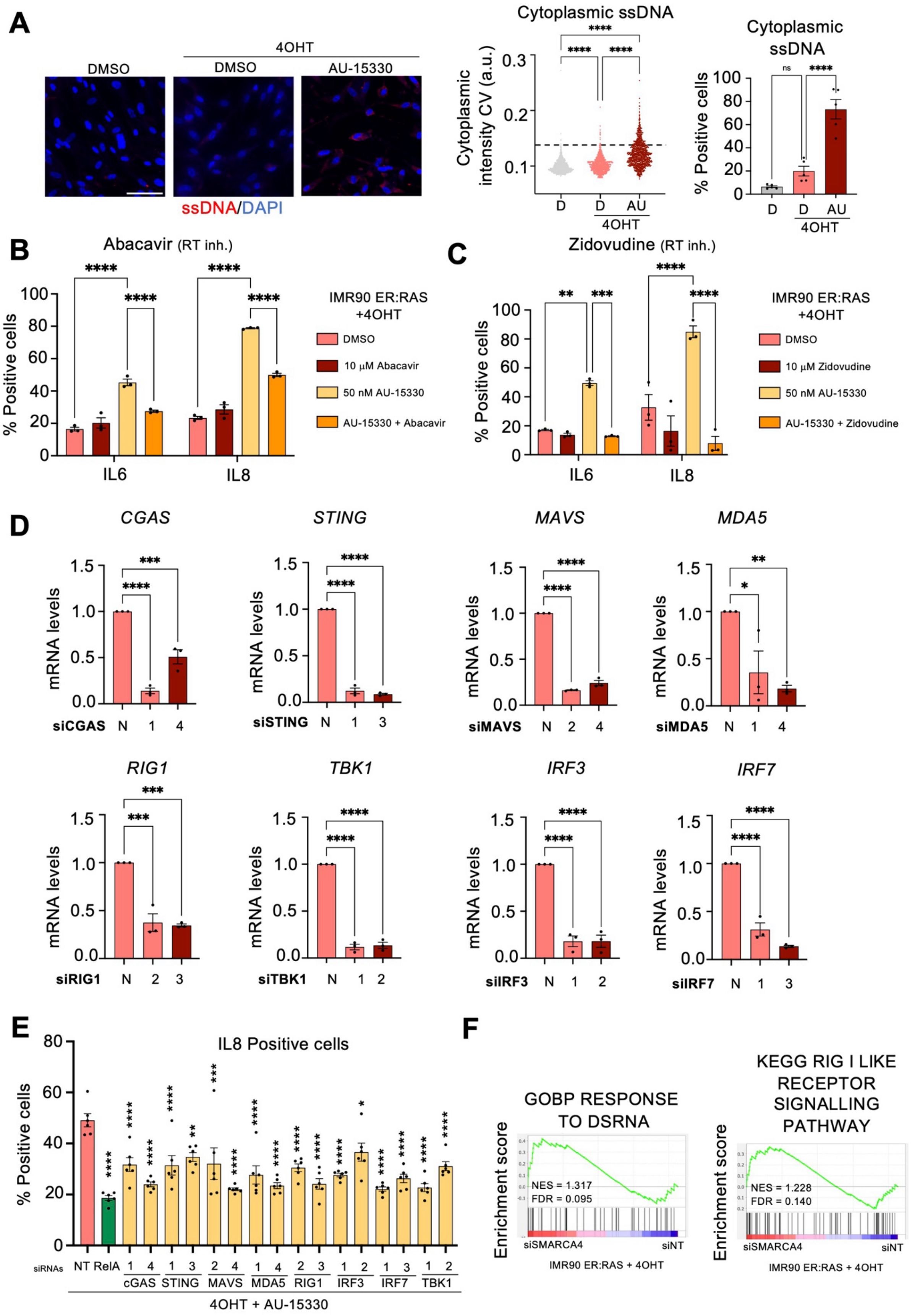
A
DMSO
4OHT
DMSO
AU-15330
ssDNA/DAPI
Cytoplasmic ssDNA
Cytoplasmic intensity CV (a.u.)
D
D AU
4OHT
Cytoplasmic ssDNA
% Positive cells
ns
B
Abacavir (RT inh.)
IMR90 ER:RAS +4OHT
DMSO
10 μM Abacavir
50 nM AU-15330
AU-15330 + Abacavir
IL6
IL8
C
Zidovudine (RT inh.)
10 μM Zidovudine
AU-15330 + Zidovudine
D
CGAS
STING
MAVS
MDA5
RIG1
TBK1
IRF3
IRF7
mRNA levels
siCGAS
siSTING
siMAVS
siMDA5
siRIG1
siTBK1
siIRF3
siIRF7
E
IL8 Positive cells
siRNAs
NT RelA
cGAS STING MAVS MDA5 RIG1 IRF3 IRF7 TBK1
4OHT + AU-15330
F
GOBP RESPONSE TO DSRNA
Enrichment score
NES = 1.317
FDR = 0.095
siSMARCA4
siNT
IMR90 ER:RAS + 4OHT
KEGG RIG I LIKE RECEPTOR SIGNALLING PATHWAY
NES = 1.228
FDR = 0.140

Figure S11. Mechanisms explaining SASP induction upon AU-15330 treatment. (A) Left panel, representative IF images of cytoplasmic ssDNA staining in IMR90 ER:RAS cells with the indicated treatments; Middle panel, quantification of cytoplasmic ssDNA intensity; Right panel, quantification of the percentage of cells positive for cytoplasmic ssDNA in IMR90 ER:RAS cells with and without AU-15330 treatment. ****$p < 0.0001$; ns, not significant; ordinary one-way ANOVA (Dunnett's multiple comparisons test). **(B-C)** Quantification of the percentage of IL6 and IL8 4OHT-induced IMR90 ER:RAS cells in the presence or absence of AU-15330 in combination with Abacavir **(B)** or Zidovudine **(C)** treatment. Data represents mean ± SEM (n = 3). **$p < 0.01$; ***$p < 0.001$; ****$p < 0.0001$; two-way ANOVA (Tukey's multiple comparisons test). **(D)** qRT-PCR analysis showing the mRNA expression of the indicated markers for the MAVS/RIG-1 pathway in IMR90 ER:RAS cells 3 days after being transfected with two selected siRNAs against the indicated genes. Data represents mean ± SEM (n = 3). *$p < 0.05$; **$p < 0.01$; ***$p <0.001$; ****$p <0.0001$; ordinary one-way ANOVA (Dunnett's multiple comparisons test). **(E)** Quantification of the percentage of IL8 in 4OHT-induced IMR90 ER:RAS cells treated with AU-15330 following transfection with the indicated siRNAs. Data represents mean ± SEM (n = 6). *$p < 0.05$; ****$p < 0.0001$; two-way ANOVA (Šídák's multiple comparisons test). **(F)** GSEA enrichment plots of GOBP response to dsRNA and KEGG RIG I-like receptor signalling pathway signature in siSMRCA4 vs siNT transfected IMR90 ER:RAS senescent cells.

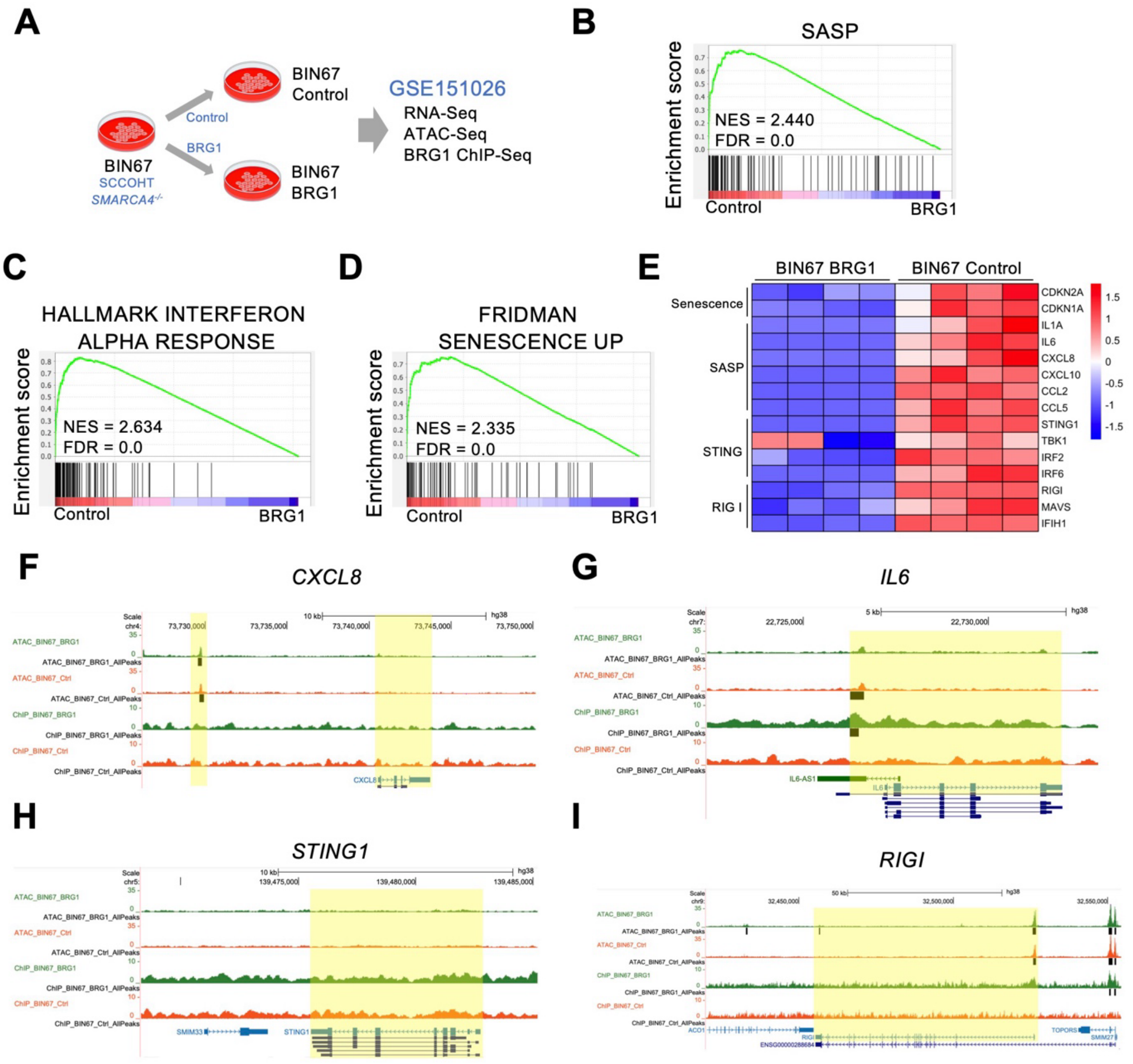

J

Motif	Name	p-value
	Fra1(bZIP)/BT549-Fra1-ChIP-Seq(GSE46166)/Homer	1e-1704
	Fra2(bZIP)/Striatum-Fra2-ChIP-Seq(GSE43429)/Homer	1e-1696
	JunB(bZIP)/DendriticCells-Junb-ChIP-Seq(GSE36099)/Homer	1e-1623
	Atf3(bZIP)/GBM-ATF3-ChIP-Seq(GSE33912)/Homer	1e-1619
	BATF(bZIP)/Th17-BATF-ChIP-Seq(GSE39756)/Homer	1e-1588
	Fosl2(bZIP)/3T3L1-Fosl2-ChIP-Seq(GSE56872)/Homer	1e-1555
	AP-1(bZIP)/ThioMac-PU.1-ChIP-Seq(GSE21512)/Homer	1e-1509
	Jun-AP1(bZIP)/K562-cJun-ChIP-Seq(GSE31477)/Homer	1e-1384
	Bach2(bZIP)/OCILy7-Bach2-ChIP-Seq(GSE44420)/Homer	1e-697
	NF-E2(bZIP)/K562-NFE2-ChIP-Seq(GSE31477)/Homer	1e-271

Figure S12. Effect of BRG1/SMARCA4 re-expression in BIN67 cells. (A) GSE151026 dataset (*49*) investigating re-expression of SMARCA4/BRG1 in the Small Cell Carcinoma of Ovary, Hypercalcemic Type (SCCOHT) cell line BIN67. **(B-D)** GSEA enrichment plots of SASP **(B)**, Interferon alpha **(C)** and Senescence **(D)** signatures in control (*SMARCA4* KO) and BIN67 cells re-expressing SMARCA4/BRG1. Data was reanalysed from GSE151026 dataset (*49*). **(E)** Heatmap derived of the RNA-Seq data showing the expression of selected genes involved in senescence, SASP, STING pathway, and RIGI pathway in control (SMARCA4 KO) and BIN67 cells re-expressing SMARCA4/BRG1. Data was reanalysed from GSE151026 dataset (*49*). **(F-I)** UCSC genome browser tracks of ATAC-seq and BRG1/SMARCA4 ChIP-seq data in BIN67 cells (BIN67_Ctrl) and BIN67 cells with re-expressed BRG1/SMARCA4 (BIN67_BRG1) at the **(F)** *CXCL8*, **(G)** *IL6*, **(H)** *STING1*, and **(I)** *RIGI* loci, which are associated with oncogene-induced senescence. ATAC-seq peak bed files are displayed below the corresponding tracks, and regions of interest are highlighted in yellow. **(J)** Top DNA motifs (known motifs) detected by HOMER v4.11 ($p < 0.05$) for ATAC-seq peaks gained upon BRG1/SMARCA4 re-expression. ChIP-seq, ATAC-seq, and RNA-seq data were obtained from GSE151026 (*49*).

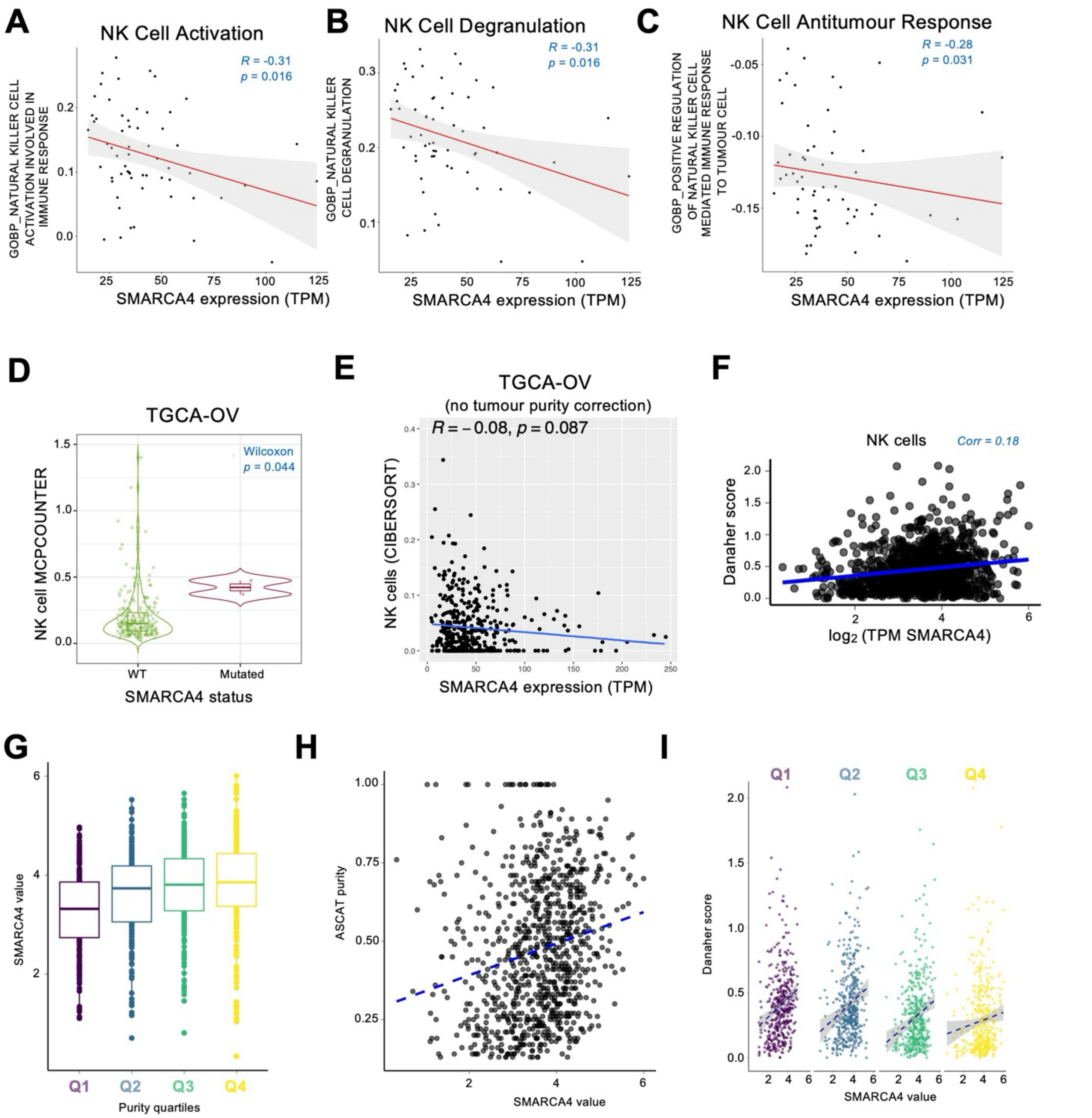

Figure S13. Correlation between SMARCA4 levels and infiltration of NK cells in ovarian and lung cancer patients. (**A-C**) Correlation in the OCTIPS cohort. (**A**) Spearman's correlation between the score for the Gene Ontology Biological Pathway (GOBP) "natural killer cell activation involved in immune response" geneset, and expression of *SMARCA4* in TPM. (**B**) Spearman's correlation between the score for the GOBP "natural killer cell degranulation" geneset, and expression of *SMARCA4* in TPM. (**C**) Spearman's correlation between the score for the GOBP "positive regulation of natural killer cell-mediated immune response to tumour cell" geneset, and expression of

SMARCA4 in TPM. GOBP geneset scores for OCTIPS samples are calculated by singscore (*78*). R = Spearman r, p = p-value. The linear regression line is in red, with grey-shaded areas representing confidence intervals. (**D**) Violin plot illustrating the infiltration levels of NK cells, as estimated by MCPcounter, in ovarian serous adenocarcinoma samples (n = 411) from The Cancer Genome Atlas (TCGA-OV) dataset, using TIMER2.0 (http://timer.cistrome.org). Samples are categorised based on SMARCA4 mutation status, with 407 samples displaying wild-type (WT) SMARCA4 and 4 samples harbouring SMARCA4 mutations. p = 0.044, Wilcoxon rank-sum test, by TIMER2.0. (**E**) Spearman's correlation between absolute NK cell infiltration as inferred by CIBERSORTx (*51*) and expression of *SMARCA4* in transcripts per million (TPM) in the TCGA-OV cohort (n=422) before correction for tumour purity. R = Spearman r, p = p-value. (**F**) Scatter-plot showing the Danaher score for NK cells on the y-axis and the expression value of *SMARCA4* on the x-axis. (**G**) Boxplots of the *SMARCA4* values (log_2(TPM_*SMARCA4*)) on the y-axis and purity quartiles on the x-axis for the TRACERx samples. (**H**) Scatter-plot showing the purity calculation for TRACERx samples on the y-axis and the expression value for *SMARCA4* on the x-axis. (**I**) Scatter plot with a fitted linear model showing the Danaher score for NK cells from TRACERx samples on the y-axis and the expression value for *SMARCA4* on the x-axis, faceted by purity quartiles.

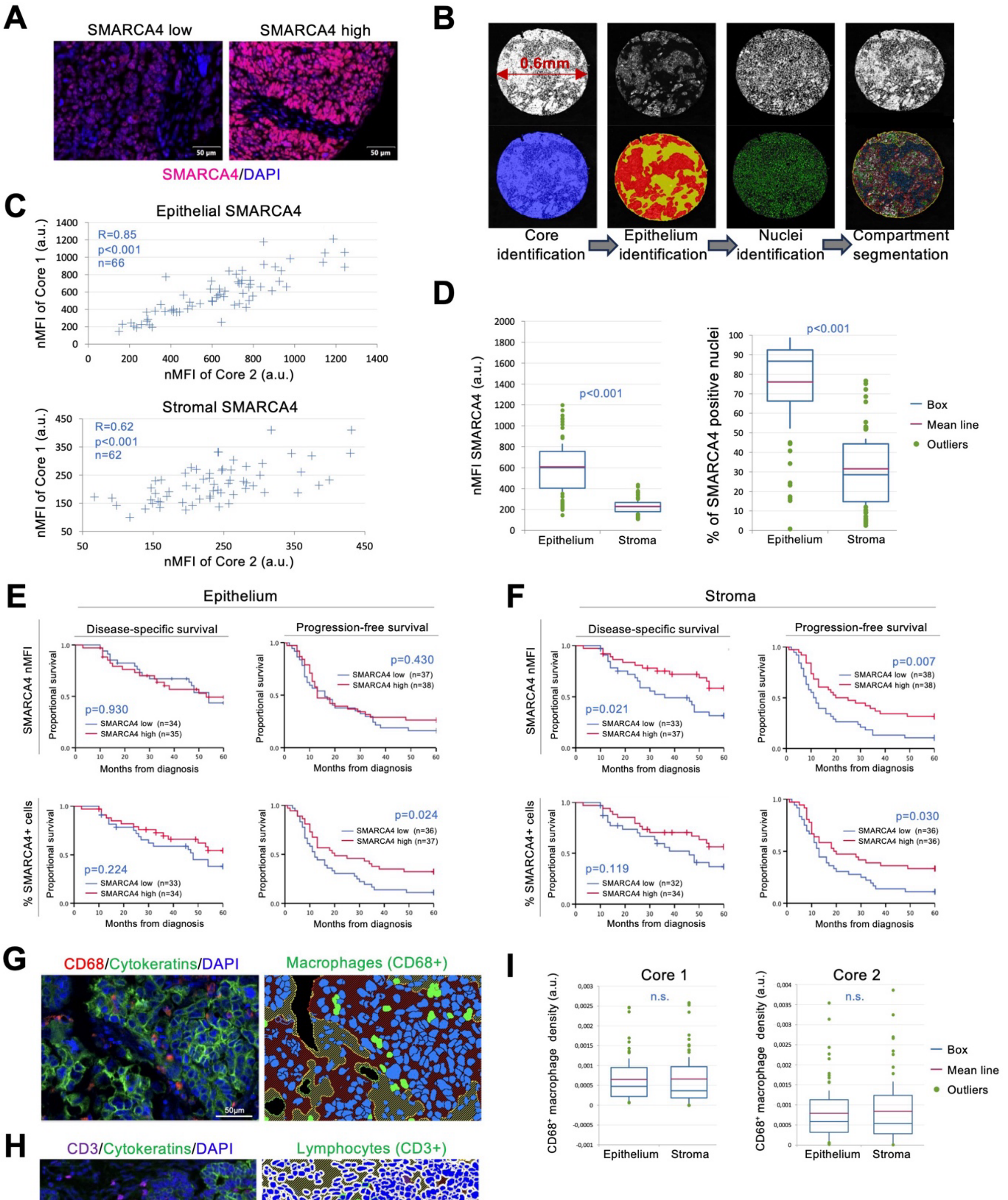

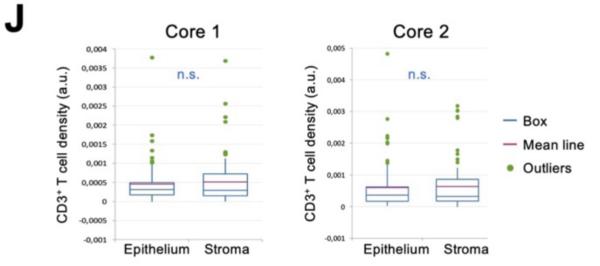

K

Association between immune cell density and SMARCA4 expression levels in all compartments					
Immune cells - Cell count divided by area		Cores 1		Cores 2	
SMARCA4 - nuclear MFI or %cell+		R	*p*	R	*p*
**SMARCA4% - CD68+**	epithelial	0,00247	*0,98402*	0,13813	*0,25770*
**SMARCA4MFI - CD68+**	epithelial	-0,30930	***0,01444***	0,19606	*0,11753*
**SMARCA4% - CD68+**	stromal	0,12680	*0,29916*	-0,00397	*0,97554*
**SMARCA4MFI - CD68+**	stromal	-0,16776	*0,17816*	-0,10769	*0,42951*
**SMARCA4% - CD3+**	epithelial	0,02760	*0,82319*	0,04026	*0,74260*
**SMARCA4MFI - CD3+**	epithelial	-0,13934	*0,28008*	-0,03611	*0,77174*
**SMARCA4% - CD3+**	stromal	0,09453	*0,43296*	-0,12277	*0,34179*
**SMARCA4MFI - CD3+**	stromal	-0,05537	*0,65136*	0,11142	*0,39263*

Figure S14. Single-cell analysis of SMARCA4 in ovarian cancer tissues and association with clinical outcome and immune cells. (**A**) Representative multiplex immunofluorescence images of cores with low (left) or high (right) SMARCA4 expression. (**B**) The pipeline of core tissue identification, segmentation in the epithelium (red) and stroma (yellow), nuclei identification (green), and compartment assignation. Core diameter is 0.6 mm. (**C**) Correlation of nuclear SMARCA4 mean fluorescence intensity (nMFI) in the epithelium (top) and stroma (bottom) between the first core (Y-axis) and second core (X-axis) for each patient. (**D**) Nuclear MFI (left) and percentage of SMARCA4 positive nuclei (right) in the epithelium and stroma. (**E-F**) Percentile 50 Kaplan-Meier analysis of disease-specific survival and progression-free survival based on the SMARCA4 nMFI and percentage of SMARCA4+ cells in the epithelium (**E**) or the stroma (**F**). (**G-H**) Detection of CD68+ macrophages (**G**) or CD3+ lymphocytes (**H**). Left, representative immunofluorescence image; right, same image processed for cell detection (hatched yellow, stroma; hatched red, epithelium; green label, immune cells). (**I-J**) CD68+ macrophage (**I**) or CD3+ lymphocyte (**J**) density analysis in the epithelial and stromal compartment in the first (left) or the second (right) tissue core of each patient. (**K**) Associations between immune cell density and SMARCA4 expression levels in all compartments. Pearson's linear correlation was calculated in (**C**) and (**K**). Mann–Whitney U test was used to calculate significance in (**D**), (**I**) and (**J**). n.s., non-significant.

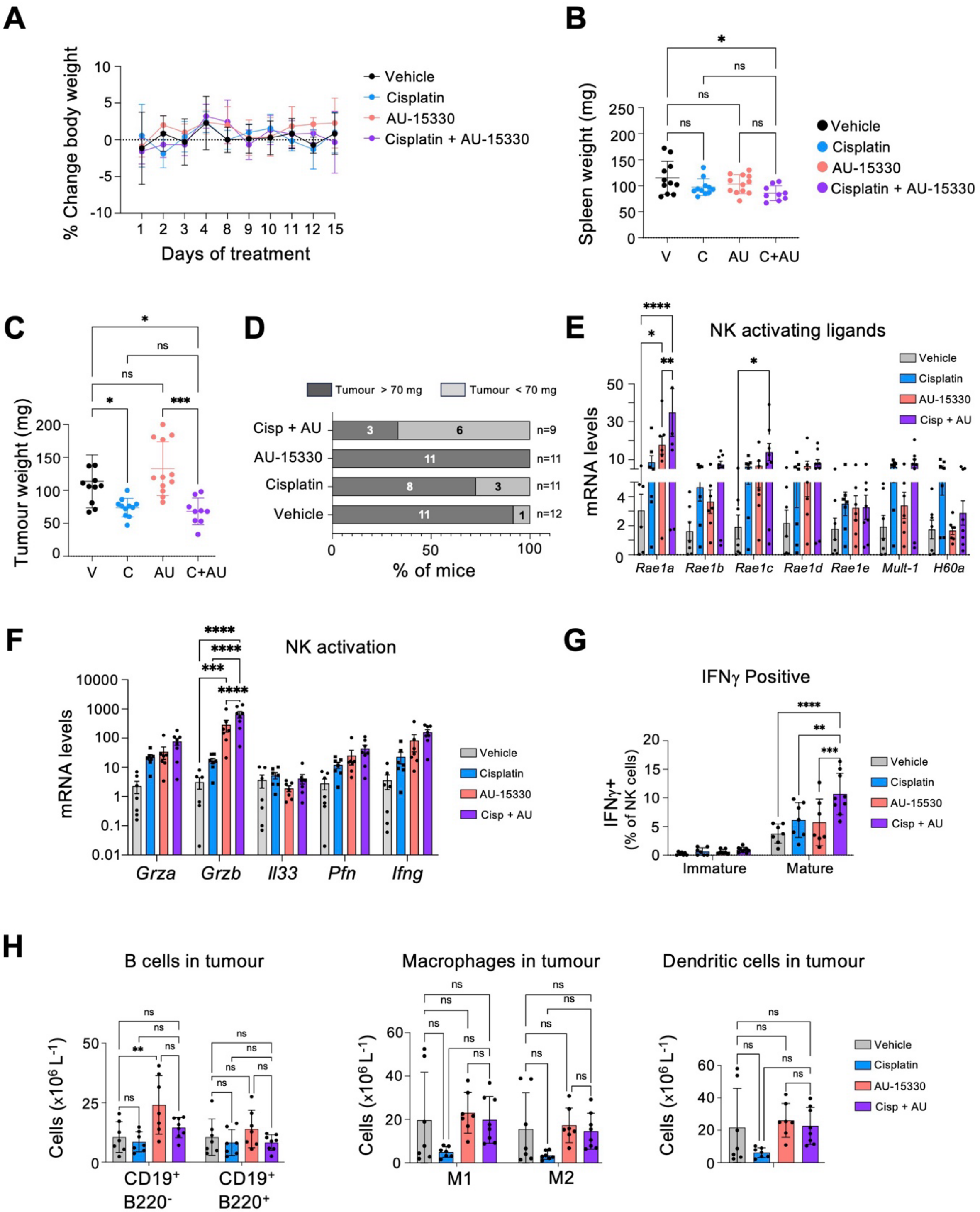

Figure S15. Effects of combined cisplatin and intraperitoneal AU-15330 treatment in a mouse model of ovarian cancer. (**A**) Change in mice body weight (%) during drug administration. n = 7 mice for V, C and AU groups; n = 9 for C+AU. (**B**) Spleen weight (mg) of mice treated with vehicle (V), cisplatin (C), AU-15330 (AU) and cisplatin in

combination with AU-15330 (C+AU). Data represents mean ± SD. *p<0.05, ns, not significant; two-way ANOVA (Tukey's multiple comparisons test). (**C**) Tumour weight (mg) of mice treated with vehicle (V), cisplatin (C), AU-15330 (AU) and cisplatin in combination with AU-15330 (C+AU). Data represents mean ± SD. *p<0.05; ***p<0.001; ns, not significant; two-way ANOVA (Tukey's multiple comparisons test). (**D**) Quantification of the percentage of mice with a tumour weight below 70 mg in the indicated experimental group from the experiment shown in (**C**). For **B-D**, n = 11 mice for V and C; n = 12 mice for AU; n =9 for C+AU. (**E-F**) mRNA expression levels of markers for NK activating ligands (**E**) and immune activation (**F**) in omental tumours measured by qRT-PCR. Data represents mean ± SEM. *p<0.05; **p<0.01; ***p<0.001; ****p<0.0001; two-way ANOVA (Tukey's multiple comparisons test). (**G**) Levels of IFNγ secretion measured by flow cytometry in omental tumours. Data represents mean ± SD. **p<0.01; ***p<0.001; ****p<0.0001; two-way ANOVA (Tukey's multiple comparisons test). (**H**) Flow cytometry analysis of B cells, Macrophages and Dendritic cell counts in omental tumours. Data represents mean ± SD. **p<0.01; ns, not significant; two-way ANOVA (Šídák's multiple comparisons test). For **E-H,** n = 7 mice for V, C and AU groups; n = 8 for C+AU.

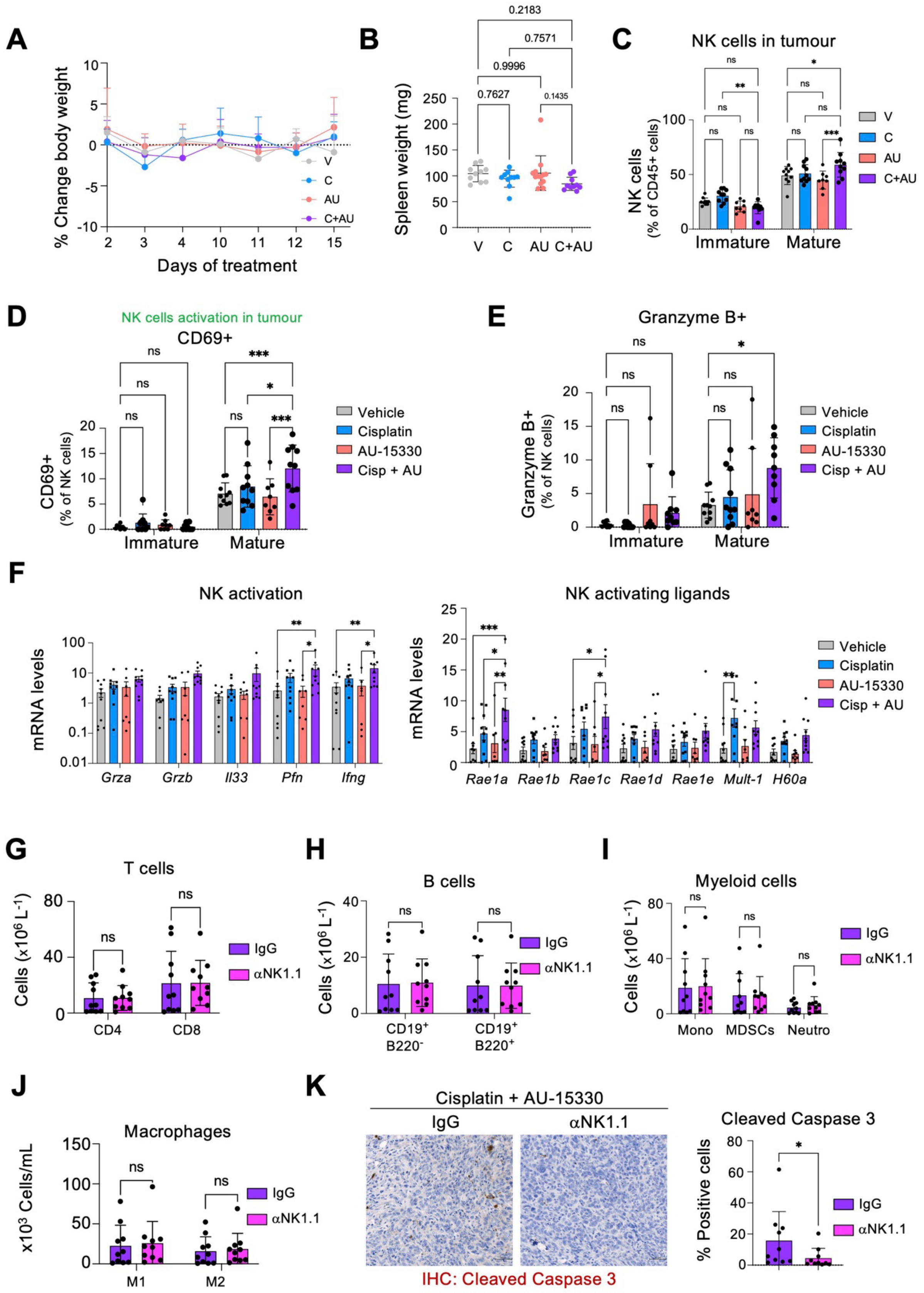
A
% Change body weight
Days of treatment
V
C
AU
C+AU
B
Spleen weight (mg)
0.2183
0.7571
0.9996
0.7627
0.1435
V C AU C+AU
C
NK cells in tumour
NK cells (% of CD45+ cells)
Immature
Mature
D
NK cells activation in tumour
CD69+
CD69+ (% of NK cells)
Vehicle
Cisplatin
AU-15330
Cisp + AU
E
Granzyme B+
Granzyme B+ (% of NK cells)
F
NK activation
mRNA levels
Grza Grzb Il33 Pfn Ifng
NK activating ligands
Rae1a Rae1b Rae1c Rae1d Rae1e Mult-1 H60a
G
T cells
Cells (x10^6 L^-1)
CD4 CD8
IgG
αNK1.1
H
B cells
CD19+ B220-
CD19+ B220+
I
Myeloid cells
Mono MDSCs Neutro
J
Macrophages
x10^3 Cells/mL
M1 M2
K
Cisplatin + AU-15330
IgG
αNK1.1
IHC: Cleaved Caspase 3
Cleaved Caspase 3
% Positive cells

Figure S16. Effects of combined cisplatin and intravenous AU-15330 treatment in a mouse model of ovarian cancer. (**A**) Change in mice body weight (%) during drug administration. (**B**) Spleen weight (mg) of mice treated with vehicle (V), cisplatin (C), AU-15330 (AU) and cisplatin in combination with AU-15330 (C+AU). Data represents mean ± SD. One-way ANOVA (Tukey's multiple comparisons test). For **A-B**, n = 10 mice for V, C and C+AU groups; n = 13 for AU. (**C**) Flow cytometry analysis of the percentage of NK cells in the omental tumours. Data represents mean ± SD. *p<0.05; **p<0.01; ***p<0.001; ns, not significant; two-way ANOVA (Tukey's multiple comparisons test). (**D**) Flow cytometry analysis of NK cell activation (% of CD69+ NK cells) in omental tumours. Data represents mean ± SD. *p<0.05; ***p<0.001; ns, not significant; two-way ANOVA (Tukey's multiple comparisons test). (**E**) Levels of Granzyme B secretion measured by flow cytometry in omental tumours. Data represents mean ± SD. *p<0.05; ns, not significant; two-way ANOVA (Tukey's multiple comparisons test). **F**) mRNA expression levels of markers of immune activation in omental tumours and NK activating ligands measured by qRT-PCR. Data represents mean ± SD. *p<0.05; **p<0.01; two-way ANOVA (Tukey's multiple comparisons test). For **C-F**, n = 10 mice for V, C and C+AU groups; n = 8 for AU. (**G-J**) Flow cytometry analysis of T cell count (**G**), B cell count (**H**), myeloid cell count (**I**) and macrophages count (**J**) in omental tumours from mice treated with C+AU+IgG and C+AU+αNK1.1. Data represents mean ± SD. ns, not significant; two-way ANOVA (Šídák's multiple comparisons test). (**K**) Representative images (left) and quantification (right) of Cleaved-Caspase 3 staining in omental tumours treated with C+AU+IgG or C+AU+αNK1.1. Scale bar, 50 μm. Data represents mean ± SD. *p < 0.05; two-way ANOVA (Tukey's multiple comparisons test). For **G-K,** n = 10 mice for C+AU+IgG and C+AU+ αNK1.1 groups.

SUPPLEMENTARY TABLES

Table S1. Candidates identified in the primary SASP. Related to Fig. 1, it is included as an Excel file.

Table S2. Hits of the screens for siRNAs superinducing SASP. Related to Fig. 1, it is included as an Excel file.

Table S3. Hits of the screen for siRNAs enhancing NK-mediated killing of senescent cells. Related to Fig. 2, it is included as an Excel file.

Table S4. Chemical compounds.

**Chemical compounds**	**CAS Number**	**Supplier**	**Catalogue number**
4-OHT	68392-35-8	Sigma-Aldrich	H7904
SGC-SMARCA-BRDVIII	1997319-84-2	Sigma-Aldrich	SML3094
Etoposide	33419-42-0	Sigma-Aldrich	E1383
Indisulam	165668-41-7	Sigma-Aldrich	SML1225
AU-15330	2380274-50-8	MedChemExpress	HY-145388
Cisplatin	15663-27-1	APExBIO	A8321
G150	2369751-30-2	Selleckchem	S8944
C171	2244881-69-2	Selleckchem	E0128
C176	314054-00-7	MedChemExpress	HY-112906
Lamivudine (3TC)	134678-17-4	MedChemExpress	HY-B0250
Abacavir	136470-78-5	MedChemExpress	HY-17423
Zidovudine	30516-87-1	MedChemExpress	HY-17413

Table S5. Primary antibodies used for IF and IHC.

Target	Clone	Manufacturer	Catalogue number	H/M*	Dilution	RRID
**53BP1**	Polyclonal	Novus Biologicals	NB100-304	H	1:1000	AB_350221
**γ-H2AX**	JWB301	Millipore	05-636	H, M	1:100	AB_2755003
**BrdU**	3D4	BD Biosciences	555627	H	1:1000	AB_395993
**CCL2**	Polyclonal	R&D Systems	AF-279-NA	H	1:250	AB_354441
**IL1α**	4414	R&D Systems	MAB-200	H	1:250	AB_2295862
**IL1β**	8516	R&D Systems	MAB-201	H	1:250	AB_358006
**IL6**	Polyclonal	R&D Systems	AF-206-NA	H	1:250	AB_354392
**IL8/CXCL8**	6217	R&D Systems	MAB-208	H	1:250	AB_2249110
**$p16^{INK4a}$**	JC8	CRUK	-	H	1:1000	
**$p21^{CIP1}$**	12D1	Cell Signaling Technology	2947	H	1:2000	AB_823586
**BRG1 (SMARCA4)**	EPNCIR111A	Abcam	Ab110641	H, M	1:500	AB_10861578
**$p21^{CIP1}$**	EPR18021	Abcam	Ab188224	M	1:500	AB_2734729
**CXCL10**	10H11L3	Thermo Fisher Scientific	701225	H, M	1:500	AB_2532429
**KI67**	SP6	Thermo Fisher Scientific	MA5-14520	H, M	1:500	AB_10979488

**N-RAS**	F155	Santa Cruz Technology	Sc-31	M	1:500	AB_628041
**ORF1p**	4H1	Sigma Aldrich	MABC1152	H	1:250	AB_2941775
**cGAS**	D-9	Santa Cruz Technology	sc-515777	H	1:250	AB_2734736
**pSTING**	D8K6H	Cell Signaling Technology	40818	H, M	1:800	AB_2799187
**pTBK1**	D52C2	Cell Signaling Technology	5483s	H, M	1:100	AB_10693472
**pIRF3**	4D4G	Cell Signaling Technology	4947s	H, M	1:500	AB_823547
**ssDNA**	TNT-3	Sigma Aldrich	MAB3868	H, M	1:100	AB_570342
**dsDNA**	rDSD/4565	Novus Biologicals	NBP3-07670	H, M	1:100	AB_3107112
**dsRNA**	rJ2	Sigma Aldrich	MABE1134	H, M	1:100	AB_2819101
**CD68**	PG-M	Dako	M0876	H	1:50	AB_2074844
**CD3**	CD3-12	Abcam	Ab11089	H, M	1:200	AB_2889189
**Cytokeratin 7**	OV-TL12/30	Novus	NBP2-44814	H, M	1:200	-
**Cytokeratin 18**	DC-10	Santa Cruz	sc-6259	H	1:200	AB_627850
**Cytokeratin 19**	A53-B/A2.26	Novus	NBP2-15186	H	1:200	-

*Reactivity: H, human; M, mouse.

Table S6. Antibodies used for flow cytometry analysis.

Antigen	Fluorophore	Company	Clone	Catalogue number	RRID
CD45	BV605	BioLegend	30-F11	103140	AB_2650656
CD3	FITC	BioLegend	17A2	100204	AB_312660
CD19	Pe-Cy7	Invitrogen	1D3	25-0193-82	AB_657663
NK1.1	PE	BioLegend	PK136	108707	AB_313394
CD4	APC-Cy7	BioLegend	GK1.5	100414	AB_312698
CD49b	APC	BioLegend	DX5	108909	AB_313416
B220	BV711	BioLegend	RA3-6B2	103255	AB_2563491
CD8	BV650	BioLegend	53-6.7	100741	AB_2563056
CD11b	BV421	BioLegend	M1/70	101251	AB_11203704
CD11c	PerCP-Cy5.5	BioLegend	N418	117327	AB_2129642
F4/80	PE	BioLegend	BM8	123110	AB_893486
MHCII	BV510	BioLegend	M5/114.15.2	107635	AB_2561397
CD206	APC	BioLegend	C068C2	141707	AB_10896057
LY6C	FITC	BioLegend	HK1.4	128005	AB_1186134
LY6G	APC-Cy7	BioLegend	1A8	127623	AB_10645331
CD107a	FITC	BioLegend	1D4B	121605	AB_572006
CD69	PerCP	BioLegend	H1.2F3	104520	AB_940495
NKG2D	BV711	BD	CX5	563694	AB_2722498
NKp46	BV650	BioLegend	29A1.4	137635	AB_2734200
IFN-γ	PerCP-Cy5.5	BioLegend	XMG1.2	505821	AB_961359
Granzyme B	Pacific Blue	BioLegend	GB11	515407	AB_2562196

Table S7. Secondary antibodies used in this study.

Target	Clone / Conjugate	Manufacturer	Catalogue number	Dilution	RRID
**Goat IgG (H+L)**	Polyclonal / AlexaFluor® 594	Invitrogen	A-11058	1:2000	AB_2534105
**Goat IgG (H+L)**	Polyclonal / AlexaFluor® 488	Invitrogen	A-11055	1:2000	AB_2534102
**Mouse IgG (H+L)**	Polyclonal / AlexaFluor® 594	Invitrogen	A-11032	1:2000	AB_2534091
**Mouse IgG (H+L)**	Polyclonal / AlexaFluor® 488	Invitrogen	A-11059	1:2000	AB_2534106
**Rabbit IgG (H+L)**	Polyclonal / AlexaFluor® 594	Invitrogen	A-11037	1:2000	AB_2534095
**Rabbit IgG (H+L)**	Polyclonal / AlexaFluor® 488	Invitrogen	A-11008	1:2000	AB_143165
**Rabbit IgG**	-	Cell Signaling	8114S	-	AB_10544930
**Mouse IgG**	-	Cell Signaling	8125S	-	AB_10547893
**Mouse IgG (H+L)**	Polyclonal / Alexa-Fluor® 647	Invitrogen	A-31571	1:250	AB_162542

**Rat IgG (H+L)**	Polyclonal / Alexa Fluor® 750	Invitrogen	Ab175750	1:250	-
**Rabbit IgG (H+L)**	Polyclonal / Alexa-Fluor® 647	Invitrogen	A-31573	1:250	AB_2536183
**Mouse IgG (H+L)**	Polyclonal / Alexa-Fluor® 750	Invitrogen	A-21037	1:250	AB_2535708

Table S8. RT-qPCR Primers used to detect human transcripts.

Target	Forward primer	Reverse primer
RPS14	CTGCGAGTGCTGTCAGAGG	TCACCGCCCTACACATCAAACT
CDKN1A	CGTGTCACTGTCTTGTACCCT	GCGTTTGGAGTGGTAGAAATCT
CDKN2A	CGGTCGGAGGCCGATCCAG	GCGCCGTGGAGCAGCAGCAGCT
PLAUR	CCACTCAGAGAAGACCAACAGG	GTAACGGCTTCGGGAATAGGTG
LMNB1	GAGAGCAACATGATGCCCAAGTG	GTTCTTCCCTGGCACTGTTGAC
IL1A	AGTGCTGCTGAAGGAGATGCCTGA	CCCCTGCCAAGCACACCCAGTA
IL1B	TGCACGCTCCGGGACTCACA	CATGGAGAACACCACTTGTTGCTCC
IL6	CCAGGAGCCCAGCTATGAAC	CCCAGGGAGAAGGCAACTG
IL8	GAGTGGACCACACTGCGCCA	TCCACAACCCTCTGCACCCAGT
CXCL10	GGTGAGAAGAGATGTCTGAATCC	GTCCATCCTTGGAAGCACTGCA
TNFA	CTCTTCTGCCTGCTGCACTTTG	ATGGGCTACAGGCTTGTCACTC
SMARCA4	CAAAGACAAGCACATCCTCGCC	GCCACATAGTGCGTGTTGAGCA
FURIN	GCCACATGACTACTCCGCAGAT	TACGAGGGTGAACTTGGTCAGC
POLR1B	CACAACCAGAGTCCACGGAACA	TCACCAAGGGACTCTGAGGAGT
IFNG	GAGTGTGGAGACCATCAAGGAAG	TGCTTTGCGTTGGACATTCAAGTC
GZMA	CCACACGCGAAGGTGACCTTAA	CCTGCAACTTGGCACATGGTTC
GZMB	CGACAGTACCATTGAGTTGTGCG	TTCGTCCATAGGAGACAATGCCC
PRF1	ACTCACAGGCAGCCAACTTTGC	CTCTTGAAGTCAGGGTGCAGCG
CGAS	AGGAAGCAACTACGACTAAAGCC	CGATGTGAGAGAAGGATAGCCG
STING	CCTGAGTCTCAGAACAACTGCC	GGTCTTCAAGCTGCCCACAGTA
MAVS	ATGGTGCTCACCAAGGTGTCTG	TCTCAGAGCTGCTGTCTAGCCA
MDA5	GCTGAAGTAGGAGTCAAAGCCC	CCACTGTGGTAGCGATAAGCAG
RIG-1	CACCTCAGTTGCTGATGAAGGC	GTCAGAAGGAAGCACTTGCTACC
IRF3	TCTGCCCTCAACCGCAAAGAAG	TACTGCCTCCACCATTGGTGTC
IRF7	CCACGCTATACCATCTACCTGG	GCTGCTATCCAGGGAAGACACA
TBK1	CAACCTGGAAGCGGCAGAGTTA	ACCTGGAGATAATCTGCTGTCGA

Table S9. RT-qPCR Primers used to detect mouse transcripts.

Target	Forward primer	Reverse primer
Rn18S	ACCGCAGCTAGGAATAATG	GCCTCAGTTCCGAAAACCA
Gapdh	AACTTTGGCATTGTGGAAGG	ACACATTGGGGGTAGGAACA
Il6	CCTGAGACTCAAGCAGAAATGG	AGAAGGAAGGTCGGCTTCAGT
Mcp1/ Ccl2	CATCCACGTGTTGGCTCA	GATCATCTTGCTGGTGAATGAGT
Tnfa	GGTGCCTATGTCTCAGCCTCTT	GCCATAGAACTGATGAGAGGGAG
Ccl5	CTGCTGCTTTGCCTACCTCT	CGAGTGACAAACACGACTGC
Cxcl10	ATCATCCCTGCGAGCCTATCCT	GACCTTTTTTGGCTAAACGCTTTC
Cxcl1	CTGGGATTCACCTCAAGAACATC	CAGGGTCAAGGCAAGCCTC
Il15	GTAGGTCTCCCTAAAACAGAGGC	TCCAGGAGAAAGCAGTTCATTGC
Il18	GACAGCCTGTGTTCGAGGATATG	TGTTCTTACAGGAGAGGGTAGAC
Raet1a	GACAGCAAATGCCACTGAAGTGG	GCTTTGCAGATAAATCATGGTGAC
Raet1b	CCAAAGTGGACACTCACAAGACC	GAATTCCCAGGTGGCACTAGGA
Raet1c	GACGGCAAATGCCACTGAAGTG	GCCCTGGCTTTGCGGATAAATC
Raet1d	CCAAAGTGGACACTCACAAGACC	GCTCATAATCTCTGTGTAGAAGGT
Raet1e	CCTCTGAACGATTTGTGCCAGG	GCCCTGGCTTTGCGGATAAATC
Mult-1	GTGCAGGAGACTAACACAACCG	TGCCAGTGCTTGTGTCAACACG
H60a	GGTCTGAGTGTCACCTGGATTG	TTTTCTTCAGCATACACCAAGCGA
GranzymeA	TGCTGCCCACTGTAACGTG	GGTAGGTGAAGGATAGCCACAT
GranzymeB	CCACTCTCGACCCTACATGG	GGCCCCCAAAGTGACATTTATT
Perforin	AGCACAAGTTCGTGCCAGG	GCGTCTCTCATTAGGGAGTTTTT
Il33	ATGGGAAGAAGCTGATGGTG	CCGAGGACTTTTTTGAAGG
Ifng	ATGAACGCTACACACTGCATC	CCATCCTTTTGCCAGTTCCTC

Table S10. siRNA sequences.

Target	siRNA_ID	Sequence	Cat. number
**Non targeting**	2	UAAGGCUAUGAAGAGAUAC	D-001210-02
**Non targeting**	4	AUGAACGUGAAUUGCUCAA	D-001210-04
L3HYPDH	3	GAAGAUGACGACCCAUUGA	D-010373-03
EPRS	3	GAAGAGGGAUGACAGUUGA	D-008245-03
SIRT1	3	GCGAUUGGGUACCGAGAUA	D-003540-07
LGMN	2	GAACAGAUCGUUGUGAUGA	D-005924-02
LPAR2	1	UCUACUACCUGCUCGGCAA	D-004602-21
FOSL1	1	GCUCAUCGCAAGAGUAGCA	D-004341-23
	2	GGACACAGGCAGUACCAGU	D-004341-24
	3	AGCGAGAGAUUGAGGAGCU	D-004341-25
	4	GCAGGCGGAGACUGACAAA	D-004341-26
KDM5C	1	GAGCGGAGGUUUCCUAAUA	D-010097-01
	2	GUGGACAACUUCAGGUUUA	D-010097-02
	3	GCAAGGAUAUGCCUAAGGU	D-010097-03
	4	GAGUGAAACUGAACUACUU	D-010097-04
MMP8	1	GCACUAAUGUGAUCGUUGA	D-005969-01
	2	CCAAGGAUAUAUCAAACUA	D-005969-02
	3	GAGAAGGCAUCCUCAGCUA	D-005969-03
	4	AGAAAGAGCUAUCAAGGAU	D-005969-04
PHF19	1	CCUCGUGACUUUCGAAGAU	D-026200-03
	2	GGAAGUGUGGCCUGGGUUA	D-026200-20
	3	GGUCAGCAGCUCUAAGCAA	D-026200-21
	4	GAUCCAGGGACUCGGGACU	D-026200-22
POLR1B	1	UCAUAGGAGUGGUUUAUUA	D-013391-02
	2	GAACCCAACUAUCGGAGAU	D-013391-03
	3	ACCAAUGCCAGAUGGGUAA	D-013391-17
	4	CCUUAACUACCUAGGUGAA	D-013391-18

SMARCA4	1	GAAAGGAGCUGCCCGAGUA	D-010431-01
	2	CCAAGGAUUUCAAGGAAUA	D-010431-02
	3	GAAAGUGGCUCAGAAGAAG	D-010431-03
	4	AGACAGCCCUCAAUGCUAA	D-010431-04
CD247	1	GGAAUCCUCUUCAUCUAUG	D-004257-01
	2	GAAGAGAGGAGUACGAUGU	D-004257-02
	3	ACAGUGAGAUUGGGAUGAA	D-004257-04
	4	AGGAAGGCCUGUACAAUGA	D-004257-05
DPH5	1	GGAAUUCCUUAUAGAGUUA	D-020926-01
	2	GUAUAUGAGUGUAAACCAA	D-020926-02
	3	ACAGUGAUCUUGUUCUAAG	D-020926-03
	4	ACGCUGCAGUCGAGUGUAU	D-020926-04
FCRL2	1	CCUGAUGGCUAUAGAAGAG	D-016490-01
	2	CCUCAUGACAGCUGGAGUU	D-016490-02
	3	CCCAAUCUUGUACCAAUUU	D-016490-03
	4	GUCCUUGGUUUCACUGGUG	D-016490-04
FOXH1	1	CCACGAGAGUGGAGGGGGA	D-008636-01
	2	AGAAGAGGUACCUGCGACA	D-008636-02
	3	GGAAAGACUCCAUUCGCCA	D-008636-03
	4	CCAAUGUGGUAAUGCCCUU	D-008636-04
FURIN	1	GGACUAAACGGGACGUGUA	D-005882-01
	2	GGACUUGGCAGGCAAUUAU	D-005882-02
	3	GCAGAUGGGUUUAAUGACU	D-005882-03
	4	GGGCUGGGCUCCAUCUUUG	D-005882-04
GLRA4	1	UGAAAGACCUCGUGUUUGA	D-034258-05
	2	GCGAACAUCUGGAUAUGAU	D-034258-06
	3	GACCACGGACAACAAGUUA	D-034258-07
	4	CGCCUGUCCUACCGAGAAU	D-034258-09
ARID1A	1	GCAACGACAUGAUUCCUAU	D-017263-01
	2	GAAUAGGGCCUGAGGGAAA	D-017263-02

	3	AGAUGUGGGUGGACCGUUA	D-017263-03
	4	UAGUAUGGCUGGCAUGAUC	D-017263-04
ARID1B	1	CCACAGCGGUAUCCAAUUG	D-013970-01
	2	GUAUGGACCUCAACAGACA	D-013970-02
	3	GGCGAAAGAUUACCUCCAA	D-013970-03
	4	GGCGUGAGCUGGCAACCAA	D-013970-04
ARID1A	1	GGUCGCAGAUUGUUAAAGA	D-026945-01
	2	CGACAGCGGUUUUCUUUUA	D-026945-02
	3	CCAAAUAAAGUAGGAGUUA	D-026945-03
	4	GCAAUUAGGCCUUGACACA	D-026945-04
ARID1B	1	GGACAUGCAUGACGAUAAC	D-013141-01
	2	GCCCAAGGUUGAUGACAAA	D-013141-02
	3	ACACAUCCCUACGAAUCUA	D-013141-03
	4	GGACGAGAAGUGUGGCUCA	D-013141-04
ARID2	1	GUACUAAUGCCAUGAUUUA	D-020297-01
	2	GCAAGUAACUCCAGGUGAU	D-020297-02
	3	GCACGUAUGGAGUUCGAAA	D-020297-04
	4	GAACAGUCAGUGCGAAUUU	D-020297-17
BCL7A	1	CAAUGAAGAUACAGCUGUU	D-014250-01
	2	GGCCAGAUACCGUGUACUA	D-014250-02
	3	CACGCAGGCUUUAAGAUGA	D-014250-03
	4	AGUCAGUUACGGAAUUUAA	D-014250-04
BRD7	1	CGGGACAGAUUUACACGUA	D-031779-01
	2	CAUUAUGGACUGUCAGAAA	D-031779-03
	3	GGCCAAAGGAAAGGCAUAU	D-031779-04
	4	GCGAGGACUUCUACCGCGA	D-031779-17
BRD9	1	GUGGACAGCUCAUCGCGAA	D-020751-02
	2	GUAAUGAUCGACCGAAUGU	D-020751-03
	3	GCAUCGGGCUCAAGCUCAA	D-020751-04
	4	GAUGCUUACUAGACGUGAU	D-020751-17

DPF1	1	GAAAUCAACCAGACUAUUA	D-008692-01
	2	GAAAUGAGUUUGUUCAGAA	D-008692-02
	3	GAAACUGCCUUCUAAAGUG	D-008692-03
	4	UGACAUGGCUUCUCCCAAA	D-008692-04
GLTSCR1	1	GCACUUCGGUUACUCAAUU	D-017253-01
	2	CCGCAUAGCUCAUAGGAUA	D-017253-02
	3	GAAGGGCACUGGUAUGCGA	D-017253-03
	4	CAAAGCAACCGUGGAACUA	D-017253-04
PBRM1	1	GAAAGGAGCUGCCCGAGUA	D-010431-01
	2	CCAAGGAUUUCAAGGAAUA	D-010431-02
	3	GAAAGUGGCUCAGAAGAAG	D-010431-03
	4	AGACAGCCCUCAAUGCUAA	D-010431-04
SMARCA2	1	GAAACUACCUCCGUAUGUU	D-010536-01
	2	CCACAACCAUCAACAGGAA	D-010536-02
	3	GUGACGAUCUGGAUUUGAA	D-010536-03
	4	AGACCUACGCCUUCAGCGA	D-010536-17
SMARCB1	1	GAAGUUGGCUAACAAAUUG	D-010813-01
	2	GAACUUGACUGGAGAUGUG	D-010813-02
	3	GAAGUAUGCUGAAUUACGA	D-010813-03
	4	GAGCAGACCAAUCACAUUA	D-010813-04
SMARCC1	1	GAUCAAAUGUUUCCUAGAU	D-008312-01
	2	CCAAGACACUGACAGAUGA	D-008312-02
	3	GCUACUAUCCUGACAGUUA	D-008312-03
	4	GAUCAAACUUCGGCACUUU	D-008312-04
SMARCC2	1	UCAGUGCUCUGGACAGUAA	D-009459-01
	2	UAAACCAGCUCAAGAUCCA	D-009459-02
	3	UGACAAGGAAUACAUCAAU	D-009459-03
	4	GAAGAAGACGGCGUGCUAU	D-009459-05
SMARCD3	1	GCAACGACAUGAUUCCUAU	D-017263-01
	2	GAAUAGGGCCUGAGGGAAA	D-017263-02

	3	AGAUGUGGGUGGACCGUUA	D-017263-03
	4	UAGUAUGGCUGGCAUGAUC	D-017263-04
cGAS (MB21D1)	1	GAAGAAACAUGGCGGCUAU	D-015607-01
	2	GAAGAGAAAUGUUGCAGGA	D-015607-02
	3	GUAAGGAAUUUCUGACAAA	D-015607-03
	4	CAACACUCGUGCAUAUUAC	D-015607-04
STING (TMEM173)	1	GCACCUGUGUCCUGGAGUA	D-024333-01
	2	GGUCAUAUUACAUCGGAUA	D-024333-02
	3	GCAUCAAGGAUCGGGUUUA	D-024333-03
	4	ACAUUCGCUUCCUGGAUAA	D-024333-04
MAVS	1	AAGUAUAUCUGCCGCAAUU	D-024237-02
	2	GCUGUGAGCUAGUUGAUCU	D-024237-04
	3	CAAGAGACCAGGAUCGACU	D-024237-19
	4	AGAAUGAGUAUAAGUCCGA	D-024237-20
MDA5 (IFIH1)	1	GAGAAUAACUCAUCAGAAU	D-013041-01
	2	GAAAUCAUCUGCAAAUGUG	D-013041-02
	3	GGAAAUGAAUCAGGUGUAA	D-013041-03
	4	GAAUAACCCAUCACUAAUA	D-013041-04
RIG-1 (DDX58)	1	CAGAAGAUCUUGAGGAUAA	D-012511-01
	2	GCACAGAAGUGUAUAUUGG	D-012511-02
	3	AGACAUGGGUAUAGAGUUA	D-012511-03
	4	CAACCGAUAUCAUUUCUGA	D-012511-04
IRF3	1	GCAAAGAAGGGUUGCGUUU	D-006875-01
	2	AUGCACAGCAGGAGGAUUU	D-006875-03
	3	GGGAAGAGUGGGAGUUCGA	D-006875-04
	4	CCAAGAGGCUCGUGAUGGU	D-006875-05
IRF7	1	GGAAGCACUUCGCGCGCAA	D-011810-03
	2	GAGAGUGGCUCCUUGGAGA	D-011810-04
	3	GACAUCGAGUGCUUCCUUA	D-011810-05
	4	CGAGCUGCACGUUCCUAUA	D-011810-06

TBK1	1	GAACGUAGAUUAGCUUAUA	D-003788-01
	2	UGACAGAGAUUUACUAUCA	D-003788-02
	3	UAAAGUACAUCCACGUUAU	D-003788-06
	4	GGAUAUCGACAGCAGAUUA	D-003788-07
RELA	1	GGAUUGAGGAGAAACGUAA	D-003533-03

Table S11. Source data for the Figures and Supplementary Figures. It is included as an Excel file.
